# Temperature‐Dependent Kinetics of Plasma‐Based CO_2_ Conversion: Interplay of Electron‐Driven and Thermal‐Driven Chemistry

**DOI:** 10.1002/cssc.202401526

**Published:** 2024-11-20

**Authors:** Aswath Mohanan, Ramses Snoeckx, Min Suk Cha

**Affiliations:** ^1^ CCRC Physical Science and Engineering Division (PSE) King Abdullah University of Science and Technology (KAUST) 4700 King Abdullah University of Science and Technology (KAUST) Thuwal 23955 Saudi Arabia; ^2^ Laboratory for Advanced Fibers Empa Swiss Federal Laboratories for Materials Science and Technology Lerchenfeldstrasse 5 9014 St. Gallen Switzerland

**Keywords:** Plasma Chemistry, Kinetics, Gas-phase reactions, Radical Reactions, Sustainable Chemistry

## Abstract

The transformation of CO_2_ into chemical building blocks for various industries is considered a key technology in a net‐zero energy future. To realize this, plasma discharges are one of the most promising approaches thanks to their electron‐driven reactions and high operational flexibility. Most studies focused on room‐temperature and vibrationally‐excited discharges, however, lately, the importance of thermal reactions is considered. Therefore, we developed a temperature‐dependent plasma‐chemical reaction mechanism to investigate the temperature dependence of plasma‐based CO_2_ conversion. Here, we present the various effects of thermally‐driven reactions on the CO_2_ conversion as a function of the gas temperature and specific energy input. Our analysis pinpointed the key reactions controlling the plasma‐based CO_2_ conversion, shifting from an electron‐driven to a thermal‐driven regime. Additionally, we used the mechanism to verify the theoretical upper boundary of the process’ energy efficiency, and discussed how our findings could lead to the further development and optimization of plasma discharges for efficient CO_2_ conversion in the future.

## Graphical abstract

The transformation of CO_2_ into chemical building blocks is essential for a net‐zero energy future. Plasma‐chemical processing offers promising potential to achieve this. Here, we explore a temperature‐dependent plasma‐chemical reaction mechanism, revealing a shift from electron‐driven to thermal‐driven CO_2_ conversion regimes. Key reactions and their impact on process efficiency are analyzed to guide the development of optimized plasma processes.

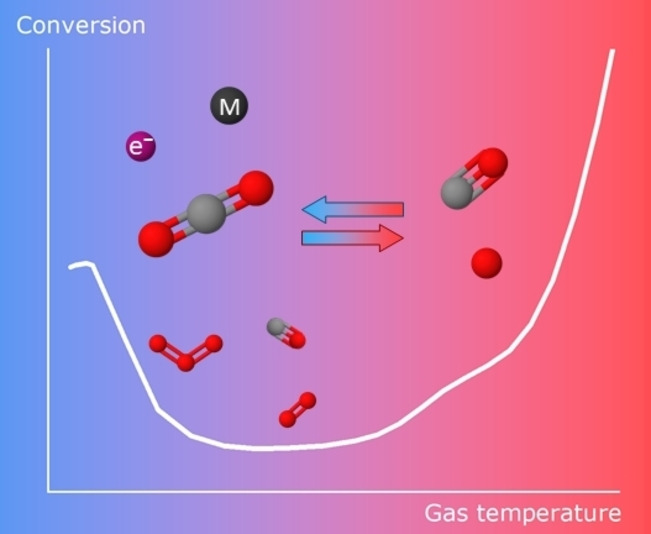

## Introduction

In nature, carbon dioxide (CO_2_) is the primary source of carbon for biochemical processes and it serves as a crucial part of the carbon cycle that encompasses nearly all life on Earth. However, the increased anthropogenic CO_2_ emissions from burning fossil fuels, industrial processes, and agriculture in combination with the destruction of natural carbon sinks through modified land use, are disturbing the natural carbon cycle.[^1^, ^2^, ^3^] This imbalance is causing an unprecedented increase in atmospheric CO_2_ concentrations, which in turn is the main cause of current global warming and ocean acidification.^[4]^ If kept unchecked, these developments will lead to irreversible effects on our climate and environment, and form a major threat to society.^[4]^

In the short‐term (<2050), a transition to net‐zero energy systems is essential to prevent a further increase in atmospheric CO_2_ concentrations.^[4]^ In the long term (>2050), technologies that can capture and store or transform CO_2_ are required to remove the excess CO_2_ from the atmosphere.^[4]^ The captured CO_2_ can then become the carbon source for an industrial carbon cycle (referred to as the circular carbon economy) following the cradle‐to‐cradle principle.[^5^, ^6^, ^7^] To this end, a wide variety of technologies is being explored, such as thermo‐, electro‐, photo‐, and biochemical approaches, both with and without catalysts, and all their possible combinations.[^7^, ^8^] One technology that is of particular interest, thanks to its flexibility of operation and its capability to rely on (renewable) electricity, is plasma–electrical discharges.^[8]^

CO_2_ plasmas have practical applications in various domains, including CO_2_ lasers,^[9]^ astrophysical observations,^[10]^ surface treatment processes on carbon‐containing substrates,[^11^, ^12^] polymer deposition,^[13]^ and the design of spacecraft shields for planetary atmosphere entry.^[14]^ For the plasma‐based conversion of CO_2_, two main pathways are explored: (i) pure CO_2_ splitting into CO and O_2_, where the CO can be used as a feedstock for Fischer‐Tropsch synthesis,[^8^, ^15^] while the production of oxygen is being considered for future space missions[^16^, ^17^]; and (ii) the combined processing of CO_2_ with hydrogen‐containing gases, such as CH_4_, H_2_ or H_2_O, for the production of syngas and valuable oxygenates (e. g. methanol, formaldehyde, and formic acid) in a single synthesis step.^[8]^

Gaining insight into the temperature‐dependence of the CO_2_/CO plasma chemistry is essential to study and optimize any plasma‐based process with CO_2_ as (co−)reactant or intermediate operating at elevated temperatures. This includes not only gliding arc and microwave discharges, but also plasma‐catalytic discharge reactors used for pure CO_2_ conversion^[8]^ and any type of reforming (e. g. partial oxidation,[^18^, ^19^] dry reforming,[^19^, ^20^, ^21^, ^22^] steam reforming,[^19^, ^23^] and multi reforming^[24]^). Additionally, it is relevant for the increased interest in plasma‐assisted combustion.[^25^, ^26^, ^27^] Therefore, we (i) developed a temperature‐dependent plasma‐chemical kinetic reaction mechanism for pure CO_2_, suitable for plasma‐assisted reforming, catalysis, oxidation/reduction, and combustion processes; (ii) investigated the, up till now neglected, temperature‐dependence of the CO_2_ conversion for elevated gas temperatures (*T*_g_=300–1050 K); and (iii) evaluated the energy efficiency of the plasma process for gas temperatures ranging from colder (e. g. room temperature plasma) to warmer (e. g. so‐called warm plasma) conditions (*T*_g_=200 to 5000 K).

Here, we present the development and validation of our mechanism and highlight the importance of gas temperature, electron‐impact cross section data, and the O‐reactions on the conversion of CO_2_. For the numerical part, we performed zero‐dimensional (0D) simulations with the plasma‐chemical kinetic model KAUSTKin.[^28^, ^29^] While for the validation of our mechanism, we obtained experimental data utilizing an atmospheric pressure temperature‐controlled dielectric barrier discharge (DBD) reactor.[^30^, ^31^] Gas chromatography (GC) was used to monitor the conversion of CO_2_ (up to *T*_g_=1050 K), and Fourier‐transform infrared (FTIR) spectroscopy to analyze the production of ozone (O_3_) (up to *T*_g_=600 K). Our plasma‐chemical reaction mechanism for CO_2_ represents a substantial advancement over existing mechanisms by integrating several key improvements. These include updated, detailed electron impact cross‐sections for dissociation and ionization, the inclusion of the electronic excited state CO(a ^3^Π) with corresponding updated reactions, and a comprehensive ozone formation mechanism. While some of these features have been individually incorporated in prior studies, collectively, these enhancements create a temperature‐dependent mechanism that closely aligns with experimental observations. Following the successful validation of our model, a sensitivity analysis and a chemical analysis provided valuable insights into the effect of the gas temperature on the reaction pathways and species involved. Lastly, by evaluating the energy efficiency of the system with extended simulations, we revealed the distinctive temperature regimes for the CO_2_ conversion and discussed their implications for the future advancement and development of efficient plasma‐based CO_2_ conversion processes.

## Results and Discussion

### Temperature Dependence of Plasma‐Based CO_2_ Conversion

Due to the stability of CO_2_, breaking the OC=O bond requires significant dissociation energy (5.51 eV/molecule).^[8]^ Plasmas can supply this energy in different ways, through heat (e. g. thermal plasmas), highly energetic electrons (e. g. cold non‐thermal plasmas), or a combination of both (e. g. warm non‐thermal plasmas). In all cases, the gas temperature influences the reaction rates and, hence, the overall chemistry. To assess the temperature dependence of the plasma‐based CO_2_ conversion process, its performance with respect to the conversion of CO_2_ into CO, O_2_, and O_3_ was investigated for three different specific energy inputs (SEI=0.35, 0.70 and 1.39 eV/molecule, defined by the ratio of the discharge power, i. e. *P*_dis_=5, 10, and 20 W, to the volumetric flow rate at standard conditions, i. e. 200 sccm) and varied gas temperatures (*T*_g_=300 to 1050 K). To evaluate the temperature dependence, the CO_2_ was preheated to a set gas temperature (*T*_g_) before entering the discharge section of the reactor (for more details see the Methods section and the Supporting Information (SI)).

Although the results presented here are based on preheating the gas before it reaches the plasma zone, it is important to note that our observations and the developed plasma‐chemical kinetic mechanism can be directly applied to other discharge types (e. g. microwave and gliding arc), where the elevated gas temperature results from internal heating mechanisms such as joule heating, electron impact heating, vibrational‐translational (V−T) relaxation, exothermic reactions, and others. The advantage of this approach is that, by using external gas heating via an electric furnace in combination with a non‐thermal plasma with minimal heat generation, we achieve independent control over both the gas temperature and discharge parameters.

**Figure 1:**
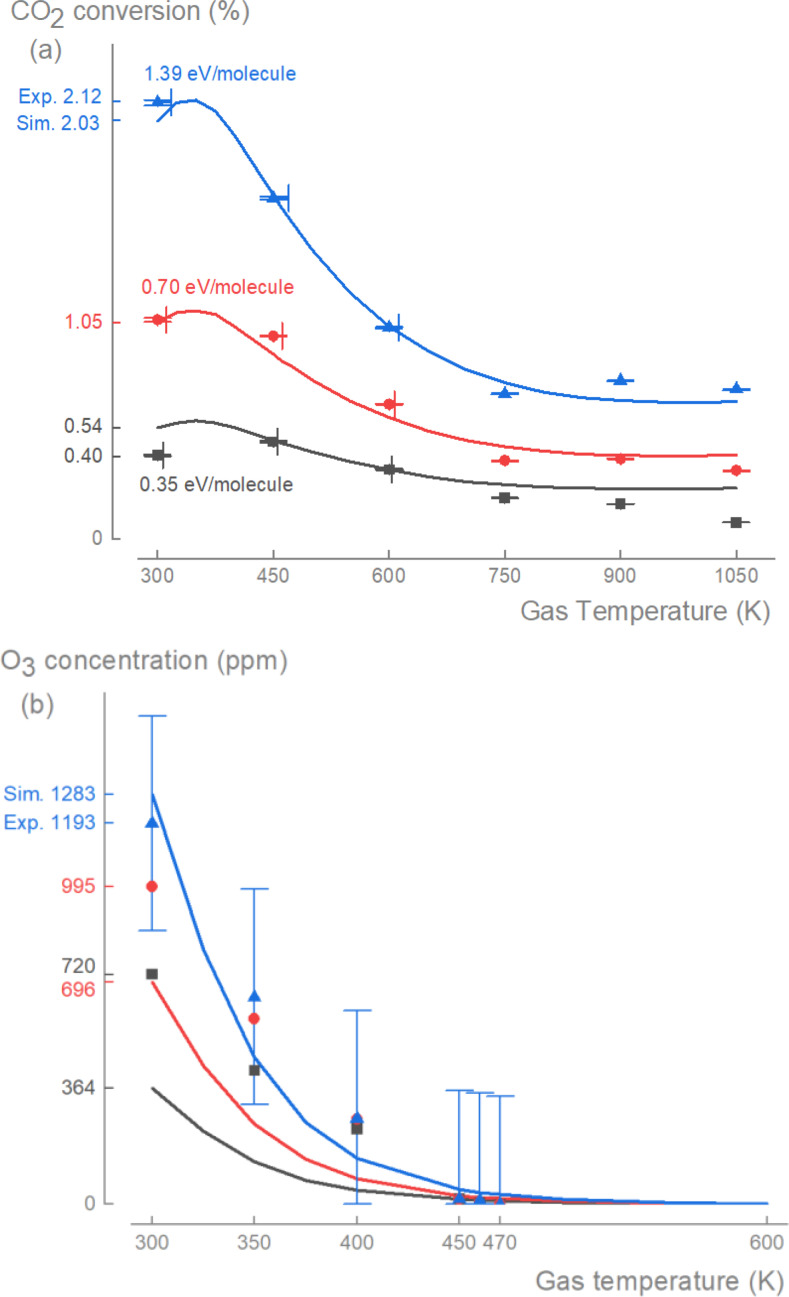
Negative effect of gas temperature on CO_2_ conversion and O_3_ production. The measured (symbols) conversion of CO_2_ (a) and concentration of O_3_ (b) decreased with higher gas temperature and increased with higher specific energy input (SEI). The simulations with our mechanism (solid lines) captured these different trends for the studied range of *T*_g_=300–1050 K and SEI=0.35–1.39 eV/molecule. The error margin in (b) is ±337 ppm for all three SEI cases, for clarity error bars in (b) are only shown for SEI=1.39 eV/molecule.

The experimentally obtained CO_2_ conversion showed a consistent decline across all specific energy inputs (SEI=0.35, 0.70 and 1.39 eV/molecule) as the gas temperature increased from *T*_g_=300 to 750 K, and then remained relatively unchanged as the gas temperature increased further to *T*_g_=1050 K (Figure 1a). As expected in most plasma processes, the conversion increased proportionally to the energy input.^[32]^ The standard deviation of successive measurements was 1.4 %, as indicated by the vertical error bars. Heating due to the discharge led to a small increase in the gas temperature (which was more pronounced with higher SEI), as indicated by the horizontal error bars (see the Methods section and SI, for more information). For SEI=0.70 and 1.39 eV/molecule, our mechanism predicted the experimental conversion well within a 20 % deviation (in most cases <10 %). However, at SEI=0.35 eV/molecule, the discharge was unstable (especially at higher *T*_g_), likely leading to the larger deviations between the simulations and the experiments for *T*_g_≥600 K. Interestingly, our simulations exhibited a maximum CO_2_ conversion at *T*_g_=350 K. With increased gas temperature from *T*_g_=300 K to 350 K, there was a relative increase in CO_2_ conversion of ~5–6 %. However, from *T*_g_=350 K to 750 K, CO_2_ conversion dropped significantly by ~54–64 %, which matched the experimentally observed decrease of ~51–67 %. Hence, for the non‐thermal plasma conditions studied, a small increase in gas temperature up to *T*_g_=350 K had a minor positive effect, while a further increase had a detrimental effect on the CO_2_ conversion and thus, the process efficiency. Furthermore, these results indicate that the reactions responsible for the thermal dissociation of CO_2_ are not yet active in this temperature regime (*T*_g_≤1050 K).

An often neglected aspect of plasma‐based CO_2_ conversion is the production of O_3_. This temperature‐sensitive process^[31]^ can significantly impact CO_2_ conversion and provide valuable information to validate our reaction mechanism. Similar to the CO_2_ conversion, the O_3_ concentration declined as the gas temperature increased (Figure 1b). O_3_ was detected up to *T*_g_=470 K, but at higher gas temperatures, the signal became indistinguishable from background noise. These observations are consistent with the dissociation of O_3_ at elevated temperatures as observed in O_2_ discharges by Bang et al.^[31]^ For additional information on the O_3_ measurements, see the Methods section and SI. With modified third‐body efficiencies (see Methods section and SI), our mechanism captured the trend of decreasing O_3_ concentration with increased gas temperature, within the experimental error margin of ±337 ppm. At *T*_g_=600 K, the concentration of O_3_ became negligible in the simulations. These results indicated that in non‐thermal plasmas with a low gas temperature, the reactions involved in the production and decomposition of O_3_ play an important role for the CO_2_ conversion; whereas for plasmas with a higher gas temperature (e. g. warm non‐thermal plasmas and thermal plasmas), their role is minimal.

The good agreement between our simulation results and experimental data for both the conversion of CO_2_ (Figure 1a) and the production of O_3_ (Figure 1b) validated several important aspects of our newly developed reaction mechanism: (i) the improved reaction rate coefficients and third‐body efficiencies for the O_2_ chemistry, (ii) the modification of the back reaction CO(a ^3^Π)+O, and (iii) the newly assigned combination of cross section data (see Methods section and SI). Therefore, we employed this reaction mechanism to investigate the underlying plasma‐chemical pathways of the CO_2_ conversion process and its temperature dependence.

### Insights in the Chemistry Through Chemical Analysis

#### Main Reactions

To analyze the primary reaction pathways, we integrated the instantaneous reaction rates over the entire duration of the simulation (see SI). This analysis revealed significant shifts in the pathways as a function of the gas temperature, whereas the influence of the specific energy input was minimal. This observation aligns with findings for other gases, such as H_2_,[^28^, ^29^] NH_3_ ^[30]^ and O_2_.^[31]^ In plasma discharges, CO_2_ decomposition is initiated by electron impact dissociation of CO_2_ producing ground state O (O(^3^P)) and ground state CO (CO(^1^Σ^+^)) (R1) or electronic excited CO(a ^3^Π) (R2.[^33^, ^34^]
(R1)

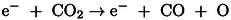



(R2)

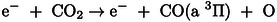




Together, these reactions account for 78–97 % of CO_2_ consumption depending on *T*_g_ (Figure S14b). The contribution of the primary process, R1, decreased from 96 to 67 % as the gas temperature increased from *T*_g_=300 to 900 K, while R2’s contribution increased from 1.3 to ~11 %.

Other significant contributors to CO_2_ consumption are electron impact ionization (R3) and dissociative electron attachment (R4.
(R3)

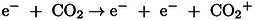



(R4)

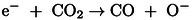




The contribution of R3 increased from 1.4 % at *T*_g_=300 K to ~20 % at *T*_g_=900 K. In contrast, the contribution of R4 decreased from 2 % at *T*_g_=300 K to 1.2 % at *T*_g_=450 and became negligible at higher temperatures.

Besides electron impact processes with CO_2_, the electron impact excitation of CO (R5) must be considered, as it transforms ground state CO into the more energetic electronically excited CO(a ^3^Π). This process alters the ratio of ground state to electronic excited CO, which influences the reaction pathways (see below).
(R5)

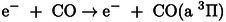




At *T*_g_=300 K, R5 is responsible for 89, 94, and 96 % of CO(a ^3^Π) production at SEI=0.35, 0.70 and 1.39 eV/molecule, respectively. As the gas temperature rises, its role decreases to 20, 28, and 38 % at *T*_g_=1050 K.

Next, these main species (CO, CO(a ^3^Π), and O) produced by the plasma can further interact with each other, potentially inhibiting the conversion process through the back‐production of CO_2_.[^35^, ^36^, ^37^]
(R6)

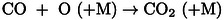



(R7)

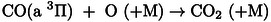



(R8)

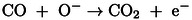



(R9)

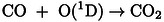




The recombination reaction of CO and O (R6) emerged as the primary route for CO_2_ production, with its contribution increasing sharply as the gas temperature rose from *T*_g_=300 to 600 K (Figure S14a), but decreasing as the specific energy input increased. The recombination of the excited state CO(a ^3^Π) with O (R7) was the second most significant process. At *T*_g_=300 K, R7’s impact (>56 %) even surpassed R6. However, R7’s contribution decreased significantly at *T*_g_=450 K, whereas that of R6 grew. Conversely, the contribution of R7 increased with increasing specific energy input due to the higher production of CO(a ^3^Π), leading to a reduced role for R6. Furthermore, at *T*_g_=300 K, R8’s contribution decreased from 15 to 7 % as the specific energy input increased from SEI=0.35 to 1.39 eV/molecule and its role diminished rapidly with rising temperature (<2.5 % at 450 K). Finally, R9 had a minor influence (<2 %) at *T*_g_=300 K for both SEI=0.70 and 1.39 eV/molecule.

In addition to the recombination reactions R6 and R7, other key reactions that significantly influence the plasma‐based CO_2_ conversion process involve O, O_2_, and O_3_.
(R10)

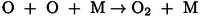



(R11)

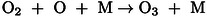



(R12)

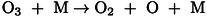



(R13)

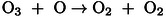



(R14)

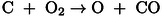




**Figure 2:**
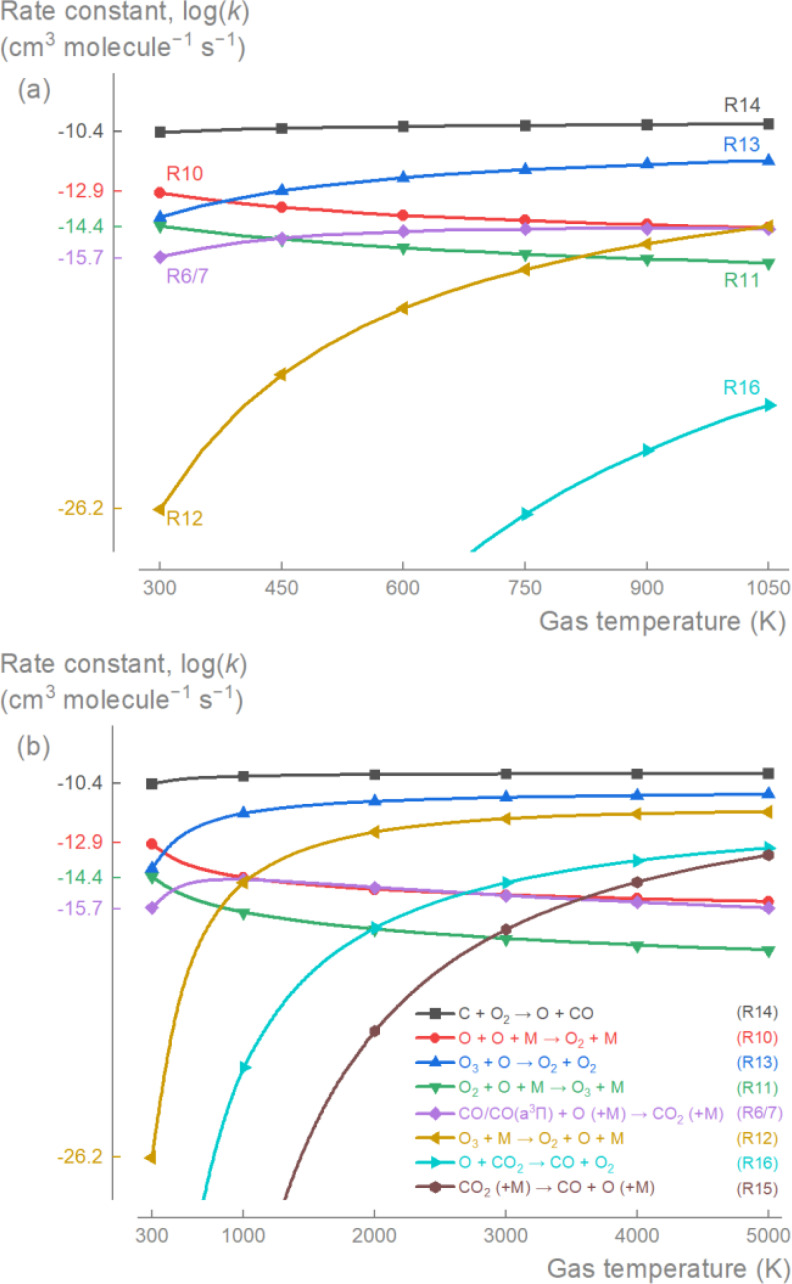
Variation of reaction rate constants (*k*) with gas temperature (*T*_g_) for main reactions that determine plasma‐based CO_2_ conversion process. For the investigated temperature range (a), as *T*_g_ increases, *k*_R10_ and *k*_R11_ decrease, while *k*_R12_ and *k*_R14_ increase, resulting in increased O production. The elevated O density coupled with the increased *k*_R6_, facilitates the back reaction of O to CO_2_ rather than to O_2_ (or O_3_), and thus limiting the plasma‐based CO_2_ conversion process. For higher temperatures (b), as found in warmer plasmas, the strong increase of *k*_R16_ and *k*_R15_ can significantly affect the process at *T*_g_>2500 K through both the direct decomposition of CO_2_ (R15) and the simultaneous consumption of O radicals and CO_2_ producing CO and O_2_ (R16).

The decrease in CO_2_ conversion with increased gas temperature is primarily due to the increasing reaction rate constant of R6 (Figure 2). On the other hand, R10, R11, and R13 consumed the available O radicals, which positively impacts the CO_2_ conversion by limiting their recombination into CO_2_ via R6. As the gas temperature increased, the decreasing reaction rate constants for R10 and R11 resulted in lower O radical consumption. At *T*_g_≥600 K, R12 (the reverse reaction of R11) contributes additional O radicals. Although the reaction rate constant for R13 increases with temperature, the decreased production (R11) and increased decomposition (R12) of O_3_ particularly for *T*_g_≥450 K, results in lower concentrations of O_3_. Thus, R13’s consumption of O radicals rapidly diminishes with increased temperatures. Lastly, at *T*_g_≥600 K, R14 also provided O radicals due to its high reaction rate coupled with the increased availability of C from the dissociative electron ion recombination of CO_2_^+^ at higher *E*/*N*. Therefore, as the gas temperature increased, the increased O radical production rate along with the elevated reaction rates of R6 and R7 promoted CO_2_ production, leading to decreased CO_2_ conversion as more CO recombines into CO_2_. At low *T*_g_, CO_2_ conversion is maximized because the rate constant of R13 is comparable to those of R10 and R11, while surpassing R12 and R6. This results in ample O_3_ concentration and more O radical consumption via reactions R10, R11 and R13 rather than R6, thereby minimizing CO to CO_2_ recombination.

It must be mentioned that the two thermal reactions for CO_2_ dissociation (R15 and R16) are insignificant within the investigated temperature range (*T*_g_≤1050 K.
(R15)

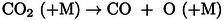



(R16)

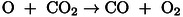




R16 has been considered significant for non‐thermal plasma‐based CO_2_ conversion due to its favourable thermodynamics.^[35]^ However, kinetically, it is evident that this reaction only becomes important at high gas temperatures, *T*_g_>2000 K (Figure 2b), while R15 requires even higher gas temperatures, *T*_g_>3000 K (Figure 2b). These findings aligns with recent observations regarding so‐called warm plasma‐based CO_2_ conversion (see Section 2.4).[^35^, ^38^, ^39^]

Our analysis reveals that the non‐thermal plasma‐based decomposition of CO_2_ is controlled by the available O radicals. More specifically, the competition between (i) recombination reactions (R6 and R7) that produce CO_2_ and thus hinder the overall CO_2_ decomposition process, and (ii) the two‐step production of O_3_ (R10 and R11) and its decomposition into O_2_ (R13). At low temperatures, the production (R11) and decomposition (R13) of O_3_ consume the majority of the O radicals due to their higher rate constants compared to the recombination reactions (R6 and R7) (Figure 2). However, as the gas temperature rises, O_3_ production decreases while its decomposition through R12 increases. This resulted in the increased O radical production rate, enhancing the recombination reactions (R6 and R7) and negatively impacting CO_2_ conversion (Figure 1a).

#### Reaction Pathways

**Figure 3:**
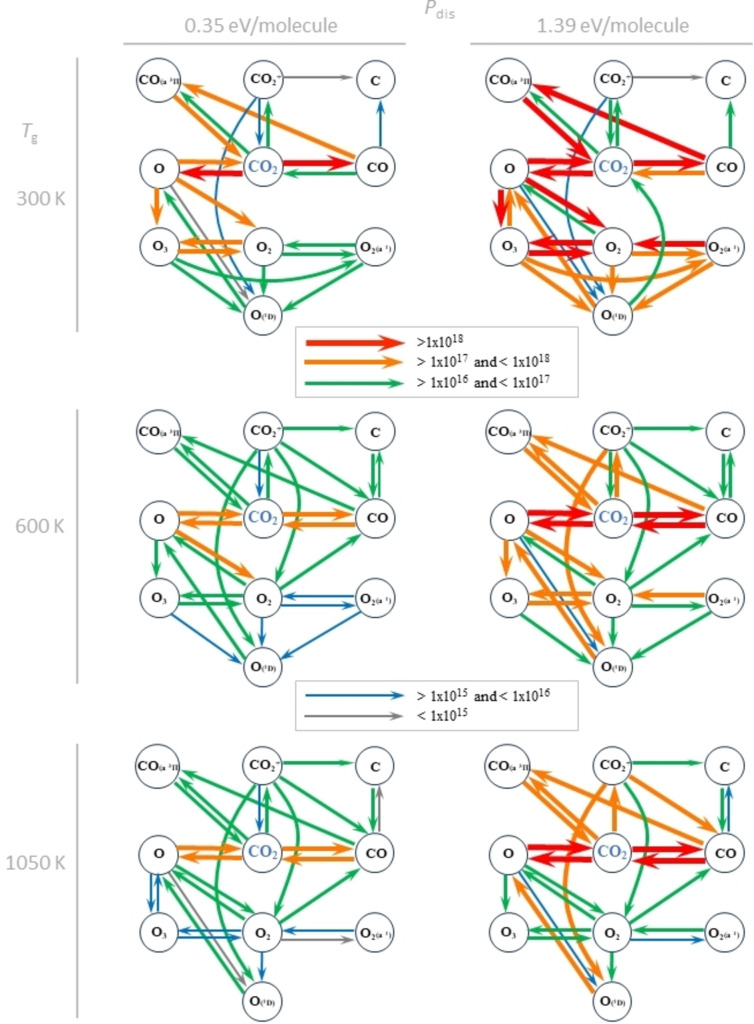
Reaction scheme visualizing production pathways between key species involved in plasma‐based CO_2_ conversion process. The production pathways (gray<blue < green < orange < red arrows) are visualized for *T*_g_=300, 600, and 1050 K (top to bottom) and SEI=0.35 and 1.39 eV/molecule (left to right). The diagram provides valuable insights into the complex interplay of factors affecting the plasma‐based CO_2_ conversion. As *P*_dis_ increases, there is a clear enhancement in production activity and hence an increase in CO_2_ conversion. Conversely, as *T*_g_ increases, there is a reduction in the production activity of species and hence a decrease in CO_2_ conversion.

Reaction pathway diagrams involving key species (Figure 3) visually illustrate the trends in CO_2_ conversion relative to gas temperature and specific energy input. From these diagrams and extensive chemical analysis data (see Figures S10–S20 in SI), we can derive three key observations: (i) an increased net CO_2_ decomposition and CO production for higher specific energy inputs due to an increased electron density; (ii) a decreased net CO_2_ decomposition, CO production, and O_3_ production for higher gas temperatures, due to the dominant consumption of O radicals by recombination reactions R6 and R7, rather than reactions that produce O_2_ (R10 and R13) and O_3_ (R11); (iii) the role of excited species decreases with elevated gas temperatures and increases with higher specific energy input.

At *T*_g_=300 K, a significant portion of the converted CO is excited to CO(a ^3^Π) via R5, while the majority of the O radicals is transformed to O_2_ and O_3_ (R10, R11, and R13). Consequently, the overall production of CO_2_ remains low. Notably, since the electron impact excitation of CO (R5) dominates over CO recombination (R6), the recombination of CO(a ^3^Π) (R7) emerges as the primary contributor to CO_2_ production. However, as the gas temperature increased to *T*_g_=600 K, there is a significant increase in CO_2_ production from R6, displacing R7. Simultaneously, O_3_ production via R12 decreases and a new pathway for O production from O_2_ via R14 emerges, leading to a substantial decrease in CO_2_ conversion. Furthermore, as the gas temperature continues to rise, O is produced from O_3_ through R12. Lastly, the near constant CO_2_ conversion for *T*_g_>750 K can be explained based on the relatively constant overall reaction rates of the main CO_2_ decomposition (R1–3) and production (R6) reactions (Figure S13) at these temperatures.

#### Sensitivity Analysis

**Figure 4:**
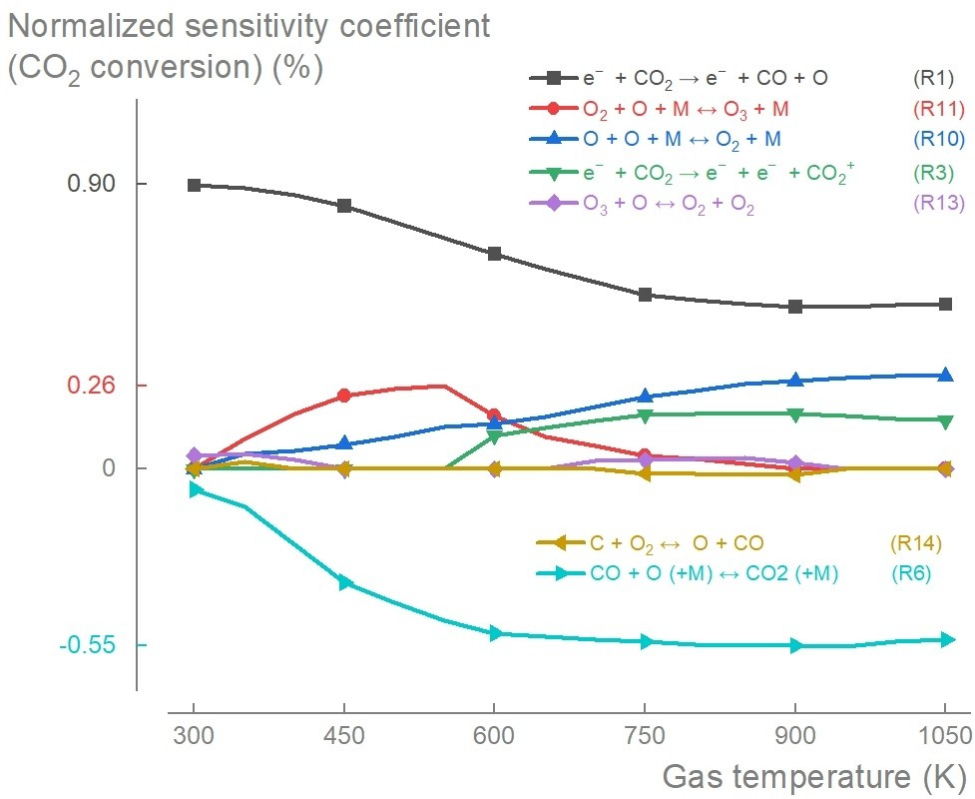
Normalized sensitivity coefficients (*S̃*_i,j_) with respect to CO_2_ conversion (for SEI=1.39 eV/molecule). Reactions that dissociate CO_2_ (R1, R3) and those that produce O_2_ (R10, R13) and O_3_ (R11) exhibit a clear positive sensitivity with respect to the CO_2_ conversion, while the recombination reaction that produces CO_2_ (R6) exhibits a negative sensitivity.

To better understand the rate‐limiting steps of the process (i. e. reactions that have a significant effect on the overall process when their rate coefficient is changed), we conducted a (brute force) sensitivity analysis[^40^, ^41^] by calculating the normalized sensitivity coefficient (*S̃*_i,j_):

(1) $${\tilde{S}}_{i,j}=\frac{k_{j}}{X_{i}}\frac{\partial X_{i}}{\partial k_{j}}=\frac{\partial lnX_{i}}{\partial lnk_{j}}$$

where *k*_j_ is the fractional change in the rate coefficient of reaction *j* and *X*_i_ the fractional change in the concentration of species *i*. To evaluate the sensitivity of the plasma‐based CO_2_ conversion, we investigated *i*=CO_2_ with a threshold criterion |*S̃*_i,j_|>0.1 (Figure 4). A positive value of *S̃*_i,j_ indicates a positive effect of the reaction on the CO_2_ conversion, while a negative value indicates a negative effect.

The sensitivity analysis identified seven rate‐limiting reactions, supporting the main observations: (i) the key role of R1 as an initiation step, (ii) the significance of ionization at higher temperatures, and (iii) the competition for O radicals.

The electron impact dissociation of CO_2_ (R1) emerged as the primary rate‐limiting step, aligning with its pivotal role in facilitating the CO_2_ conversion. Its normalized sensitivity coefficient decreased from 0.88 at *T*_g_=300 K to 0.50 at *T*_g_=900 K, with a slight increase to 0.51 at *T*_g_=1050 K. At higher gas temperatures, particularly at *T*_g_≥600 K (with *E/N* exceeding 220 Td), the ionization reaction (R3) became prominent in aiding the CO_2_ conversion. The analysis also highlighted the competition for O radicals between CO recombination (R6), O_2_ production (R10), and O_3_ production (R11). The normalized sensitivity coefficient of R10 consistently increased to 0.29 at *T*_g_=1050 K, while that of R11 increased to 0.26 at *T*_g_=550 K but declined again with further increased gas temperatures. R6 was the main rate‐limiting step responsible for restraining the CO_2_ conversion. Its coefficient decreased rapidly from −0.06 at *T*_g_=300 K to −0.51 at *T*_g_=600 K, reaching a minimum of −0.55 at *T*_g_=900 K.

### Energy Efficiency

**Figure 5:**
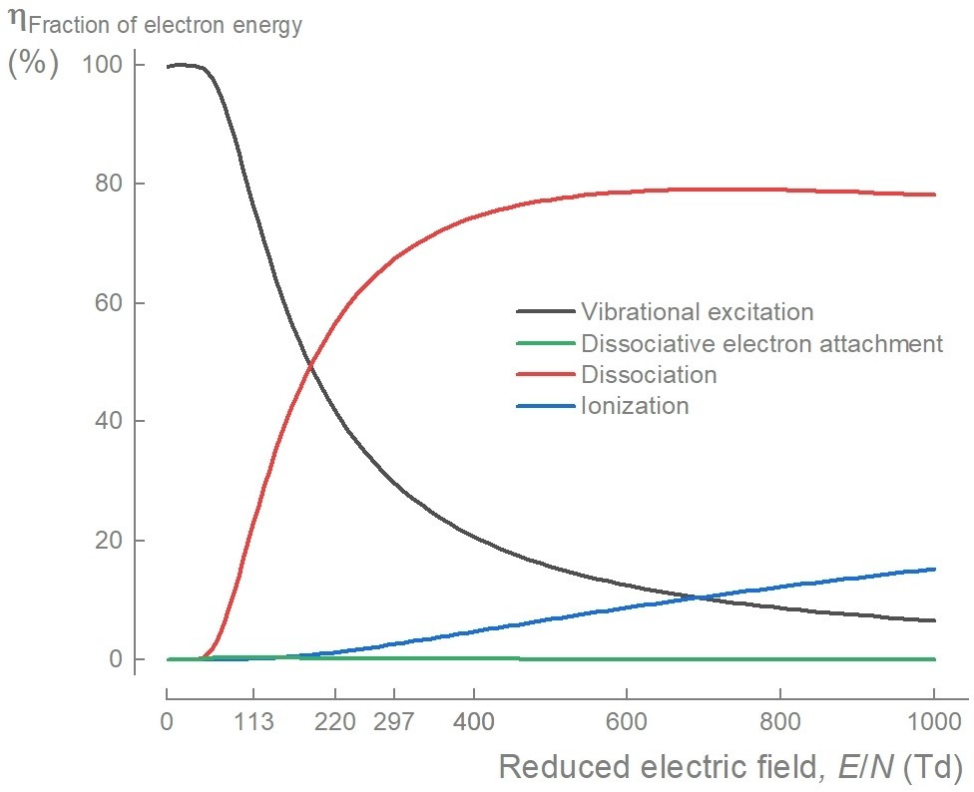
Changes in fraction of energy transferred to various electron impact processes with CO_2_. The energy transferred to vibrational excitation, dissociation, ionization, and dissociative electron attachment calculated with Bolsig+^[42]^ as a function of reduced electric field (*E*/*N*) using the cross section data presented in this work. There is a clear shift from vibrational excitation to dissociation and ionization with increased *E*/*N*. Note that some higher electronic excitation processes are not included.

Understanding plasma‐based CO_2_ conversion requires knowledge of how the electron energy is distributed, as the process relies on electrons to initiate the production of reactive species, like radicals and excited states. The distribution of electron energy across various electron impact processes is dependent on the reduced electric field (*E*/*N*). The electron energy loss fraction (Figure 5) represents the proportion of energy transferred from the electrons to CO_2_ molecules during various collisions. Note that some higher energy electronic excitations, which would be pronounced at high *E*/*N*, are absent in the mechanism used.

At low *E*/*N* (<100 Td), vibrational excitation is the dominant process. This has been extensively discussed since the 1960′s as a potentially efficient conversion pathway through vibrational ladder climbing,^[8]^ however, to‐date its practical feasability remains unproven. As *E*/*N* increases, energy transfer to dissociation increases and this becomes the primary process above 200 Td. In our experiments, *E*/*N* increased with increased gas temperature from 113 Td at *T*_g_=300 K to 297 Td at *T*_g_=900 K, followed by a slight decrease to 277 Td at 1050 K (Figure S5). Hence, one might have expected an increased CO_2_ conversion due to the increased *E*/*N* as the gas temperature increased. However, as discussed above, while electron impact dissociation initiates the plasma‐based CO_2_ conversion process, the final conversion is controlled by the thermally‐governed reactions with O radicals. Thus, the negative effect on the conversion of these thermally‐governed reactions with increasing gas temperature outweighs the benefits of increased fraction of electron energy going to electron impact dissociation. Note that our simulations predicted a minor increase in the CO_2_ conversion due to increased *E*/*N* at low gas temperatures (*T*_g_<350 K) (Figure 1, see SI Section 4.2 and Figure S21 for a comparison of simulations with fixed *E*/*N*).

To assess the performance of the plasma‐based CO_2_ conversion process in relation to the reaction enthalpy, we computed the energy efficiency (*η*).

(2) $$\eta =\frac{\chi_{Total}\left(\%\right)\times \Delta H_{R}\left(kJmol^{-1}\right)}{SEI\;\left(kJ\;mol^{-1}\right)+\;\Delta H_{gas}\;\left(kJ\;mol^{-1}\right)}$$

where *χ*_Total_ is the total conversion of CO_2_, Δ*H*_R_ is the reaction enthalpy (as function of *T*_g_), SEI is the specific energy input defined by the ratio of the discharge power to the volumetric flow rate at standard conditions, and Δ*H*_gas_ is the energy required to heat the gas.

**Figure 6:**
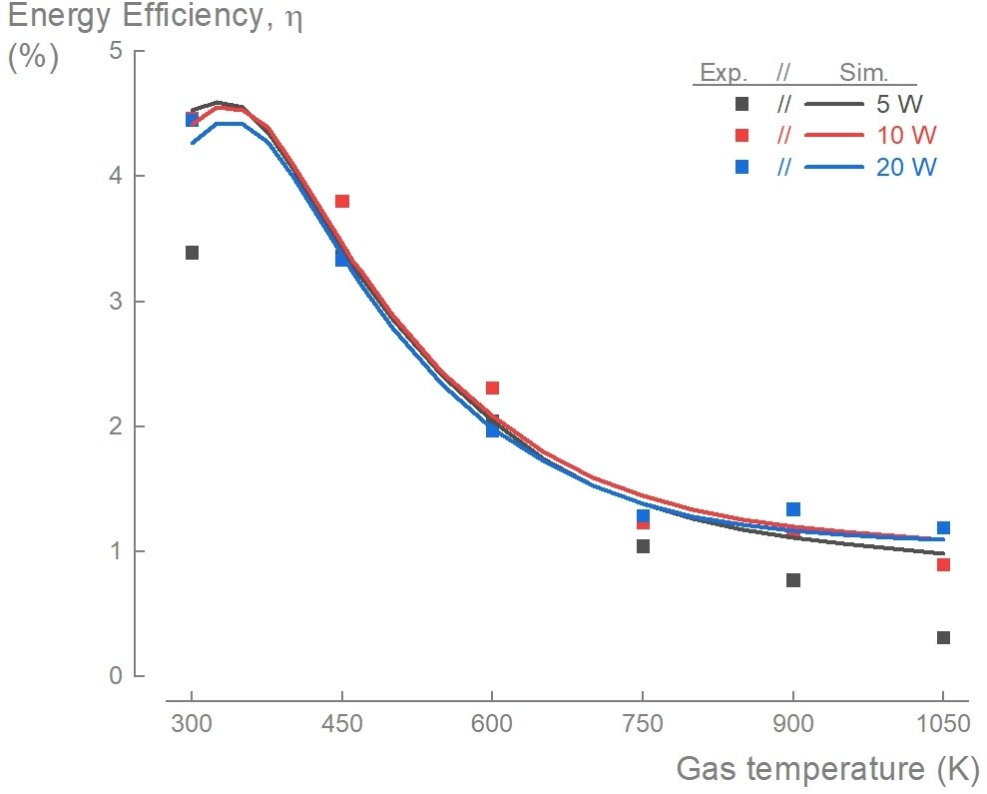
Energy efficiency of plasma‐based CO_2_ conversion process decreased with gas temperature and is independent of specific energy input. The measured (symbols) energy efficiency of the plasma‐based CO_2_ conversion process decreased with higher gas temperature and remained comparable for the different discharge powers. The simulations with our mechanism (solid lines) captured these trends for the studied range of *T*_g_=300–1050 K and SEI=0.35–1.39 eV/molecule.

The energy efficiency exhibited a similar downward trend to CO_2_ conversion as the gas temperature increased. However, it showed no significant dependence on the specific energy input (most results from the experiments fall within a ±12 % deviation) (Figure 6). This suggests that, despite a non‐linear relation between *χ*_Total_ and the sum of SEI and Δ*H*_gas_ (as SEI was comparable to Δ*H*_gas_), the increase in *χ*_Total_ with respect to the specific energy input at each temperature considered effectively balanced out the sum of SEI and Δ*H*_gas_. The deviation at SEI=0.35 eV/molecule could be attributed to the previously discussed instability of the discharge during the experiment. At SEI=0.70 eV/molecule, the energy efficiency decreased from 4.5 % at *T*_g_=300 K to 0.9 % at *T*_g_=1050 K (Figure 6), mirroring the decrease in CO_2_ conversion from 1.1 % to 0.3 % (Figure 1). Like the results for the conversion, our simulations well captured the trends observed in the experiments. It is important to note that energy efficiency is inversely proportional to the energy required to heat the gas, Δ*H*_gas_. As the gas temperature increases, the energy needed to heat the gas also rises, contributing to the decrease in energy efficiency along with the decrease in conversion. For instance, for SEI=1.39 eV/molecule, the contribution of gas heating increased from 0.05 % of the total energy input (i. e. discharge power and heating power) at *T*_g_=300 K to 21.6 % at 1050 K.

## Implications and Outlook

**Figure 7:**
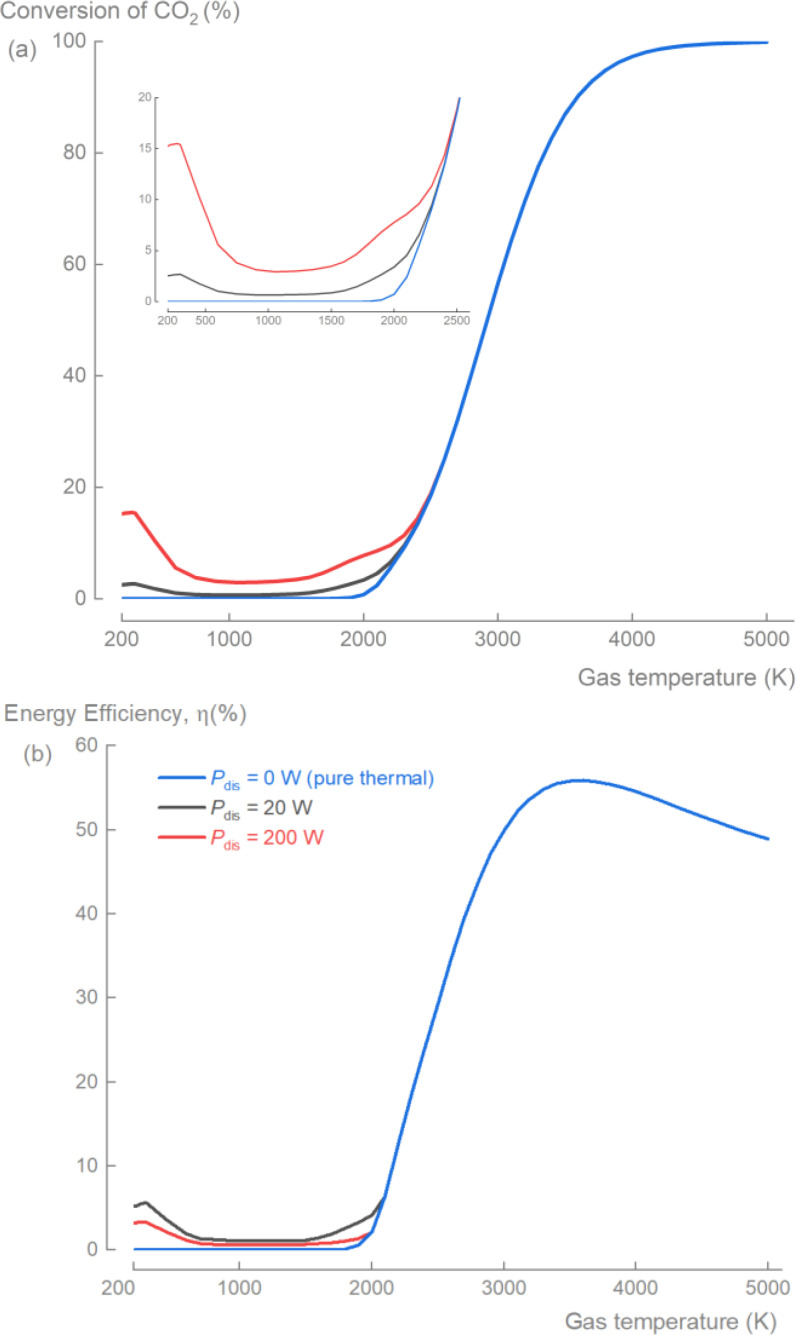
Predicted maximum conversion and energy efficiency of plasma‐based CO_2_ conversion up to *T*_g_=5000 K. Simulated CO_2_ conversion (a) and energy efficiency (b) as a function of temperature for pure thermal (*P*_dis_=0 W) and plasma‐based (*P*_dis_=20 and 200 W, *E*/*N*=200 Td) conversion of CO_2_. Note: Discharges can induce gas heating effects that depend on several factors, including the discharge type, reactor configuration, and process conditions, typically increasing with discharge power. Although, this figure does not directly account for these effects, they can be approximated by evaluating conversion and energy efficiency at the observed or expected overall gas temperature in the specific plasma‐based CO_2_ conversion process.

In this last section, we employed the validated temperature‐dependent CO_2_ mechanism to numerically investigate the plasma‐based CO_2_ conversion for gas temperatures ranging from *T*_g_=200 to 5000 K. With this approach we aimed to decouple the contributions of electron‐driven and thermal‐driven chemistry, and to estimate the maximum potential in terms of the conversion and energy efficiency (Figure 7). To facilitate the comparison of the energy input related to discharge power and thermal heating, discharge power will be used in this section instead of specific energy input (*P*_dis_=20 W corresponds to SEI=1.39 eV/molecule and 200 W corresponds to 13.94 eV/molecule).

Four regimes were identified based on the gas temperature (Figure 7): (i) a discharge‐limited regime (*T*_g_≤300 K), where CO_2_ conversion is primarily governed by the electron‐driven chemistry; (ii) a thermal‐inhibiting regime (300<*T*_g_≤1500 K), where CO_2_ conversion is initiated by the electron‐driven chemistry, but the thermal‐driven chemistry favors the recombination of CO to CO_2_ (R6) causing a reduced CO_2_ conversion; (iii) a thermal‐accelerating regime (1500<*T*_g_≤2500 K), where the CO_2_ conversion is initiated by the electron‐driven chemistry and remains suppressed, but due to a change in the reaction rates of the thermal‐driven chemistry (favoring R16 over R11 and R6) the conversion starts to increase again; and (iv) a temperature‐limited regime (*T*_g_>2500 K), where the CO_2_ conversion is dominated by thermal‐driven chemistry and the impact of electron‐driven chemistry on the final conversion becomes negligible. It is important to note that for self‐sustaining thermal plasma discharges within this regime, where the gas heating is achieved entirely through the applied discharge power, it is challenging to completely separate electron‐induced chemistry from thermal effects. Nonetheless, it is evident that CO_2_ conversion is primarily dictated by the thermal effects. For instance, at *T*_g_>2500 K, the chemistry is entirely thermal‐driven; at this point, even a discharge power of *P*_dis_=200 W has a minimal effect on the CO_2_ conversion. In contrast, in the discharge‐limited regime (*T*_g_≤300 K), increasing the gas temperature had no effect on CO_2_ conversion for a fixed reduced electric field, due to the minimal effect of the thermal chemistry in this regime.

At high gas temperatures, CO_2_ conversion is primarily governed by reactions R15 and R16.^[35]^ Specifically, at *T*_g_=2500 K, the reaction rate constant of R16 surpasses those of R10 and R6, and at *T*_g_=3600 K, R15 also exceeds R10 and R6 (Figure 2b) leading to higher conversions (Figure 7). This temperature‐dependent behavior aligns with experimental observations for so‐called warm plasma‐based CO_2_ conversion and underscores the importance of post‐processing techniques to maintain high conversion and energy efficiency upon cooling the processed gas mixture.^[8]^ For instance, fast gas quenching using nozzles^[43]^ or cooled elements like channels^[44]^ to prevent a continuous drop in conversion upon cooling (Figure 7). Another approach is using solid carbon (C) as a scavenger for O_2_ (via R14)[^45^, ^46^] to limit the backward reaction of R16 by converting the produced O_2_ into CO, thereby shifting the chemical equilibrium in favor of CO_2_ conversion.

If one would consider a plasma process where all the electron energy of the discharge is transferred into gas heating, the theoretical maximum energy efficiency would be around 56 % (Figure 7b). For non‐thermal plasma processes (assuming *T*_g_<2000 K), the predicted maximum energy efficiency is <10 %, consistent with most recent experimental data.[^8^, ^38^] On the other hand, our numerical investigation shows that a high energy efficiency (>50 %) with substantial CO_2_ conversion (>60 %) can be achieved over *T*_g_=3000 K. This suggests that plasmas operating at higher gas temperatures (e. g. warm plasmas like microwave and gliding arc plasmas) are preferable.

This observation could be confirmed further by comparing the effectiveness of allocating the electrical power to either electron‐induced reactions or to pure heating. If we allocated 20 W to (i) electrons (*P*_dis_=20 W), by setting *E*/*N* at 200 Td while maintaining a *T*_g_ of 300 K; and (ii) heat (*P*_heat_=20 W), by increasing the gas temperature to *T*_g_=2300 K, the conversion for the thermal case (*P*_dis_=0 W at *T*_g_=2300 K in Figure 7) is predicted to be four and a half times higher compared to that for the electron case (*P*_dis_=20 W at *T*_g_=300 K in Figure 7). Likewise, to achieve a conversion of 15.4 %, the thermal case requires only *P*_heat_=24 W (*P*_dis_=0 W at *T*_g_=2442 K), whereas the electron case needs 200 W (*P*_dis_=200 W at *T*_g_=300 K). This means the thermal case requires about eight times less energy. Furthermore, for the thermal case to achieve the highest efficiency (55.9 % for *P*_dis_=0 W at *T*_g_=3600 K), only *P*_heat_=65 W is predicted, and to reach 99.8 % conversion, *P*_heat_=80 W is needed. Therefore, plasmas that effectively transfer the electrical energy into heat (*P*_dis_≈*P*_heat_) in the *T*_g_=3000–4000 K range could offer the most efficient CO_2_ conversion. This gas temperature range aligns well with reported efficiencies (40–60 %) from microwave discharge experiments.^[8]^ This suggests that these processes are primarily thermally‐driven rather than relying on vibrational ladder climbing.

At elevated temperatures, the reaction rate constants of both the CO_2_ conversion‐promoting reactions (R1–4, R10, R11, and R13 for *T*_g_<1500 K, and additionally R15 and R16 for *T*_g_>1500 K) and the CO_2_ conversion‐inhibiting reactions (R6 and R7) are significantly high.^[35]^ Hence, the observed CO_2_ conversion is the result of a delicate balance between these competing reactions. To enhance conversion rates, this balance must be shifted in favor of the conversion‐promoting pathways. One approach to achieve this is by removing oxygen through scavengers,[^36^, ^45^, ^46^, ^47^] extraction using membranes,^[48]^ or in‐situ chemical oxygen trapping,[^37^, ^49^] which can limit the back conversion via R6 and R7. Additionally, the use of catalysts^[50]^ offers another effective strategy to further offset this balance and improve the overall CO_2_ conversion efficiency.

Although challenging, achieving the desired mid‐temperature window (*T*_g_=3000–4000 K) with spatial uniformity may be possible through the use of non‐thermal plasmas with elevated temperatures (such as so called warm plasmas). By carefully adjusting power input and the reduced electric field, it could be feasible to generate a high electron density without raising the gas temperature beyond the optimal range. Pulsed discharges could also be employed with a feedback system to fine‐tune the frequency, location, and energy per pulse, allowing rapid gas heating while maintaining spatial uniformity. A feedback system using laser diagnostics or optical emission spectroscopy could monitor temperature and species concentrations in real time, enabling continuous adjustment of discharge conditions to achieve uniformity. However, a combination of experiments and simulations is likely required to fully assess the viability of these approaches and determine the optimal conditions.

## Conclusions

Our newly developed temperature‐dependent mechanism provided significant improvements over existing room‐temperature mechanisms, effectively capturing the experimental trends as a function of gas temperature and specific energy input. This newly developed mechanism differentiates itself from existing ones by combining five major changes: (i) assigning the cross‐sections suggested by Polak and Slovetsky^[33]^ for the various electron impact CO_2_ dissociation reactions; (ii) using detailed experimental cross‐sections for the electron impact ionization of the main gas components CO_2_,^[51]^ CO,^[52]^ and O_2_;^[53]^ (iii) including reactions with the electronic excited state CO(a ^3^Π); (iv) considering third‐body efficiencies of 0.5 for CO_2_ and 0.25 for CO with respect to reaction O_2_+O+M↔O_3_+M; and (v) modifying the reaction rate of the recombination reaction O+CO(a ^3^Π)(+M)→CO_2_(+M). We believe, this mechanism will further enhance the understanding of the underlying physicochemical processes and reactions for various temperature‐dependent plasma‐assisted processes containing CO_2_, such as plasma‐assisted catalysis, reforming, oxidation, reduction, and combustion. Since the fundamentals of plasma‐based CO_2_ conversion remain consistent–initiated by electron chemistry and governed by thermal chemistry–the developed mechanism is applicable to any plasma source with a homogeneous temperature distribution. In cases of non‐homogeneous temperature distribution, the mechanism can still be used to determine local effects.^[54]^ This versatility extends its applicability to other plasma sources, especially for those where the role of vibrationally excited states of CO_2_ are less significant and/or where heating dominates the chemistry.

Although in non‐thermal plasmas, CO_2_ conversion is initiated by electron impact dissociation reactions–which can be enhanced by increasing specific energy input–the observed decline in CO_2_ conversion with increased gas temperature can be attributed to thermally‐driven chemistry. The positive correlation between the CO_2_ conversion and O_3_ production reveals how the competition for O radicals between (i) the reactions producing O_2_/O_3_ (R10, R11, and R13) and (ii) the recombination reaction of CO/CO(a ^3^Π) with O to CO_2_ (R6/R7) determines the CO_2_ conversion as a function of the gas temperature. This highlights the importance of considering O_3_ reactions in plasma‐based CO_2_ conversion research.

Lastly, plasmas with high gas temperatures, such as warm plasmas, provide more favorable conditions for achieving high CO_2_ conversion and energy efficiency compared to low‐temperature plasmas, such as room‐temperature plasmas. This is because at high temperatures (*T*_g_>2500 K), plasma‐based CO_2_ conversion is governed by thermally‐driven reactions, which have lower activation energies for CO_2_ dissociation compared to electron‐driven reactions. Future work should focus on (i) maximizing the electrical energy transfer for homogenous heating of the gas to *T*_g_=3600 K for maximum efficiency and (ii) utilizing techniques to limit the back production of CO_2_, such as fast quenching, O_2_‐removal with C, or removing CO with membranes. Although scavenging of O radicals could also benefit plasmas with low gas temperatures, it becomes clear that the gain in conversion will be limited and, thus, the energy efficiency is unlikely to become competitive with warmer plasmas for pure CO_2_ conversion.

## Methods

### Experimental Details

**Figure 8:**
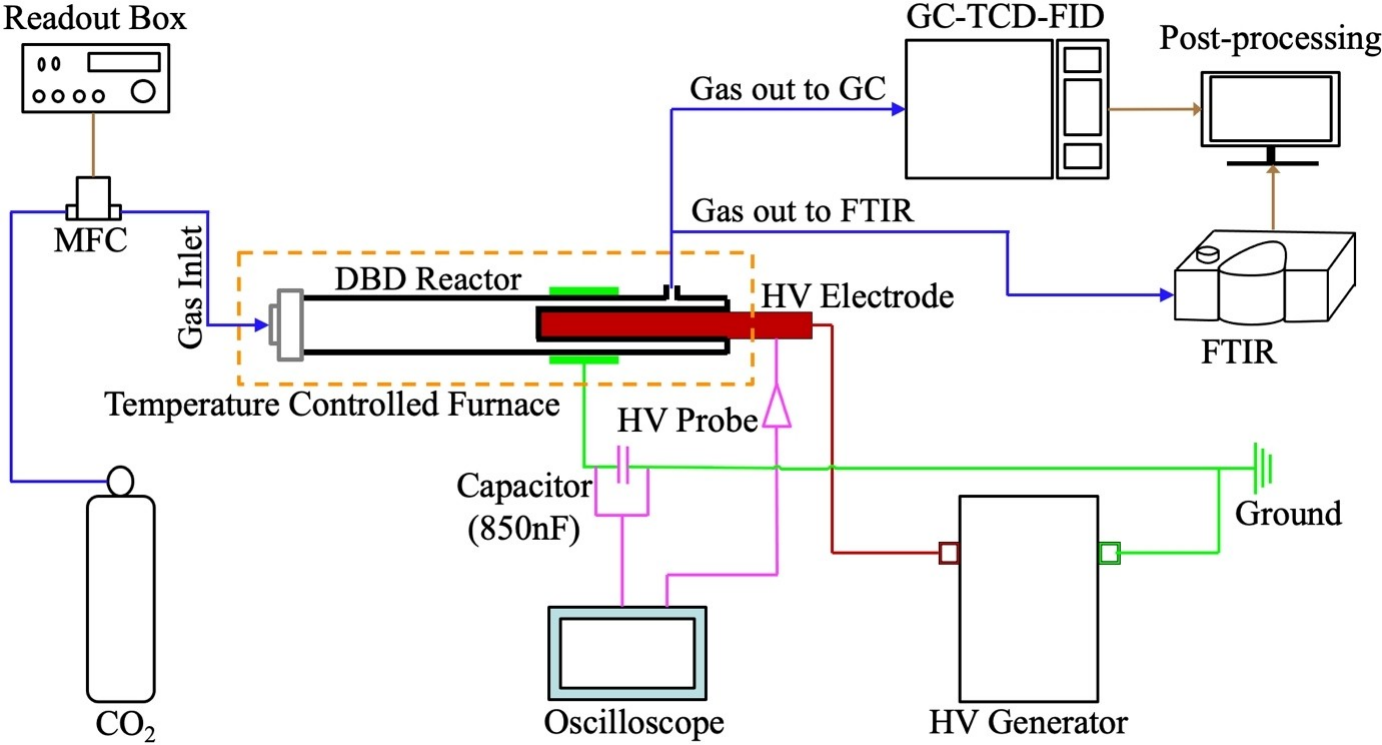
Experimental setup for the temperature‐controlled dielectric barrier discharge (DBD) experiments.

The setup included a temperature‐controlled Dielectric Barrier Discharge (DBD) reactor, a gas feed system, power supply, and an analysis unit (Figure 8). Mass flow controllers (MKS SLS5850) regulated the flow of grade 5 (99.999 %) CO_2_ and N_2_. A total CO_2_ flow rate of 200 SCCM was fed to the reactor, while N_2_ was bypassed and used as external reference for the GC measurements.

The reactor featured a double‐walled DBD design with a 2 mm discharge gap, housed in a furnace capable of reaching 1473 K. The high‐voltage (HV) electrode, made of stainless steel, was placed inside an inner quartz tube, while the ground electrode surrounded the outer quartz tube. The discharge volume was 13.07 cm^3^, with a residence time of approximately 3.57 s at *T*_g_=300 K. The reactor comprised two zones: a preheating zone for gas thermalization and a plasma discharge zone. CO_2_, CO, and N_2_ concentrations were determined using an Agilent 7890 A GC with FID and TCD detectors, while O_3_ concentrations were determined using FTIR (Thermo Fischer Scientific, Nicolet iS10) with a 2 m gas cell. A Lissajous curve (Q−V plot) was used to determine specific energy input (SEI) (from the calculated discharge power (*P*_dis_)), and the reduced electric field (*E*/*N)*.

The experimental procedure involved stabilizing the gas temperature (*T*_g_) for 30 minutes before collecting data for the pure thermal case (without discharge). The discharge was then activated at SEI=0.35 eV/molecule (*P*_dis_=5 W), stabilized for 27 minutes, and five consecutive measurements of product concentrations were recorded. This process was repeated for higher specific energy inputs (SEI=0.70 and 1.39 eV/molecule, corresponding to *P*_dis_=10 and 20 W) by adjusting the applied AC voltage. All data were normalized against the pure thermal case to account for gas heating effects outside the discharge zone. The sequence was repeated for different gas temperatures.

Further details of the experimental setup and reactor schematics can be found in the more comprehensive Methods section in the SI.

### Modeling Details

Critical parameters, such as reduced electric field, gas temperature, specific energy input and product composition were used to validate the model. The numerical simulations were performed using the 0D plasma‐chemical kinetic platform KAUSTKin, which integrates ZDPlasKin^[55]^ and ChemKin.^[56]^ ZDPlasKin handled electron‐induced reactions with rate constants calculated using Bolsig+, while ChemKin computed thermal reaction rates. The platform iteratively solved species and energy equations until the experimental conditions for specific energy input and residence time were reached, simulating both electron and thermal‐driven reactions (Figure S7).

The micro‐discharge volume in DBDs typically represents 0.1–10 % of the total plasma reactor volume[^20^, ^53^, ^55^, ^56^, ^57^, ^58^, ^59^, ^60^, ^61^] which is occupied by both temporally and spatially distributed filaments. To replicate this filamentary behaviour, periodic rectangular micro‐discharge pulses were applied, with experimentally derived *E*/*N* values. This approach has previously undergone validation across a range of DBD reactors, gas mixtures, and numerical codes.[^15^, ^20^, ^24^, ^31^]

Recent advances in plasma modelling have seen significant contributions from various studies. Notable developments include those of Aerts et al.^[62]^ and Vermeiren et al.,^[63]^ extensive CO_2_ models developed by Kozák et al.^[64]^ and Koelman et al.^[65]^ which accounted for various vibrational levels of CO_2_ and the reduced mechanism by Berthelot et al.,^[66]^ which lumped vibrational excited states of CO_2_ into smaller groups. Integrating the electron Boltzmann equation with state‐to‐state vibrational and electronic kinetics has also been crucial,[^67^, ^68^] emphasizing the role of superelastic collisions on electron energy distribution and reaction rates. Additionally, the vibrational kinetics of CO₂ dissociation highlights the involvement of temperature and vibrational relaxation rates.[^69^, ^70^, ^71^]

The developed mechanism utilizes cross‐sectional data from the IST‐Lisbon database,^[72]^ supplemented with data from Polak and Slovetsky^[33]^ for electronic excitation leading to CO_2_ dissociation. Specifically, dissociation with a threshold of 7 eV produces ground state CO, while a threshold of 11.9 eV generates the metastable CO(a ^3^Π) state, which is crucial for CO_2_ conversion.[^69^, ^73^] For CO ionization reactions, we incorporated data from Straub et al.,^[51]^ and for O_2_ electron‐impact chemistry, we referenced Bang et al.^[31]^ The ionization cross‐sections for CO and O_2_ were adjusted using data from Mangan et al.^[52]^ and Straub et al.,^[53]^ respectively. We adjusted the back reaction CO(a ^3^Π)+O→CO_2_ to match the reaction rate of the ground state CO back reaction.

The thermal mechanism developed includes ten fundamental reactions, three of which involve O_2_ species, namely O+O+M↔O_2_+M, O_3_+O↔O_2_+O_2_ and O_2_+O+M↔O_3_+M. The reaction rates for O+O+M↔O_2_+M were obtained from the work of Tsang and Hampson^[74]^ while the rates for the other two reactions were sourced from Bang et al.^[31]^ To accurately predict O_3_ production, we fine‐tuned the third‐body efficiency for O_2_+O+M↔O_3_+M through iterative adjustments, to CO_2_=0.5 and CO=0.25, thus improving alignment with experimental O_3_ production data and CO_2_ conversion predictions.

Reaction rate coefficients for C‐containing species, including CO+O_2_↔O+CO_2_, C+O_2_↔O+CO and CO+O+M↔CO_2_+M were obtained from the Foundational Fuel Chemistry Model (FFCM).^[75]^ The production and consumption of C_2_O were based on Cenian et al.^[76]^ Since vibrationally excited states have minimal impact in a DBD plasma, we treated reactions involving these states as loss processes, assuming these excited species quickly revert to their ground states. The final mechanism consisted of 51 species and 258 reactions: 78 electron impact reactions, 90 ion reactions, 70 combined excited and neutral reactions and 10 reversible neutral reactions. Detailed reaction rates are provided in the Supplementary Information (Tables S15 and S16).

## Supporting Information Summary

Comprehensive supplementary material is provided, encompassing detailed experimental setup, experimental data, numerical methodologies, supplemental data employed for chemical analysis, and individual reaction mechanism files. The authors have cited additional references within the Supporting Information.[^77^, ^78^, ^79^, ^80^, ^81^, ^82^, ^83^, ^84^, ^85^, ^86^, ^87^, ^88^, ^89^, ^90^, ^91^, ^92^, ^93^, ^94^, ^95^, ^96^, ^97^, ^98^, ^99^]

## Affiliation Statement

Most of the work was performed at the King Abdullah University of Science and Technology (KAUST) (i.e. Conceptualization and Methodology, Investigation, Formal analysis and Visualization, Writing – original draft preparation, Writing – review and editing, Funding acquisition and Resources), part of the work was performed at Empa, Swiss Federal Laboratories for Materials Science and Technology (i.e. Formal analysis and Visualization, Writing – review and editing).

## Author Contributions

Conceptualization and Methodology: RS; Investigation: AM; Formal analysis and Visualization: AM, RS; Writing–original draft preparation: AM, RS; Writing–review and editing: AM, RS, MSC; Funding acquisition and Resources: MSC.

## Conflict of Interests

The authors declare no conflict of interest.

1

## Supporting information

As a service to our authors and readers, this journal provides supporting information supplied by the authors. Such materials are peer reviewed and may be re‐organized for online delivery, but are not copy‐edited or typeset. Technical support issues arising from supporting information (other than missing files) should be addressed to the authors.

Supporting Information

Supporting Information

Supporting Information

Supporting Information

Supporting Information

Supporting Information

Supporting Information

## Data Availability

The data that support the findings of this study are available in the supplementary material of this article.
