# Supplementary material for: Temperature‐Dependent Kinetics of Plasma‐Based CO2 Conversion: Interplay of Electron‐Driven and Thermal‐Driven Chemistry
Source: ChemSusChem. 2024 Nov 20;18(6):e202401526. doi: 10.1002/cssc.202401526 (PMC11912113; doi:10.1002/cssc.202401526)
Supplement: Supplementary file 5 — Supporting Information [file CSSC-18-e202401526-s003.pdf]

ChemSusChem

Supporting Information

Temperature-Dependent Kinetics of Plasma-Based CO_2 Conversion: Interplay of Electron-Driven and Thermal-Driven Chemistry

Aswath Mohanan, Ramses Snoeckx,* and Min Suk Cha

Supporting Information:

Temperature-Dependent Kinetics of Plasma-Based CO_2 Conversion: Interplay of Electron-Driven and Thermal-Driven Chemistry

Aswath Mohanan[1], Ramses Snoeckx[1,2 *], and Min Suk Cha[1]

[1]*CCRC, Physical Science and Engineering Division (PSE), King Abdullah University of Science and Technology (KAUST), Thuwal 23955, Saudi Arabia*

[2]*Laboratory for Advanced Fibers, Empa, Swiss Federal Laboratories for Materials Science and Technology, Lerchenfeldstrasse 5, 9014 St. Gallen, Switzerland*

*Corresponding author: ramses.snoeckx@empa.ch

1. Methods

1.1. Experimental details

The setup consisted of a temperature-controlled Dielectric Barrier Discharge (DBD) reactor, a gas feed system, a power supply system, and an analyzing unit. The gas feed system consisted of mass flow controllers (MKS SLS5850) to regulate the mass flow rates of grade 5 (99.999 %) carbon dioxide (CO_2) and nitrogen (N_2). The total CO_2 flow rate in the reactor was maintained at 200 SCCM. N_2 was bypassed to the reactor output and used as an external reference for the GC measurements to standardize the volume changes due to dissociation of CO_2. The experimental procedure covered a gas temperature (T_g) range of 300–1050 K with increments of 150 K and three distinct discharge powers, P_{dis} = 5, 10 and 20 W corresponding to specific energy inputs, SEI = 0.35, 0.70 and 1.39 eV/molecule. Information on the full set of conditions used for the experiments can be found in Table S1.

1.1.1. Plasma reactor

The reactor design featured a double-walled DBD with an outer (length: 950 mm, O.D: 60 mm, I.D: 54 mm) and an inner quartz tube (length: 430 mm, O.D: 50, I.D: 46 mm) separated by a 2 mm discharge gap. It was housed within an electrical furnace (MTI OTF-1200X-III-S-NT) capable of reaching temperatures up to 1473 K (1200 °C).The inner quartz tube housed the high-voltage electrode which was constructed from a 1 mm-thick sheet of stainless steel rolled into a cylindrical shape. The ground electrode, encircling the outer quartz tube, consisted of stainless-steel wire mesh and measured 40 mm in length. This gives a discharge volume of 13.07 cm^3, featuring a mean residence time of 1070.941 [s.K] / T_g [K] (≈3.57 s at T_g =

300 K). This double-walled DBD reactor design aimed to minimize catalytic effects of bare metal electrodes.

The reactor comprised of two zones: a preheating zone (length: 520 mm) and a plasma discharge zone (Fig. S1).The preheating zone was designed to allow the inlet gas sufficient time to attain thermal equilibrium with a predetermined temperature. Meanwhile, the discharge zone was positioned in the third section of the furnace to ensure a consistent temperature throughout the experimental process.

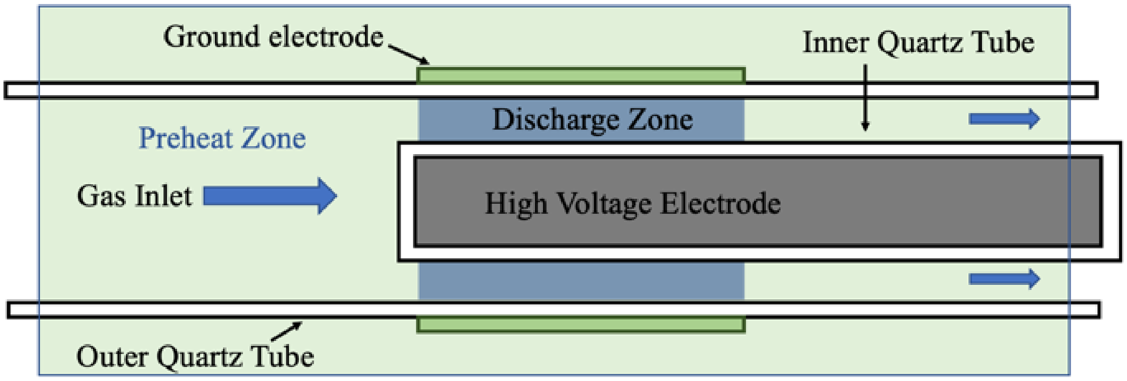

Fig. S1 Schematic of the temperature-controlled dielectric barrier discharge (DBD) reactor.

A function generator (Tektronix AFG 3021B) generated a sinusoidal waveform with an AC frequency of 2.5 kHz, which was amplified to the kV range utilizing a high voltage (HV) power supply (Trek 30/20A). To measure the charge (Q) in the discharge gap, a sampling capacitor (470 nF) was connected in series with the ground electrode. Voltage measurements at the high voltage electrode were taken using a high voltage probe (Tektronix P6015A) and a digital oscilloscope (Tektronix DPO5204B). Simultaneously, the voltage across the sampling capacitor was monitored (Tektronix TPP1000).

1.1.2. Experimental procedure and analysis

The experimental procedure involved several key steps. First, the gas temperature, T_g, was stabilized over a 30-minute period. Data was collected for the pure thermal case (i.e., without discharge) after the temperature was stabilized using an Agilent 7890A Gas Chromatograph (GC) featuring a Flame Ionization Detector (FID) and two Thermal Conductivity Detectors (TCD). Then, the discharge was activated with SEI = 0.35 eV/molecules (P_{dis} = 5 W) and allowed to stabilize for at least 27 minutes. Once the discharge was stable, five consecutive measurements of product concentrations were recorded. This process was then repeated for higher specific energy inputs (SEI = 0.70 and 1.39 eV/molecule) by adjusting the applied AC voltage while maintaining the frequency at 2.5 kHz. The data for each power was normalized using the data obtained from pure thermal case in order to remove the effect of gas heating on species concentration

outside the discharge zone. This entire sequence was repeated to gather data at various gas temperatures. Figure S2 shows the schematic of the experimental setup.

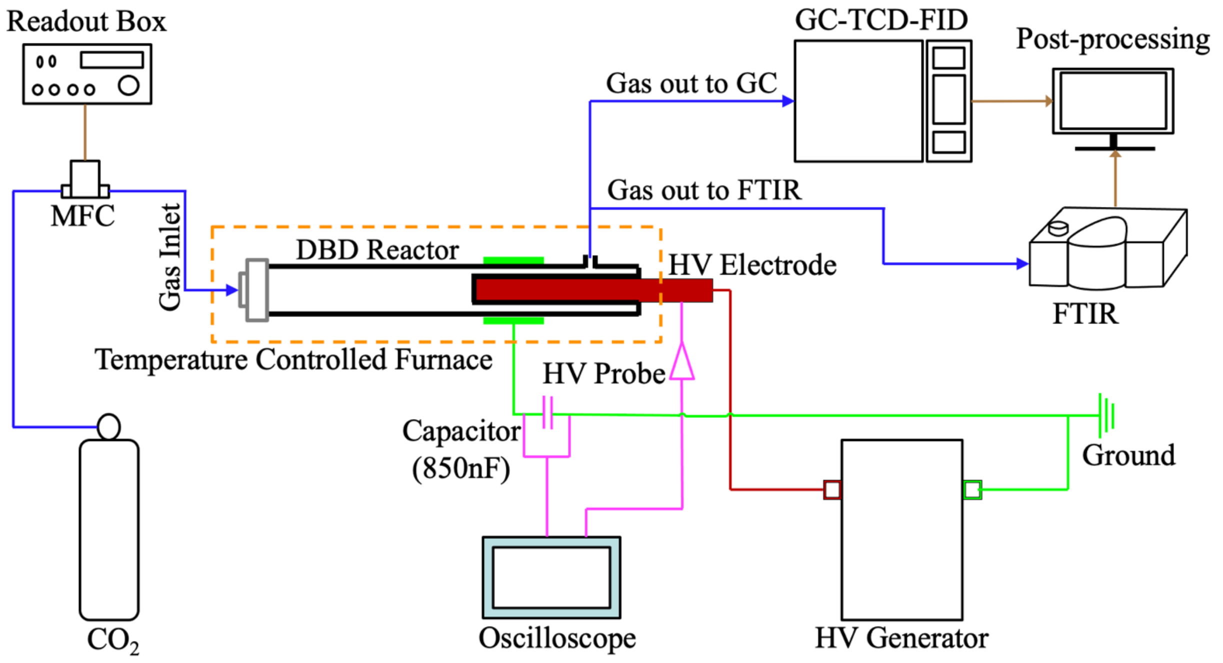

Fig. S2 Schematic of the experimental setup.

A Lissajous curve [1] (*Q*-*V* plot) was constructed by correlating the charge (*Q*) measured from the sampling capacitor with the voltage (*V*) applied at the high voltage electrode. The discharge power (P_{dis}) was calculated based on the area under the Lissajous curve and used to determine the specific energy input based on the fixed flow rate of 200 SCCM. The reduced electric field, gas temperature, product composition, specific energy input and residence time were utilized as input parameters to validate the numerical model.

When the gas flows from the preheating zone to the discharge zone, there is an abrupt change in the cross-section. Hence, the velocity profile in this region would transform into parabolic in nature due to the combined effects of the accelerated flow and boundary wall effect (because the flow was in laminar regime for all tested conditions, Re < 100) as the discharge gap is only 2 mm. However, the micro-discharges along with the ionic winds exert forces in the radial direction of the reactor and could help make the flow more uniform. Since the DBD effects are more pronounced in the region, we assume that the cross-sectional change has minimal effect on the 0D approximation.

To determine O_3 concentrations, we obtained five Fourier Transform InfraRed (FTIR, Thermo Fischer Scientific, Nicolet iS10) measurements with a 2 m gas cell after stabilizing the plasma for 10 minutes. We conducted a calibration of the FTIR using an O_3 monitor (EG-3000, Ebara Jitsugyo Measuring Instrument) using a flow rate of 1.5 SLPM. The calibration curve exhibited a clear linear relationship between the

concentration and the absorption band area with a 337-ppm offset. Therefore, we assigned an uncertainty range of ± 337 ppm to the measurements (see Figure S3).

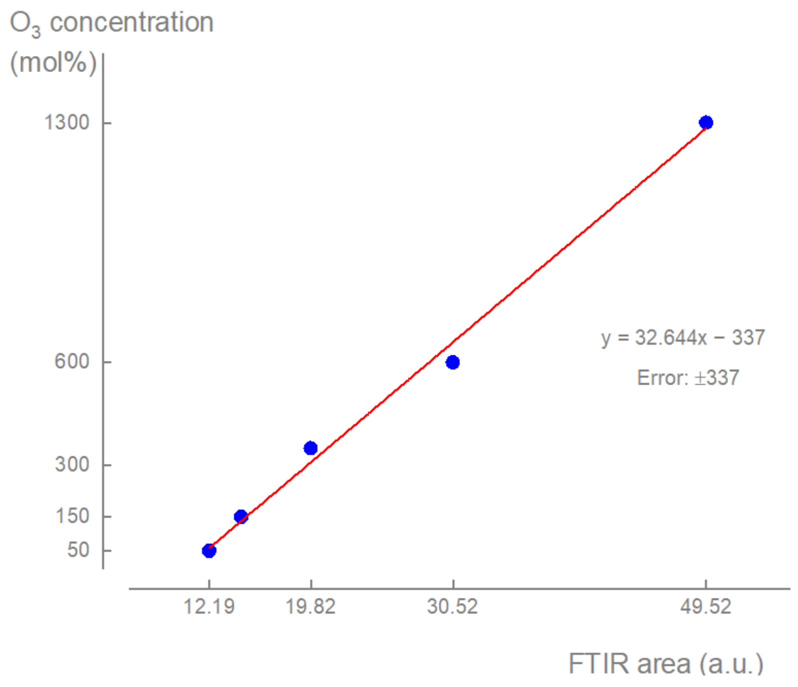

Fig. S3 Calibration curve for the O_3 concentration as function of the FTIR area.

1.1.3. Electrical data

Voltage-current (*V-I*) waveforms for different conditions are plotted in Figure S4. The distortion of the ideal sinusoidal waveform, originally generated by the function generator, became noticeable at higher applied voltages due to the limitations of the HV amplifier (slew rate). This distortion occurred during conditions of elevated specific energy inputs (SEI = 0.70 and 1.39 eV/molecule) and lower gas temperatures (T_g = 300 K). From Figure S4, it is noticable that with increase in gas temperature the peak voltage of the sinusoidal wave decreases and higher specific energy inputs generate higher currents.

In non-thermal plasmas, a significant portion of the electrical energy is channeled into electrons. This is primarily due to the influence of the applied electric field (*E*) and the ensuing Lorentz force. The electrons traverse the electric field, imparting their energy to other particles through collisions. The mean free path between these collisions is inherently related to the number density (*N*). Consequently, the parameter known as the reduced electric field (*E*/*N*) plays a pivotal role in shaping the Electron Energy Distribution Function (EEDF) and determining the mean electron energy (T_e). Hence, the reduced electric field influences both the physical and chemical aspects of non-thermal plasmas.

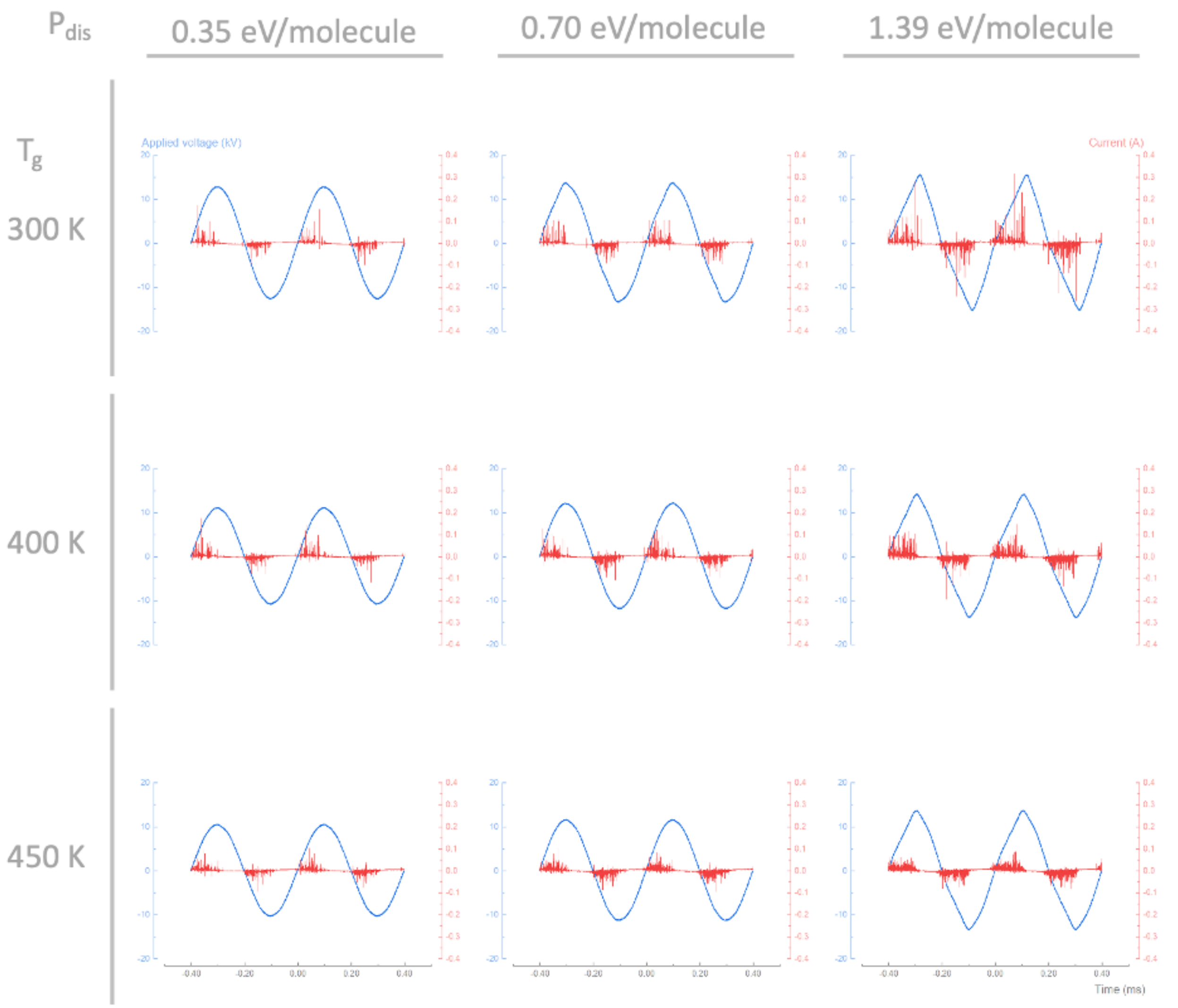

Fig. S4 V-I waveforms for T_g = 300, 400, and 450 K (top to bottom) and SEI = 0.35, 0.70 and 1.39 eV/molecule (left to right). The flow rate was fixed at 200 SCCM and the specific energy input was altered using the input AC voltage with a fixed frequency of 2.5 kHz.

In practice, establishing the precise value of E/N for electrical discharges is challenging. However, in the case of a DBD with a specific gas gap distance (d), it becomes feasible to derive a breakdown voltage (V_B) from a QV-plot (as illustrated in figure S5). This, in turn, enables the estimation of the average electric field ($E \approx V_B/d$) within the DBD reactor [2–4]. Ultimately, by obtaining N using the ideal gas law based on pressure and temperature, it is possible to calculate E/N, as summarized in Table S6. The trend of the obtained E/N value for SEI = 1.39 eV/molecule is shown in figure S6 and is used as an input for the 0D simulations.

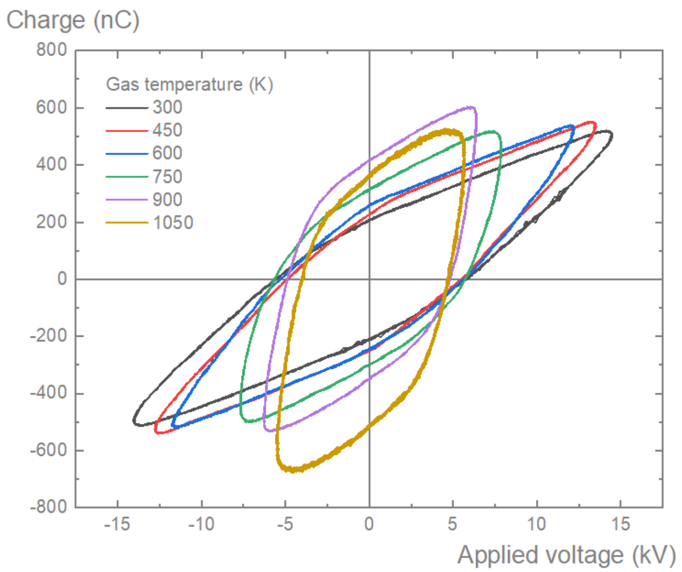

Fig. S5 Lissajous curve (Q-V plot) for different temperatures for SEI = 1.39 eV/molecule and flow rate of 200 SCCM. The applied AC voltage was changed to adjust the specific energy input while the AC frequency was held constant at 2.5 kHz.

We observed that the E/N ratio increased with rising gas temperature (T_g), reaching a peak at 900 K, largely due to the reduction in the number density (N). However, at 1050 K, the E/N ratio began to decline despite the continued decrease in N. This decline was primarily attributed to a substantial drop in the breakdown voltage (V_B) and a slower rate of decrease in N.

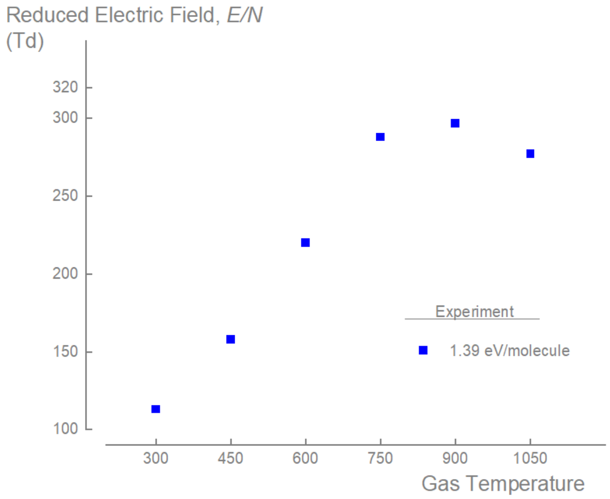

Fig. S6 Calculated reduced electric field (E/N) from experiments conducted in the DBD for T_g = 300 to 1050 K and SEI = 1.39 eV/molecule. Reduced electric field increases with increase in T_g until 900 K and drops when T_g is 1050 K.

1.1.4. Heating effects

For temperatures in the range of 300 to 600 K, the temperature increase due to the discharge was measured 3 cm downstream of the discharge zone. The maximum ΔT_g was found to be 22.9 K at 460 K for SEI = 1.39 eV/molecule (Table S7). A similar observation was found by Bang et al. [5] where only minimal increases in gas temperature were found for similar specific energy inputs. In our study, we noticed that for SEI = 1.39 eV/molecule, ΔT_g reduced between 300 and 350, then increased until 460 K, and finally slowly decreased as the temperature increased. The changes in ΔT_g 300 and 350 K can be attributed to the difference of endothermic reactions (dissociation of CO_2 and O_3) and the exothermic reactions (production of O_3 and CO_2).

1.2. Modeling details

To perform the numerical simulations, we employed the 0D plasma-chemical kinetic modeling platform KAUSTKin [6]. This platform seamlessly integrates two fundamental solvers, namely ZDPlasKin [7] and ChemKin [8], serving as an intermediary for sequential resolution of plasma reactions and thermal chemistry.

1.2.1. 0D chemical kinetics model

KAUSTKin is designed to solve the time-dependent species and energy equations, operating in a zero-dimensional (0D) framework. It initially solves the plasma energy equations using the ZDPlasKin solver relying on the Bolsig+ solver [9] for rate constants calculations for electron-induced reactions while considering collision cross-sections and the corresponding electron energy distribution function (EEDF). Meanwhile, conventional thermal reaction rates are determined using the Chemkin library. The schematic working of KAUSTKin is shown in figure S7.

ZDPlasKin calculates the time evolution of species densities during a timestep, considering the defined production and consumption processes of plasma chemical reactions. Subsequently, KAUSTKin retrieves the solution from ZDPlasKin and employs it as an initial condition for Chemkin. Thereafter, the time evolution of all species densities over the same timestep based on classical thermal chemical reactions is calculated. The results are once again collected by KAUSTKin and transferred to ZDPlasKin for the next iteration. This iterative process continues until the experimentally determined duration of time (residence time) and specific input energy is reached. This approach facilitates the integration of two distinct types of reactions observed in plasma discharges: those influenced by the electron temperature and those regulated by the gas temperature, all the while ensuring the numerical stability of the individual solvers.

To emulate the typical filamentary behavior characteristic of DBDs (revealed by the *V-I* waveform in figure S4), where gas molecules traverse multiple micro-discharge filaments during their passage, we implemented numerous consecutive micro-discharge pulses with a uniform electric field in time. This approach has previously undergone validation across a range of DBD reactors, gas mixtures, and numerical codes [5,10–12].

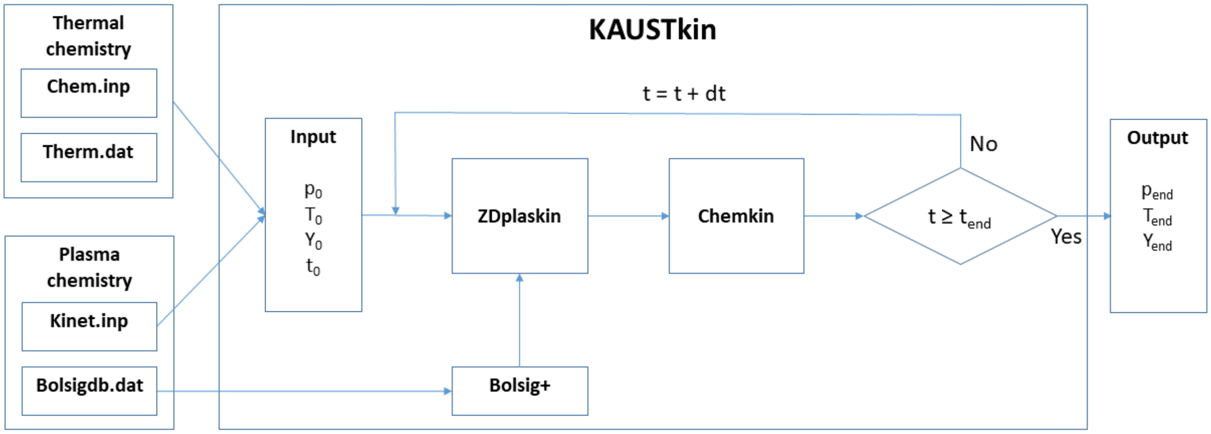

Fig. S7 Schematic of the modules and data flow of the plasma-chemical kinetic model, KAUSTKin. Reprinted from Combustion and Flame, 242, R. Snoeckx, D. Jun, B.J. Lee, and M.S. Cha, Kinetic study of plasma assisted oxidation of H2 for an undiluted lean mixture, 112205, Copyright (2022), with permission from Elsevier.

1.2.2. Modeling the micro-discharges in the DBD

In a DBD, the total micro-discharge volume is significantly smaller than the total volume of the plasma reactor, typically on the order of 0.1 to 10 % [10, 13–14]. Consequently, both temporally and spatially distributed filaments exist, occupying only a minor fraction of the overall discharge volume and residence time. To replicate the filamentary behavior in our modeling approach, we applied periodic rectangular micro-discharge pulses. These pulses had durations ranging from 5 to 100 ns, contingent upon the gas temperature. Experimental values of *E*/*N* were obtained as described in section 1.1.3 and Table S6. In reality, *E*/*N* exhibits a combined space-time evolution for each individual filament. However, providing an accurate description of each filament would necessitate multidimensional modeling, exceeding the scope of our current work.

For the conditions investigated, we applied a total of 288 micro-discharges, an artificial value, as the exact number of micro-discharge filaments that gas molecules encounter when passing through the reactor is unknown. Generally, it has been observed that employing a substantial number of consecutive micro-discharge pulses in the hundreds results in satisfactory agreement with experimental data concerning overall

species density changes [5,10–12]. Further increasing the number of pulses had a minimal effect on overall species density changes but significantly escalated computational costs. Decreasing the number of discharges had a negative impact on the outcome as well as the stability of the solver. The critical parameters that determine model outcomes, and hence need to align with experimental data, include the specific energy input (SEI) or energy density (ED) and the strength of the reduced electric field (E/N).

The energy deposited in the model (SEI = 0.35, 0.70, and 1.39 eV/molecule for P_{dis} = 5, 10 and 20 W respectively) was achieved by maintaining a fixed energy per pulse, determined by dividing the SEI by the total number of pulses employed in the model.

1.2.3. Plasma-chemical reaction mechanism

Special attention was paid in compiling cross-section data for key electron impact reactions, namely dissociation and ionization, as identified in the literature [15,16]. For the CO_2 mechanism, we primarily referenced electron impact reactions for CO_2 and CO species from the IST-Lisbon [17] database as reported in LXCAT.

The 7 eV and 10.5 eV excitations of CO_2 listed in the IST-Lisbon database is available from the work of Lowke et al. [18] and also available in the Phelps database [19] extended to 1000 eV. The 7 eV excitation was considered to be dissociative and 10.5 eV excitation as electronic excitation (loss process) in the initial version of our mechanism. To refine ionization reactions of CO_2, we incorporated data mentioned in the work of Straub et al. [20]. The electron-impact chemistry of O_2 was obtained from the work of Bang et al. [9]. Furthermore, we adjusted the ionization cross-sections of CO and O_2 using data available in the works of Mangan et al. [21] and Straub et al. [22] respectively. This mechanism from here forth will be referred as “Pure IST” mechanism.

The "Pure-IST" mechanism led to an overestimation, particularly at low E/N values. To enhance the accuracy of the results, we made modifications to the dissociation cross-section at 7 eV by incorporating cross-sections calculated by Polak and Slovetsky [23]. We replaced the electronic excitation at 10.5 eV with the dissociation of CO_2 generating metastable CO(a $^3\Pi$) having a threshold energy of 11.9 eV. Furthermore, we took into account the electronic excitation of CO to CO(a $^3\Pi$) for which cross-sectional data was available in the IST-Lisbon database. The back reaction CO(a $^3\Pi$) + O leading to the production of CO_2 was modified to have the same reaction rate as that of its ground state.

Henceforth, this modified mechanism, which incorporates the formation of CO(a $^3\Pi$), is referred to as "IST-Polak with CO(a $^3\Pi$)" and represents the final mechanism used in our study as presented in the main manuscript. Detailed comparisons between the two mechanisms are provided in section 1.2.4.

The thermal mechanism we developed consisted of ten fundamental reactions. Among these, three reactions were associated with O_2 species, namely $O+O+M \leftrightarrow O_2+M$, $O_3+O \leftrightarrow O_2+O_2$ and $O_2+O+M \leftrightarrow O_3+M$. The reaction rate of $O+O+M \leftrightarrow O_2+M$ was obtained from the work of Tsang and Hampson [24]. The reaction rates of the other two reactions were sourced from the work of Bang et al. [9]. To ensure accurate prediction of O_3 production, we fine-tuned the third body efficiency of $O_2+O+M \leftrightarrow O_3+M$ through iterative adjustments, arriving at the values of CO_2 = 0.5 and CO = 0.25. As a result, the revised mechanism demonstrated agreement with experimental O_3 production data while improving the prediction of CO_2 conversion. The reaction rate coefficients for the reactions involving C-containing species, $CO+O_2 \leftrightarrow O+CO_2$, $C+O_2 \leftrightarrow O+CO$ and $CO+O+M \leftrightarrow CO_2+M$ were obtained from the Foundational Fuel Chemistry Model (FFCM) [25]. The production and consumption of C_2O was taken from the work of Cenian et al. [26]. The list of the main species used in the mechanism is given in Table S8. Since for a DBD, the vibrational excited states do not play an important role, the reactions involving the production of these species have been considered as a loss process i.e., the assumption that these excited species instantaneously revert back to their ground states. The developed mechanism consisted of 51 species and 258 reactions: 78 electron impact reactions, 90 ion reactions, 70 combined excited and neutral reactions and 10 reversible neutral reactions. The complete set of reactions and their rates is available in section 5.2.

1.2.4. Comparison between "Pure-IST" and "IST-Polak with CO(a $^3\Pi$)"

As mentioned in section 1.2.3, the only difference between the "Pure IST" and "IST-Polak with CO(a $^3\Pi$)" mechanisms lies in the cross-sections of dissociation at 7eV and the replacement of the loss process at 10.5 eV with dissociation leading to the formation of CO(a $^3\Pi$). As can be seen from the figure S8, the cross-section of Phelps used in "Pure IST" are a magnitude higher than that of Polak and Slovetsky.

This explains the overprediction of conversion throughout the temperature range especially at lower T_g by "Pure IST" as seen in figure S9. In order to have better results in the lower T_g regime and the simulation as whole, we developed the "IST-Polak with CO(a $^3\Pi$)" mechanism, which closely agreed with experimental values.

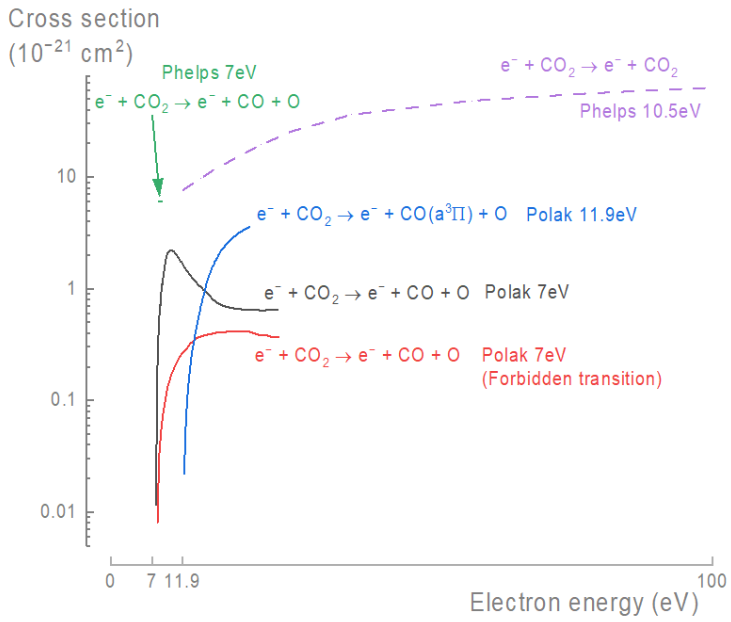

Fig. S8 Cross-section used for the dissociation of CO_2. Phelps cross-sections given in the IST database [19] are used in the "Pure IST" mechanism and the cross-sections suggested by Polak and Slovetsky [23] are used in the "IST-Polak with CO(a $^3\Pi$)" mechanism.

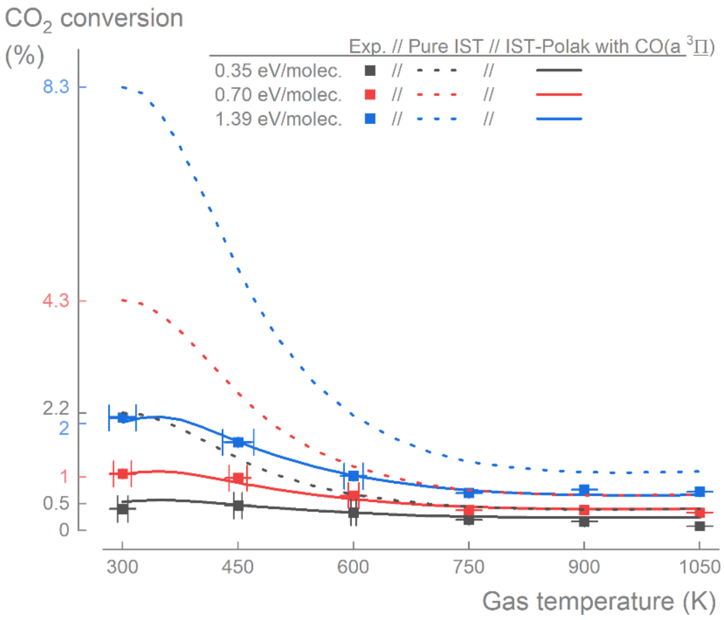

Fig. S9 Comparison of CO_2 conversion for "Pure IST" (- -) and "IST-Polak with CO(a $^3\Pi$)" (—) mechanisms. The experimental values are given as symbols for comparing the accuracy of the mechanisms. The "Pure IST" mechanism overpredicts the conversion throughout the temperature range especially at low temperatures. The results of the "IST-Polak with CO(a $^3\Pi$)" align well with the experimental data.

2. Detailed data

2.1. Experimental data

The conditions used for the experiments are given in the table below.

Table S1 Experimental conditions used.

Discharge Power (P_{dis}) [W]<br>Specific Energy Input (SEI) [eV/molecule]	5 / 10 / 20<br>0.35 / 0.70 / 1.39
Gas Flow Rate [SCCM]	200
Gas Temperature (T_g) [K]	300–1050 (ΔT_g = 150)

2.1.1. CO_2 conversion data

The following tables give the experimental data for the conversion of CO_2 at different experimental conditions.

Table S2 The measured average plasma discharge power (P_{dis}), specific energy input (SEI) and average conversion of CO_2 (χ_{CO_2}) for SEI = 0.35 eV/molecule**.**

T_g (K)	P_{dis} (W)	SEI (eV/molecule)	χ_{CO_2} (%)
**300**	5.0	0.35	0.4
**450**	5.0	0.35	0.5
**600**	5.1	0.36	0.3
**750**	5.0	0.35	0.2
**900**	5.0	0.35	0.2
**1050**	5.1	0.36	0.1

Table S3 The measured average plasma discharge power (P_{dis}), specific energy input (SEI) and average conversion of CO_2 (χ_{CO_2}) for SEI = 0.70 eV/molecule.

T_g (K)	P_{dis} (W)	SEI (eV/molecule)	χ_{CO_2} (%)
**300**	10.1	0.71	1.1
**450**	10.1	0.71	1.0
**600**	10.0	0.70	0.7
**750**	10.1	0.71	0.4
**900**	10.1	0.71	0.4
**1050**	10.0	0.70	0.3

Table S4 The measured average plasma discharge power (P_{dis}), specific energy input (SEI) and average conversion of CO_2 (χ_{CO_2}) for SEI = 1.39 eV/molecule.

T_g (K)	P_{dis} (W)	SEI (eV/molecule)	χ_{CO_2} (%)
**300**	20.2	1.40	2.1
**450**	20.0	1.39	1.7
**600**	20.0	1.39	1.0
**750**	20.0	1.39	0.7
**900**	20.0	1.39	0.8
**1050**	20.1	1.40	0.7

2.1.2. O_3 production data

The following table give the experimental data for the production of O_3 at different experimental conditions.

Table S5 Measured average production of O_3 (χ_{O_3}) for SEI = 0.35, 0.70 and 1.39 eV/molecule for different temperatures.

T_g (K)	χ_{O_3} (ppm)		
	**0.35 eV/molecule**	**0.70 eV/molecule**	**1.39 eV/molecule**
**300**	720.5	994.6	1193.1
**350**	419.2	579.6	649.1
**400**	236.5	266.9	268.3
**450**	16.2	17.2	18.1
**460**	3.9	7.0	11.0
**470**	0.0	0.0	0.0

2.1.3. Electrical data

The measured average peak voltage from the Lissajous curve and the subsequent calculation of breakdown voltage and reduced electric field is given in the table below.

Table S6 Measured average peak voltage (V_{PP}), breakdown voltage (V_B) and reduced electric field (E/N) for SEI = 1.39 eV/molecule.

T_g (K)	1.39 eV/molecule		
	$V_{p\text{-}p}$ (V)	V_B (kV)	E/N (Td)
**300**	14.5	5.5	113.0
**450**	13.4	5.2	157.9
**600**	12.2	5.4	220.2

**750**	7.6	5.6	288.0
**900**	6.3	4.9	297.1
**1050**	5.5	4.5	277.4

2.1.4. Heating data

The increase in temperature of the gas after 30 minutes of plasma discharges is given below.

Table S7 Temperature increase after 30 min of switching on the plasma reactor in pure CO_2.

T_g (K)	ΔT_g (K)		
	SEI = 0.35 eV/molecule	0.70 eV/molecule	1.39 eV/molecule
**300**	6.7	11.4	17.5
**350**	7.2	10.7	14.7
**400**	4.6	10.2	18.6
**450**	5.5	11.7	20.3
**460**	5.5	11.9	22.9
**470**	4.0	9.8	21.2
**500**	5.6	10.1	19.4
**550**	5.4	8.0	14.5
**600**	3.2	6.7	12.4

2.2. Modeling data

The list of species included in the compiled reaction mechanism is shown in table S8.

Table S8 Species included in the reaction mechanism.

	Neutral	Main Excited Species	Charged
electrons			e^-
C-containing	C<br>CO<br>CO_2<br>C_2O	CO(a $^3\Pi$)	C^+<br>CO^+<br>CO_2^+
O-containing	O<br>O_2<br>O_3	O(^{1}D), O(^{1}S)<br>O_2(a $^1\Delta_g$), O_2(b $^1\Sigma_g^+$), O_2(c = A $^3\Sigma_u^+$ + A' $^3\Delta_u$ + c $^1\Sigma_u^-$)	O^+, O^-<br>O_2^+, O_2^-<br>O_3^-<br>O_4^+, O_4^-

2.2.1. Modelling input data

Based on the experimental conditions and data, the following were taken as input for the numerical model: Gas temperature (T_g), reduced electric field (E/N) and the residence time calculated from the flow rate and volume of the reactor. The details of the input parameters to the model is shown in table S9 below.

Table S9 Input parameters for the different modeling conditions.

T_g (K)	E/N (Td)	Residence time (s)		# Discharge pulses	Inter-pulse time (ms)
		Discharge zone	Total		
**300**	113.0	3.57	3.68	288	12.4
**325**	120.5	3.30	3.40	288	11.4
**350**	128.0	3.06	3.16	288	10.6
**375**	135.5	2.86	2.95	288	9.9
**400**	142.9	2.68	2.76	288	9.3
**450**	157.9	2.38	2.45	288	8.3
**460**	162.1	2.33	2.40	288	8.1
**470**	166.2	2.28	2.35	288	7.9
**500**	178.7	2.14	2.21	288	7.4
**550**	199.4	1.95	2.01	288	6.8
**600**	220.2	1.78	1.84	288	6.2
**650**	242.8	1.65	1.70	288	5.7
**700**	265.4	1.53	1.58	288	5.3
**750**	288.0	1.43	1.47	288	5.0
**800**	291.1	1.34	1.38	288	4.7
**850**	294.1	1.26	1.30	288	4.4
**900**	297.1	1.19	1.23	288	4.1
**950**	290.5	1.13	1.16	288	3.9
**1000**	284.0	1.07	1.10	288	3.7
**1050**	277.4	1.02	1.05	288	3.5

2.2.2. Model conversion data

Table S10 Calculated CO_2 conversion (χ_{CO_2}) for different temperatures for SEI = 0.35, 0.70 and 1.39 eV/molecule.

	χ CO_2 (%)				χ CO_2 (%)		
**T_g (K)**	0.35 eV/molecule	0.70 eV/molecule	1.39 eV/molecule	**T_g (K)**	0.35 eV/molecule	0.70 eV/molecule	1.39 eV/molecule
**300**	0.54	1.05	2.03	**600**	0.34	0.59	1.03
**325**	0.56	1.10	2.12	**650**	0.30	0.52	0.91
**350**	0.57	1.11	2.13	**700**	0.28	0.48	0.82
**375**	0.56	1.09	2.07	**750**	0.26	0.45	0.76
**400**	0.54	1.03	1.96	**800**	0.25	0.43	0.71
**450**	0.48	0.90	1.67	**850**	0.25	0.41	0.68
**460**	0.46	0.86	1.61	**900**	0.24	0.40	0.67
**470**	0.45	0.85	1.56	**950**	0.24	0.40	0.66
**500**	0.42	0.77	1.40	**1000**	0.24	0.40	0.66
**550**	0.37	0.67	1.19	**1050**	0.25	0.41	0.67

2.2.3. Ozone production

Table S11 Calculated O_3 conversion (χ_{O_3}) for different temperatures for SEI = 0.35, 0.70 and 1.39 eV/molecule.

	χ O_3 (ppm)				χ O_3 (ppm)		
**T_g (K)**	0.35 eV/molecule	0.70 eV/molecule	1.39 eV/molecule	**T_g (K)**	0.35 eV/molecule	0.70 eV/molecule	1.39 eV/molecule
**300**	364.25	696.19	1283.81	**600**	0.65	0.75	0.81
**325**	227.39	433.00	796.41	**650**	0.15	0.08	0.04
**350**	132.51	250.83	459.92	**700**	0.05	0.02	0.00
**375**	75.23	141.00	257.04	**750**	0.02	0.01	0.00
**400**	42.83	79.14	143.00	**800**	0.01	0.01	0.00

**450**	14.84	26.36	46.20	**850**	0.01	0.00	0.00
**460**	12.20	21.45	37.28	**900**	0.00	0.00	0.00
**470**	10.06	17.51	30.13	**950**	0.00	0.00	0.00
**500**	5.73	9.60	15.88	**1000**	0.00	0.00	0.00
**550**	2.18	3.31	4.82	**1050**	0.00	0.00	0.00

3. Extended chemical analysis

3.1. Densities, production, and consumption rates of the main species

Figure S10 illustrates the net change in the major species for T_g range of 300–1050 K and SEI = 0.35, 0.70 and 1.39 eV/molecule. The positive values indicate the net production while negative values indicate net consumption of the species. S11–12 depicts the overall production and consumption of these species. These values are derived from the integration of production and consumption rates of relevant reactions throughout the entire simulation duration, which corresponds to the experimental residence time. All three parameters have a unit of number of species per cm^3.

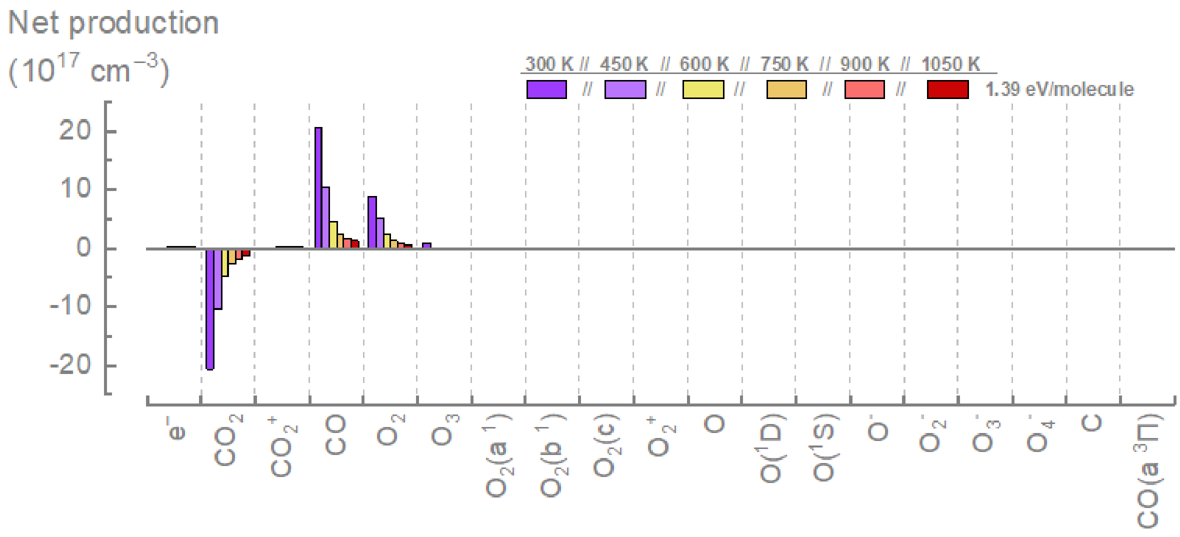

Fig. S10 Net production of the main species for T_g range of 300–1050 K and SEI = 1.39 eV/molecule.

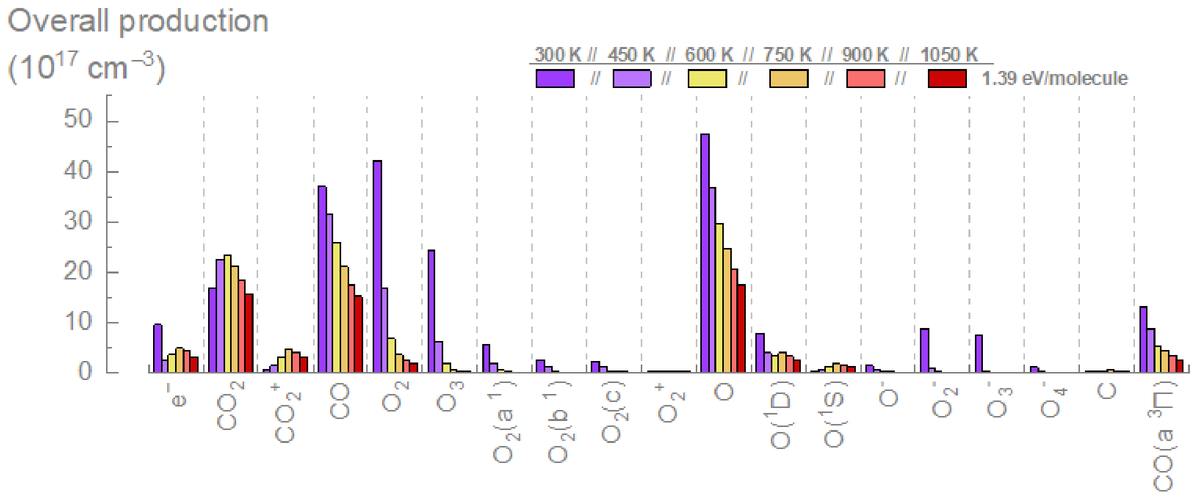

Fig. S11 Overall production of the main species for T_g range of 300–1050 K and SEI = 1.39 eV/molecule.

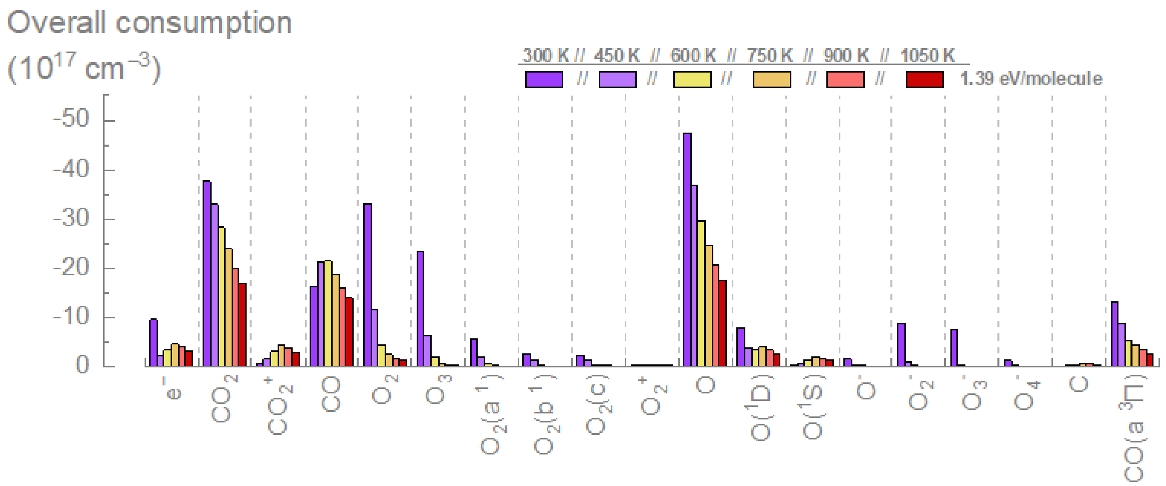

Fig. S12 Overall consumption of the main species for T_g range of 300–1050 K and SEI = 1.39 eV/molecule.

3.2. Reaction rates and contributions of the main elementary reactions

Through the chemical analysis, we identified 101 elementary reactions as significant when examining the primary species and their production and consumption rates. Figure S13 illustrates the cumulative impact of each reaction, determined through the integration of the reaction rate over the complete simulation duration.

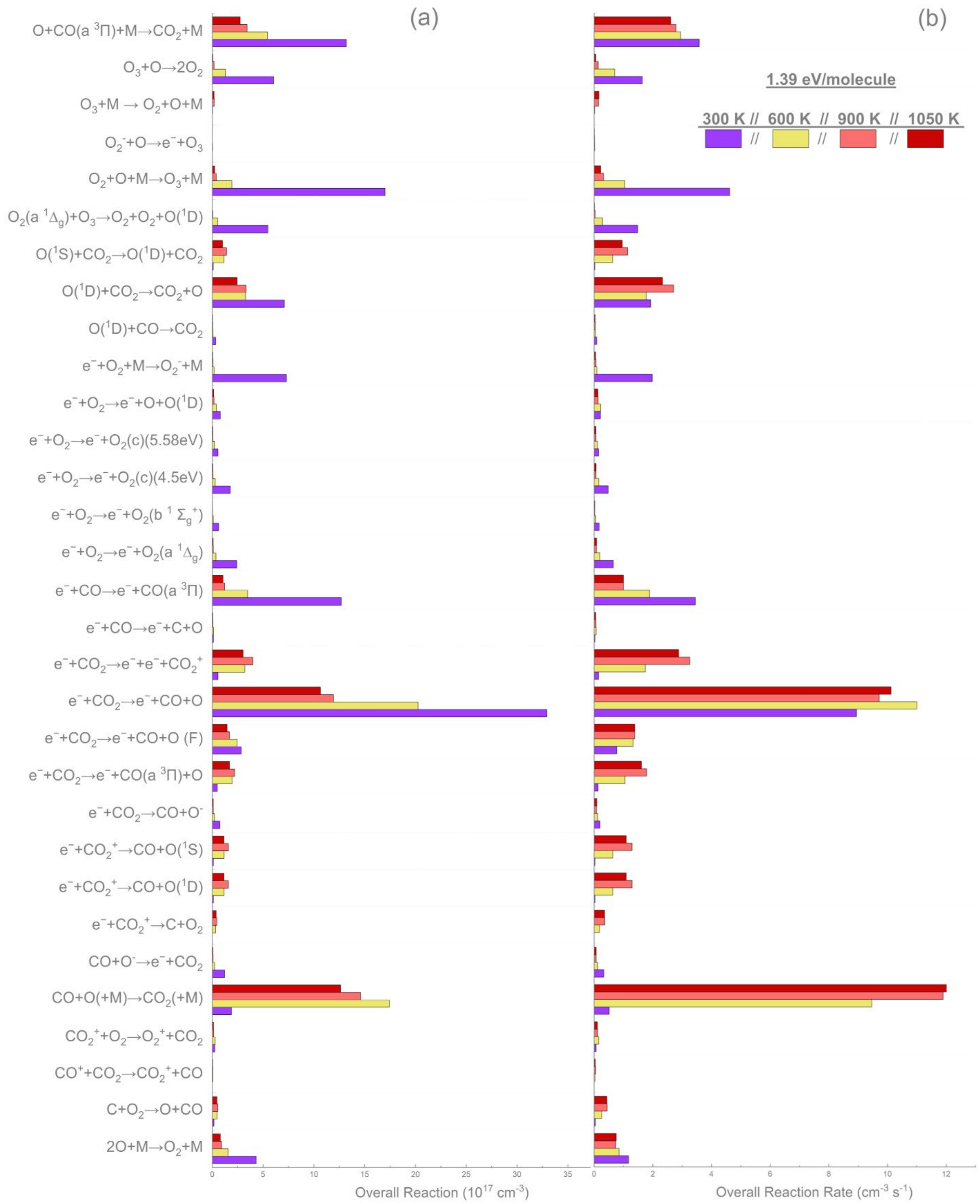

Fig. S13 Contribution of the main elementary reactions: (a) Overall reaction on left (b) Overall reaction rate on right for T_g range of 300–1050 K and SEI = 1.39 eV/molecule.

To further understand the chemical pathways, we also need to look at the contribution of individual elementary reactions in the production and consumption of the major species: CO_2, CO, CO_2^+, CO(a $^3\Pi$), O, O_2 and O_3 (see Figures S14–20).

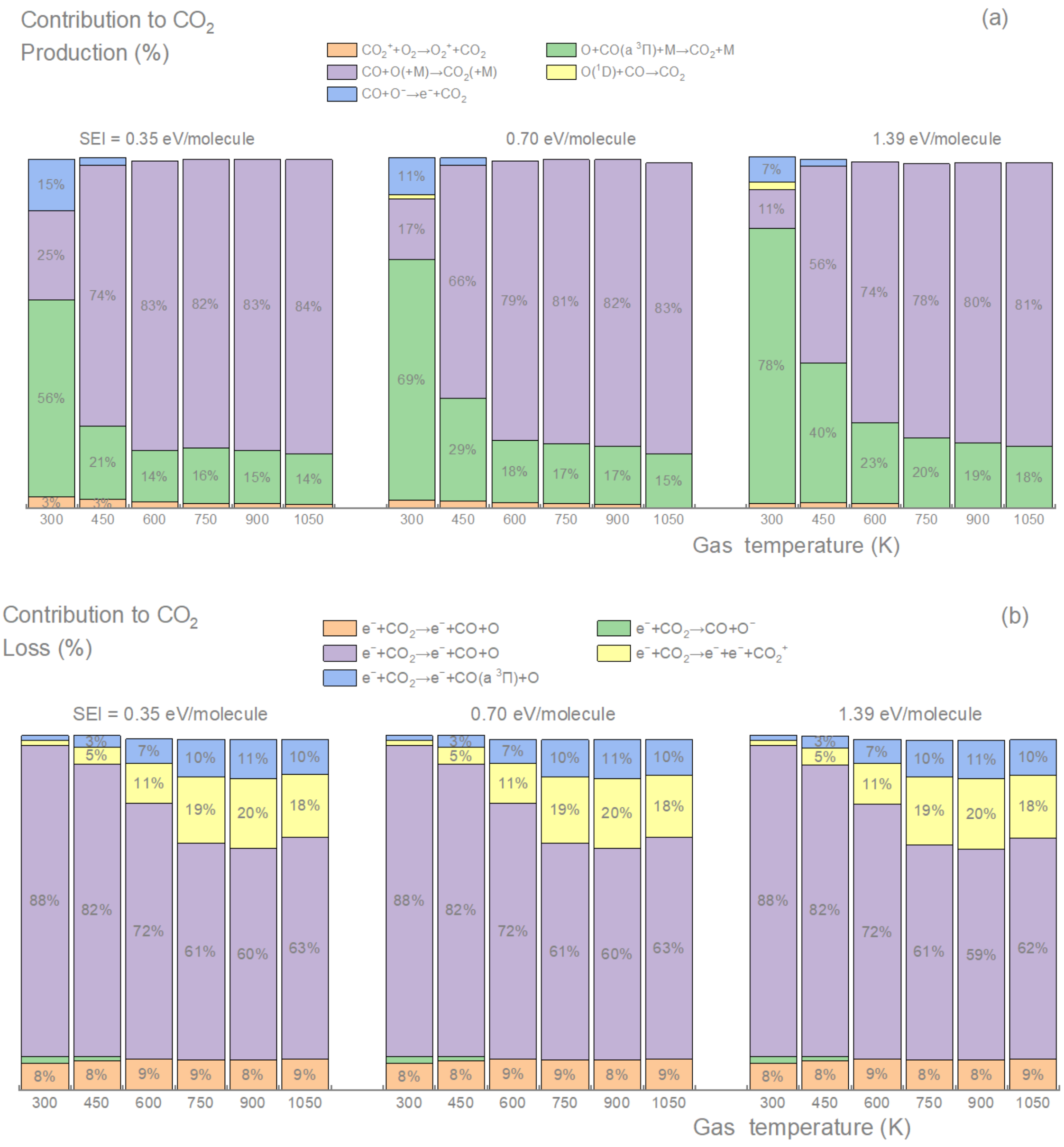

Fig. S14 Contribution of the elementary reactions in (a) production (b) consumption of CO_2 for T_g range of 300–1050 K and SEI = 0.35, 0.70 and 1.39 eV/molecule.

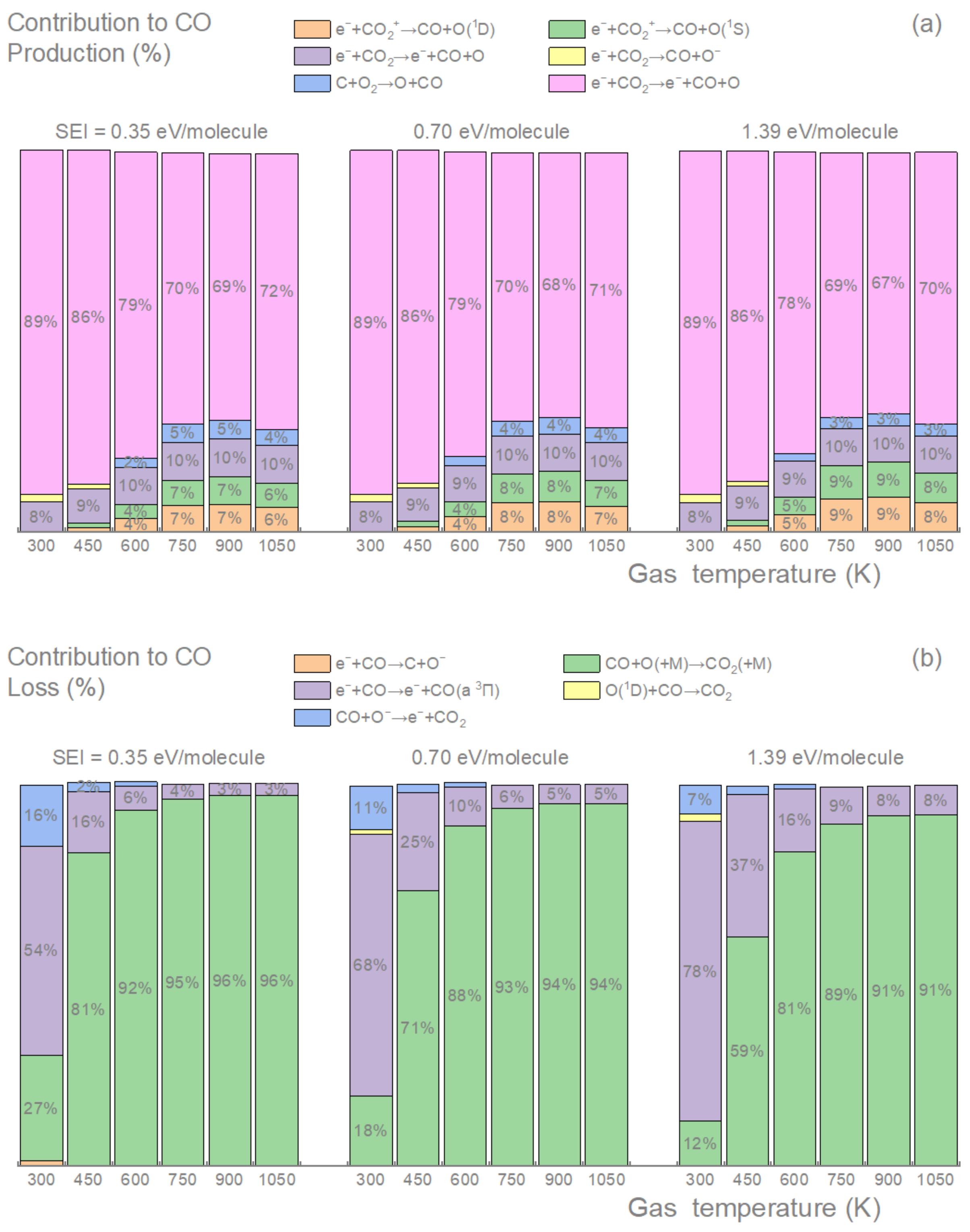

Fig. S15 Contribution of the elementary reactions in (a) production (b) consumption of CO for T_g range of 300–1050 K and SEI = 0.35, 0.70 and 1.39 eV/molecule.

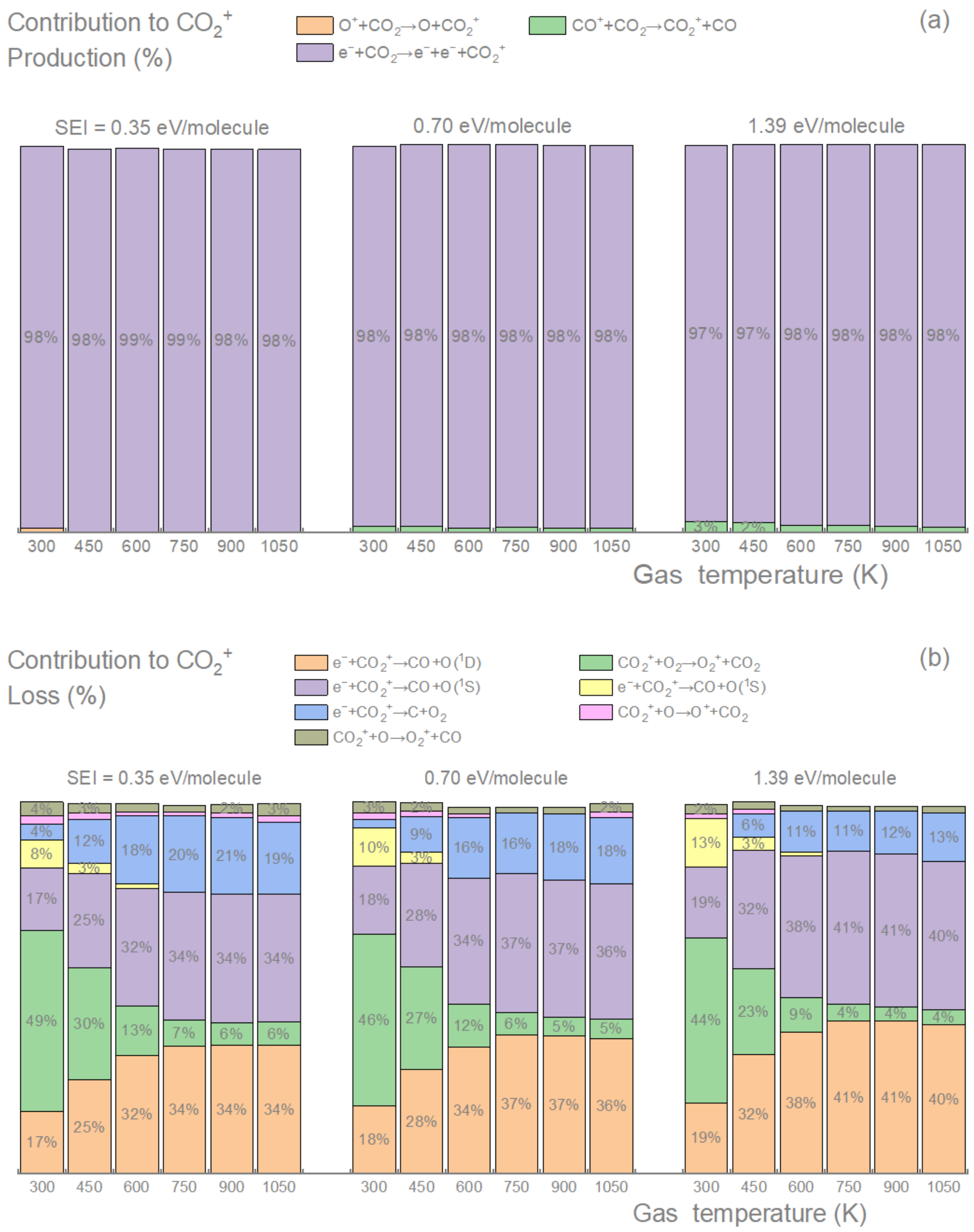

Fig. S16 Contribution of the elementary reactions in (a) production (b) consumption of CO_2^+ for T_g range of 300–1050 K and SEI = 0.35, 0.70 and 1.39 eV/molecule.

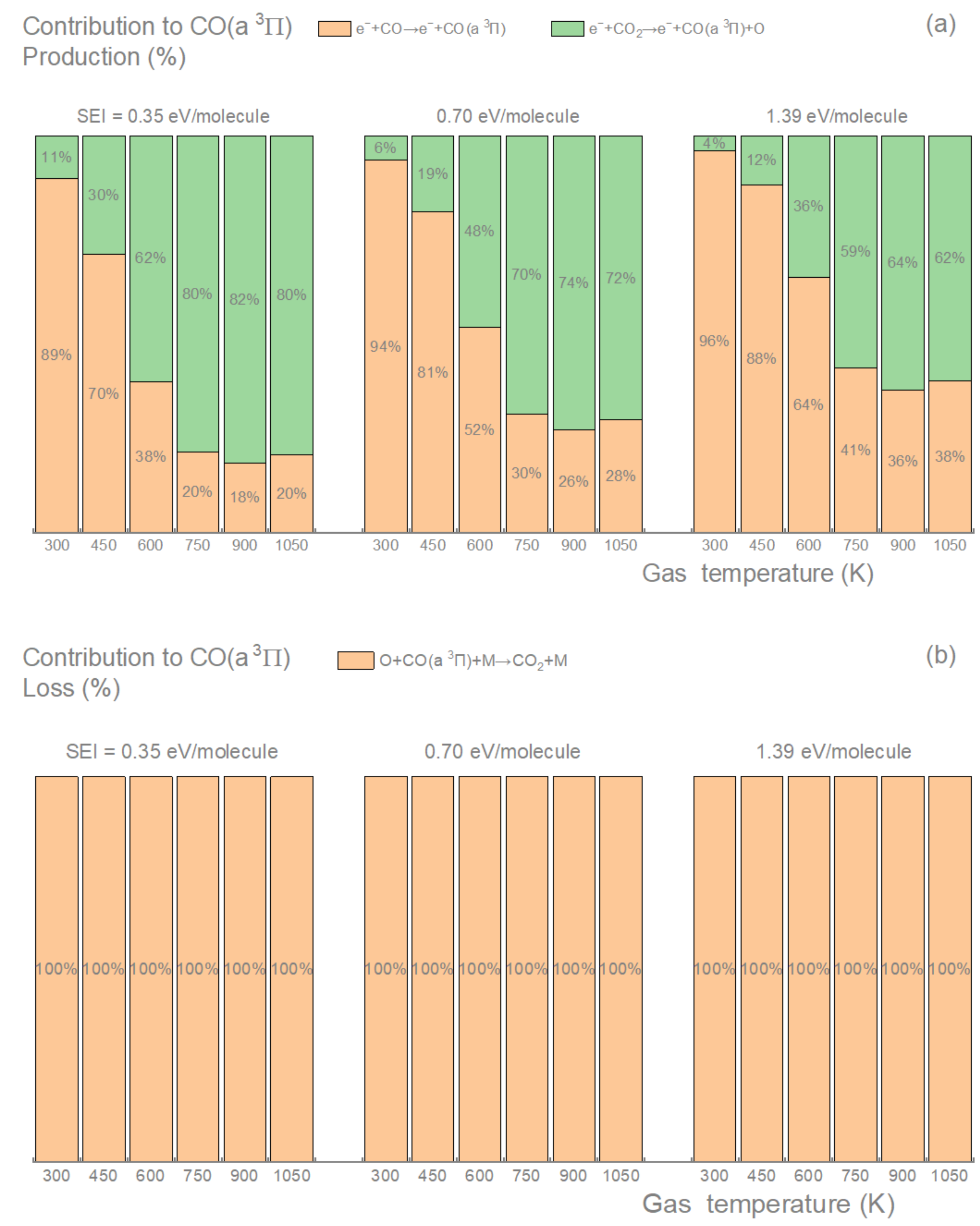

Fig. S17 Contribution of the elementary reactions in (a) production (b) consumption of CO(a $^3\Pi$) for T_g range of 300–1050 K and SEI = 0.35, 0.70 and 1.39 eV/molecule.

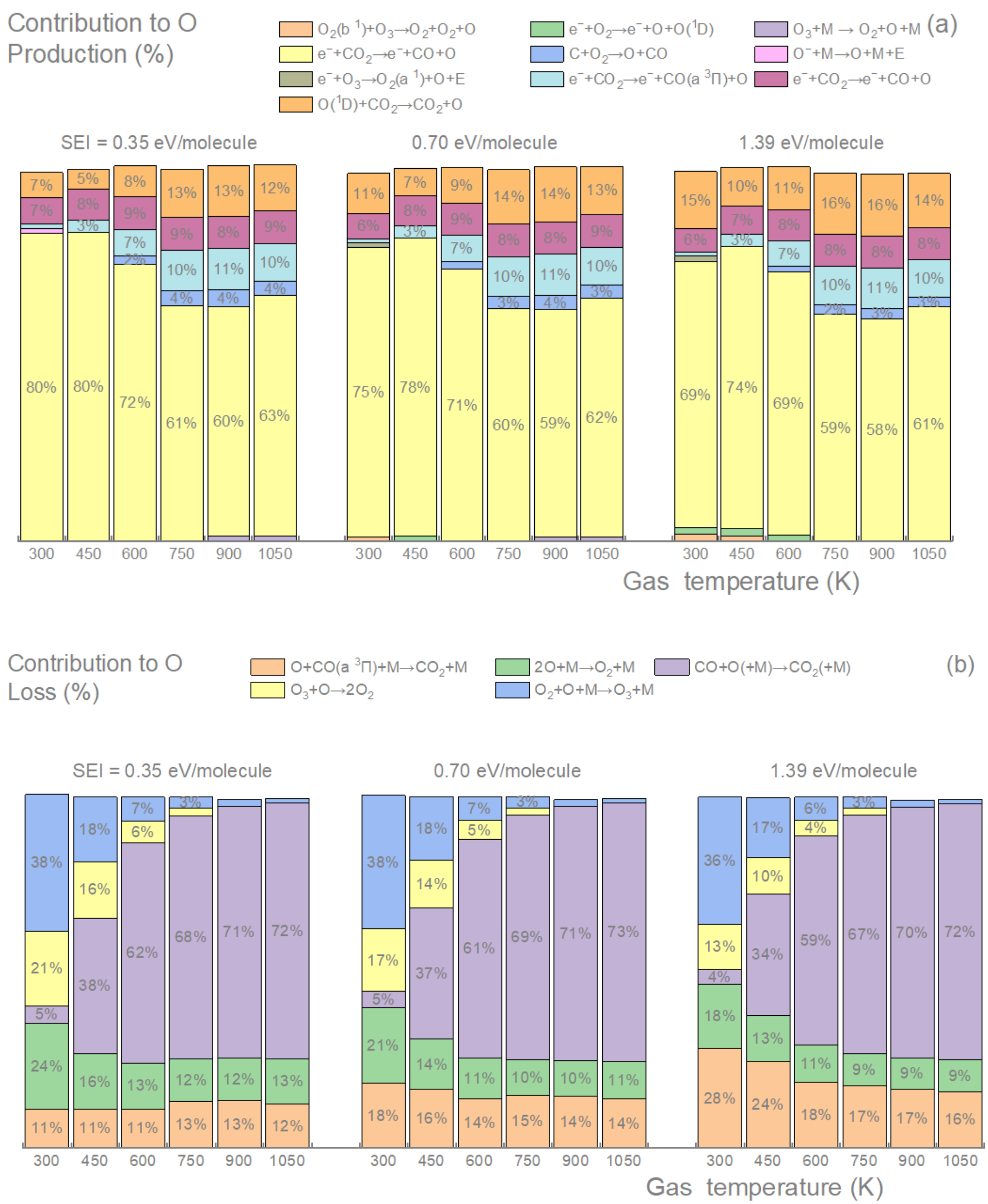

Fig. S18 Contribution of the elementary reactions in (a) production (b) consumption of O for T_g range of 300–1050 K and SEI = 0.35, 0.70 and 1.39 eV/molecule.

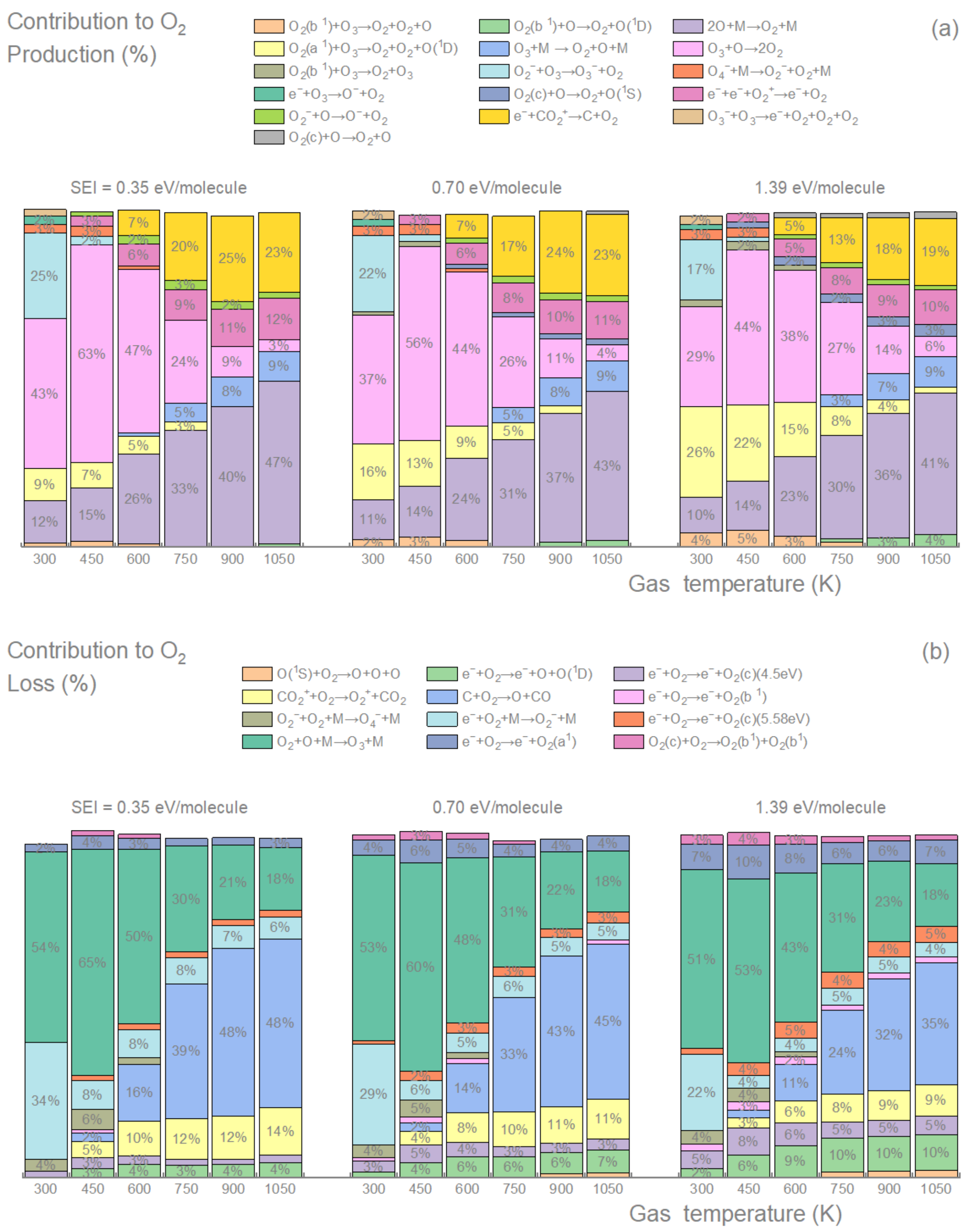

Fig. S19 Contribution of the elementary reactions in (a) production (b) consumption of O_2 for T_g range of 300–1050 K and SEI = 0.35, 0.70 and 1.39 eV/molecule.

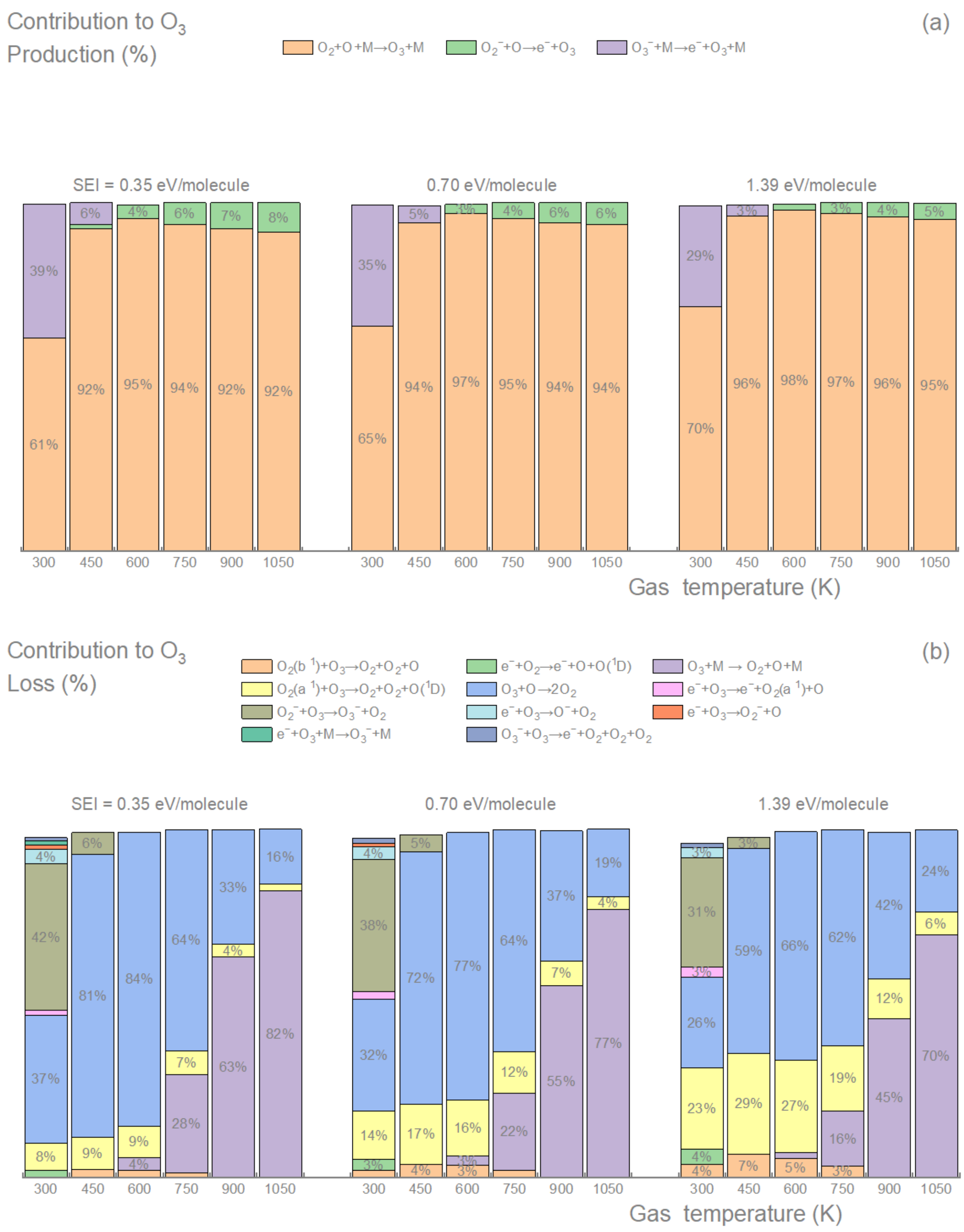

Fig. S20 Contribution of the elementary reactions in (a) production (b) consumption of O_3 for T_g range of 300–1050 K and SEI = 0.35, 0.70 and 1.39 eV/molecule.

4. Performance evaluation of developed mechanism

4.1. Energy efficiency calculation

The calculated energy efficiencies for the experimental and simulated CO_2 conversion for the temperature range of 300–1050 K and SEI = 0.35, 0.70 and 1.39 eV/molecule are given in this section.

Table S12 Energy efficiency calculations for experimental and simulated CO_2 conversions as compared to reaction enthalpy.

	**η (%) Experiments**				**η (%) Simulation**		
**T_g (K)**	0.35 eV/molecule	0.70 eV/molecule	1.39 eV/molecule	**T_g (K)**	0.35 eV/molecule	0.70 eV/molecule	1.39 eV/molecule
**300**	3.40	4.47	4.45	**300**	4.54	4.42	4.27
**450**	3.97	4.15	3.48	**450**	4.03	3.78	3.52
**600**	2.83	2.75	2.16	**600**	2.83	2.48	2.17
**750**	1.67	1.60	1.48	**750**	2.21	1.88	1.59
**900**	1.41	1.63	1.62	**900**	2.04	1.70	1.41
**1050**	0.66	1.39	1.52	**1050**	2.06	1.70	1.39

Table S13 Comparison of the CO_2 conversion, energy efficiency, and energy consumption for pure thermal and plasma-based CO_2 splitting.

P_{dis} (W)	P_{heat} (W)	T_g (K)	Conversion (%)	Efficiency (%)	Energy consumption at std. conditions (kJ/L)	(kJ/mol)	(eV/molecule)
20	0	300	2.0	4.3	6.0	134.4	1.39
0	20	2300	9.1	18.5	6.1	135.8	1.40
200	0	300	15.4	3.2	60.0	1344.8	13.9
0	24	2442	15.4	26.2	7.2	161.6	1.68
0	65	3600	90.3	55.9	19.4	434.1	4.50
0	80	5000	99.8	48.9	24.1	540.5	5.60

4.2. Reduced electric field study

To understand the effect of the reduced electric further and to ascertain the importance of thermal reactions, a study was conducted where for all the gas temperatures, the reduced electric field was kept constant in the numerical simulations. This study was conducted for a specific energy input of 1.39 eV/molecule and three different E/N values namely 100, 200, and 300 Td.

Comparison revealed similar decreasing trends in the conversion of CO_2 for all the E/N values. Thus, it can be understood that thermal reactions dominate as compared to electron-induced chemistry throughout all the temperatures studied. However, increasing the reduced electric field did have a positive impact on the conversion. This influence is more evident at lower temperatures, especially at 300 K when compared to the experimental values.

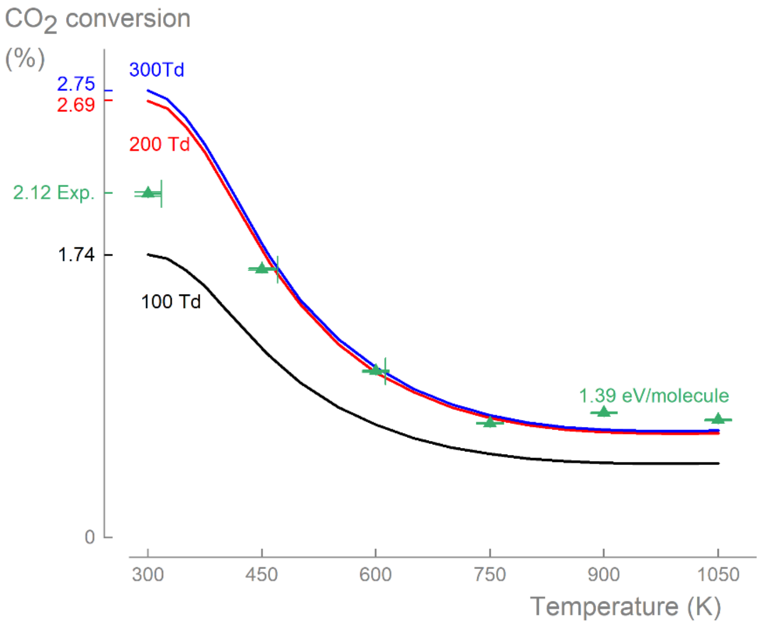

Fig. S21 Effect of reduced electric field on the conversion for fixed E/N = 100, 200, and 300 Td for SEI = 1.39 eV/molecule. All three cases showed similar trend of decrease in conversion ascertaining the predominance of thermal chemistry. The experimental data points (with varying E/N, see Fig. S6) are provided as a reference.

5. Detailed reaction mechanisms

5.1. Detailed list of excited species

Table S14: List of excited species considered as loss process in the mechanism

Ground State	Notation in the model	Described excited states
CO_2	CO_2 Va	(010)
	CO_2 Vb1	(100)
	CO_2 Vb2	(020)
	CO_2 Vc	(110), (030)
	CO_2 Vd	(n00), (0n0)
	CO_2 Ve	(040), (120), (011)
	CO_2 Vf	(200)
	CO_2 Vg	(050), (210), (130), (021), (101)
	CO_2 Vh	(300)
	CO_2 Vi	(060), (220), (140)
	CO_2 V1	(001)
		$CO_2(^1\Delta_u)$
CO	CO Vn	Sum of vibrations, n=1-10
	CO E2	CO(a $^1\Pi$)
	CO E31	CO(a $^3\Sigma_u^+$)
	CO E32	CO(b $^3\Sigma_u^+$)
	CO E41	CO(b $^1\Sigma_u^+$)
	CO E42	CO(c $^1\Sigma_u^+$)
	CO E43	CO(e $^1\Pi$)
O_2	O_2 Vn	Sum of vibrations, n=1-3

Since for a DBD, the vibrational excited states do not play an important role, the reactions involving the production of the species listed in Table S14 have been considered as loss processes i.e., the assumption that these excited species instantaneously de-excites.

5.2. Plasma chemistry mechanism

Table S15 Overview of the different reactions included in the CO_2 plasma reaction mechanism with the references from where the data was adopted. Most electron-impact reactions were treated by energy-dependent cross sections, f(σ). Unless otherwise stated, reaction rate coefficients are in the Arrhenius form,

for heavy particle collisions: k = A × T_{gas}^{n} × exp(−C/T_{gas}), for electron-impact collisions: k = A × T_{e}^{n} × exp(−C / T_{e}); both in molecule-cm^3-s-K-K units**.**

No.	Reaction	Rate coefficient	Ref
**SPECIES: CO_2**			
**Electron impact reactions**			
	Dissociative Attachment		
1	$e^- + CO_2 \rightarrow CO + O^-$	f(σ)	[17]
	Elastic / Effective / Momentum		
2	$e^- + CO_2 \rightarrow e^- + CO_2$	f(σ)	[17]
	Vibrational excitation		
3	$e^- + CO_2 \rightarrow e^- + CO_2$	f(σ)	[17]
	Electronic excitation / Dissociation		
4	$e^- + CO_2 \rightarrow e^- + CO + O$	f(σ)	[17,23]*
5	$e^- + CO_2 \rightarrow e^- + CO_2$	f(σ)	[17]**
6	$e^- + CO_2 \rightarrow e^- + CO(a\ ^3\Pi) + O$	f(σ)	[23]†
	Ionization		
7	$e^- + CO_2 \rightarrow e^- + e^- + CO_2^+$	f(σ)	[20]
8	$e^- + CO_2 \rightarrow e^- + e^- + CO^+ + O$	f(σ)	[20]
9	$e^- + CO_2 \rightarrow e^- + e^- + C^+ + O_2$	f(σ)	[20]
10	$e^- + CO_2 \rightarrow e^- + e^- + CO + O^+$	f(σ)	[20]
**SPECIES: CO**			
**Electron impact reactions**			
	Dissociative attachment		
11	$e^- + CO \rightarrow C + O^-$	f(σ)	[17]
	Elastic / Effective / Momentum		
12	$e^- + CO \rightarrow e^- + CO$	f(σ)	[17]
	Vibrational excitation		
13	$e^- + CO \rightarrow e^- + CO$	f(σ)	[17]

	Electronic excitation / Dissociation		
14	$e^- + CO \rightarrow e^- + CO$	f(σ)	[17]
15	$e^- + CO \rightarrow e^- + C + O$	f(σ)	[17]
16	$e^- + CO \rightarrow e^- + CO(a^3\Pi)$	f(σ)	[17]‡
	Ionization		
17	$e^- + CO \rightarrow e^- + e^- + CO^+$	f(σ)	[21]
18	$e^- + CO \rightarrow e^- + e^- + C^+ + O$	f(σ)	[21]
19	$e^- + CO \rightarrow e^- + e^- + C + O^+$	f(σ)	[21]
**Carbon species ion-neutral reactions**			
20	$CO + O^- \rightarrow e^- + CO_2$	$5.8\times 10^{-10} \times T_{gas}^{-0.39}$	[27]
21	$C + O^- \rightarrow e^- + CO$	5.0×10^{-10}	[28]
22	$O^- + M \rightarrow e^- + O + M$	$2.3\times10^{-9} \times \exp(-26000/T_{gas})$	[27]
23	$C^+ + CO_2 \rightarrow CO^+ + CO$	1.1×10^{-9}	[28]
24	$C^+ + CO \rightarrow CO^+ + C$	5.0×10^{-13}	[29]
25	$C^+ + O_2 \rightarrow CO + O^+$	6.2×10^{-10}	[28]
26	$C^+ + O_2 \rightarrow CO^+ + O$	3.8×10^{-10}	[28]
27	$CO_2^+ + O \rightarrow O_2^+ + CO$	1.64×10^{-10}	[27]
28	$CO_2^+ + O \rightarrow CO_2 + O^+$	9.62×10^{-11}	[27]
29	$CO_2^+ + O_2^- \rightarrow CO + O_2 + O$	6×10^{-7}	[27]
30	$CO_2^+ + O_2 \rightarrow CO_2 + O_2^+$	5.3×10^{-11}	[27]
31	$CO_2^+ + O_2(a\ ^1) \rightarrow CO_2 + O_2^+$	6.3×10^{-11}	[11]
32	$CO_2^+ + O_2(b\ ^1) \rightarrow CO_2 + O_2^+$	6.3×10^{-11}	[11]
33	$CO_2^+ + O_2(c) \rightarrow CO_2 + O_2^+$	7.04×10^{-11}	[11]
34	$CO^+ + CO_2 \rightarrow CO_2^+ + CO$	1×10^{-9}	[27]
35	$CO^+ + O_2 \rightarrow CO + O_2^+$	1.2×10^{-10}	[27]
36	$CO^+ + O \rightarrow CO + O^+$	1.4×10^{-10}	[29]
37	$CO^+ + O_2(a\ ^1) \rightarrow CO + O_2^+$	1.43×10^{-10}	[11]
38	$CO^+ + O_2(b\ ^1) \rightarrow CO + O_2^+$	1.43×10^{-10}	[11]

39	$CO^+ + O_2(c) \rightarrow CO + O_2^+$	1.59×10^{-10}	[11]
40	$CO^+ + C \rightarrow CO + C^+$	1.1×10^{-10}	[29]
41	$C^+ + O_2(a\ ^1) \rightarrow CO + O^+$	4.54×10^{-10}	[11]
42	$C^+ + O_2(b\ ^1) \rightarrow CO + O^+$	4.54×10^{-10}	[11]
43	$C^+ + O_2(c) \rightarrow CO + O^+$	4.54×10^{-10}	[11]
44	$C^+ + O_2(a\ ^1) \rightarrow CO^+ + O$	3.8×10^{-10}	[11]
45	$C^+ + O_2(b\ ^1) \rightarrow CO^+ + O$	3.8×10^{-10}	[11]
46	$C^+ + O_2(c) \rightarrow CO^+ + O$	3.8×10^{-10}	[11]
47	$O^+ + CO_2 \rightarrow O + CO_2^+$	4.5×10^{-10}	[27]
48	$O^+ + CO_2 \rightarrow O_2^+ + CO$	4.5×10^{-10}	[27]
49	$O^+ + CO \rightarrow O + CO^+$	$4.9 \times 10^{-12} \times (T_{gas}/300)^{0.5} \times \exp(-4580/T_{gas})$	[29]
50	$O_2^+ + C \rightarrow CO^+ + O$	5.2×10^{-11}	[27]
51	$O_4^- + M \rightarrow O_2^- + O_2 + M$	4.0×10^{-12}	[29]
52	$O_3^- + O_2 \rightarrow O_2 + O_3 + e^-$	2.3×10^{-11}	[29]
53	$O_3^- + O \rightarrow O_3 + O^-$	1.0×10^{-13}	[29]
54	$O_3^- + O_3 \rightarrow 3O_2 + e^-$	3.0×10^{-10}	[29]
55	$O_3^- + M \rightarrow O_3 + M + e^-$	2.3 × 10−11	[29]
**Carbon species electron-ion recombination**			
56	$e^- + CO_2^+ \rightarrow CO + O$	$2.0 \times 10^{-5} \times T_e^{-0.5}\, T_{gas}^{-0.1}$	[27]
57	$e^- + CO_2^+ \rightarrow C + O_2$	$1.07 \times 10^{-5} \times T_e^{-0.5}\, T_{gas}^{-0.1}$	[27]
58	$e^- + CO^+ \rightarrow C + O$	$3.46 \times 10^{-8} \times T_e^{-0.48}$	[27]
59	$e^- + CO_2^+ \rightarrow CO + O(^1D)$	$4.2 \times 10^{-7} \times (300/T_e)^{0.75}$	[30]
60	$e^- + CO_2^+ \rightarrow CO + O(^1S)$	$4.2 \times 10^{-7} \times (300/T_e)^{0.75}$	[30]
**Carbon species neutral-neutral and quenching reactions**			
61	$O(^1S) + CO_2 \rightarrow O(^1D) + CO_2$	4.0×10^{-13}	[30]
62	$O(^1S) + CO \rightarrow O(^1D) + CO$	1.0×10^{-13}	[30]
63	$O(^1D) + CO_2 \rightarrow CO + O_2$	2.4×10^{-13}	[31]
64	$O(^1D) + CO_2 \rightarrow CO_2 + O$	$7.9 \times 10^{-11} \times \exp(133/T_{gas})$	[27]

65	$O(^1D) + CO \rightarrow CO + O$	$4.7 \times 10^{-11} \times \exp(62.542/T_{gas})$	[27]
66	$O(^1D) + CO \rightarrow CO_2$	8.00×10^{-11}	[28]
67	$O_2(a\ ^1) + C \rightarrow CO + O$	3.00×10^{-11}	[32]
68	$O_2(b\ ^1) + C \rightarrow CO + O$	3.00×10^{-11}	[32]
69	$O_2(c) + C \rightarrow CO + O$	3.00×10^{-11}	[32]
70	$CO(a\ ^3\Pi) + O_2 \rightarrow CO + O + O$	2.4×10^{-11}	[27] ‡
71	$CO(a\ ^3\Pi) + O_2 \rightarrow CO_2 + O$	$4.2 \times 10^{-12} \times \exp(-24000/T_{gas})$	[11] ‡
72	$CO(a\ ^3\Pi) + CO \rightarrow CO_2 + C$	9.12×10^{-13}	[27] ‡
73	$CO(a\ ^3\Pi) + CO_2 \rightarrow CO + CO + O$	5.0×10^{-12}	[27] ‡
74	$CO(a\ ^3\Pi) + O \rightarrow CO + O$	1.9×10^{-10}	[27] ‡
75	$CO(a\ ^3\Pi) + O_2 \rightarrow CO + O_2$	11.0×10^{-11}	[27] ‡
76	$CO(a\ ^3\Pi) + CO \rightarrow CO + CO$	9.85×10^{-11}	[27] ‡
77	$CO(a\ ^3\Pi) + CO_2 \rightarrow CO + CO_2$	5.0×10^{-12}	[27] ‡
78	$CO(a\ ^3\Pi) + O + M \rightarrow CO_2 + M$	$1.08 \times 10^{-4} \times T_{gas}^{-3.1} \times \exp(-2772/T_{gas})$	[This work] ‡
79	$CO(a\ ^3\Pi) + O_3 \rightarrow CO_2 + O_2$	4.0×10^{-25}	[11] ‡
80	$CO(a\ ^3\Pi) + O^+ \rightarrow CO^+ + O$	$1.58 \times 10^{-11} \times (T_{gas}/300)^{0.5} \times \exp(-4580/T_{gas})$	[11] ‡
81	$CO(a\ ^3\Pi) + O^- \rightarrow e^- + CO_2$	5.5×10^{-10}	[11] ‡
82	$CO(a\ ^3\Pi) + C^+ \rightarrow CO^+ + C$	1.62×10^{-12}	[11] ‡
**SPECIES: C**			
83	$e^- + C \rightarrow e^- + e^- + C^+$	f(σ)	[33]
**SPECIES: O_2**			
**Electron impact reactions:**			
	Elastic / effective / momentum		
84	$e^- + O \rightarrow e^- + O$	f(σ)	[17, 34]
85	$e^- + O_2 \rightarrow e^- + O_2$	f(σ)	[17, 34]
86	$e^- + O_3 \rightarrow e^- + O_3$	f(σ)	[17, 34]
			Electron attachment
87	$e^- + O_2 \rightarrow O^- + O$	f(σ)	[35]

88	$e^- + O_3 \rightarrow O^- + O_2$	f(σ)	[33]
89	$e^- + O_3 \rightarrow O_2^- + O$	f(σ)	[33]
90	$e^- + O_2 + M \rightarrow O_2^- + M$	f(σ)	[36]
91	$e^- + O + O_2 \rightarrow O^- + O_2$	1.0×10^{-31}	[37]
92	$e^- + O_3 + M \rightarrow O_3^- + M$	1.0×10^{-31}	[37]
	Electron detachment		
93	$O^- + O \rightarrow O_2 + e^-$	1.4×10^{-10}	[37]
94	$O^- + O_2 \rightarrow O_3 + e^-$	5.0×10^{-15}	[37]
95	$O^- + O_2(a\ ^1) \rightarrow O_3 + e^-$	3.0×10^{-10}	[37]
96	$O^- + O_2(b\ ^1) \rightarrow O + O_2 + e^-$	6.9×10^{-10}	[37]
97	$O^- + O_3 \rightarrow O_2 + O_2 + e^-$	3.0×10^{-10}	[37]
98	$O_2^- + O \rightarrow O_3 + e^-$	1.5×10^{-10}	[37]
99	$O_2^- + O_2 \rightarrow O_2 + O_2 + e^-$	$2.7 \times 10^{-10} \times (T_{effN2} / 300)^{0.5} \times \exp(-5590 / T_{effN2})$	[37][c]
100	$O_2^- + O_2(a\ ^1) \rightarrow O_2 + O_2 + e^-$	2.0×10^{-10}	[37]
101	$O_2^- + O_2(b\ ^1) \rightarrow O_2 + O_2 + e^-$	3.6×10^{-10}	[37]
102	$O_3^- + O \rightarrow O_2 + O_2 + e^-$	3.0×10^{-10}	[37]
	Vibrational excitation		
103	$e^- + O_2 \rightarrow e^- + O_2(v1)$	f(σ)	[35]
104	$e^- + O_2 \rightarrow e^- + O_2(v2)$	f(σ)	[35]
105	$e^- + O_2 \rightarrow e^- + O_2(v3)$	f(σ)	[35]
	Electronic excitation / de-excitation / dissociation		
106	$e^- + O \rightarrow e^- + O(^1D)$	f(σ)	[17, 34]
107	$e^- + O(^1D) \rightarrow e^- + O$	f(σ)	[17, 34]
108	$e^- + O \rightarrow e^- + O(^1S)$	f(σ)	[17, 34]
109	$e^- + O(^1S) \rightarrow e^- + O$	f(σ)	[17, 34]
110	$e^- + O \rightarrow e^- + O$	f(σ)	[17, 34]
111	$e^- + O \rightarrow e^- + O$	f(σ)	[17, 34]
112	$e^- + O \rightarrow e^- + O$	f(σ)	[17, 34]
113	$e^- + O \rightarrow e^- + O$	f(σ)	[17, 34]

114	$e^- + O_2 \rightarrow e^- + O_2(a\ ^1)$	f(σ)	[35]
115	$e^- + O_2 \rightarrow e^- + O_2(b\ ^1)$	f(σ)	[35]
116	$e^- + O_2 \rightarrow e^- + O_2(c)$ (threshold = 4.5 eV)	f(σ)	[38]
117	$e^- + O_2 \rightarrow e^- + O_2(c)$ (threshold = 5.58 eV)	f(σ)	[38]
118	$e^- + O_2 \rightarrow e^- + O + O(^1D)$	f(σ)	[39]
119	$e^- + O_2 \rightarrow e^- + O + O(^1S)$ (threshold = 9.97 eV)	f(σ)	[17, 34]
120	$e^- + O_2 \rightarrow e^- + O + O(^1S)$ (threshold = 14.7 eV)	f(σ)	[17, 34]
121	$e^- + O_2(a\ ^1) \rightarrow e^- + O_2(b\ ^1)$	f(σ)	[38]
122	$e^- + O_2(a\ ^1) \rightarrow e^- + O_2(c)$	f(σ)	[38]
123	$e^- + O_2(b\ ^1) \rightarrow e^- + O_2(c)$	f(σ)	[38]
124	$e^- + O_2(a\ ^1) \rightarrow e^- + O + O$	f(σ)	[38]
125	$e^- + O_2(b\ ^1) \rightarrow e^- + O + O$	f(σ)	[38]
126	$e^- + O_2(a\ ^1) \rightarrow e^- + O + O(^1D)$	f(σ)	[38]
127	$e^- + O_2(b\ ^1) \rightarrow e^- + O + O(^1D)$	f(σ)	[38]
128	$e^- + O_3 \rightarrow e^- + O_2(a\ ^1) + O$	8.5 × f(σ)	[38]
129	$e^- + O_3 \rightarrow e^- + O_2(a\ ^1) + O(^1D)$	8.5 × f(σ)	[39]
130	$e^- + O_3 \rightarrow e^- + O_2(a\ ^1) + O(^1S)$ (threshold = 9.97 eV)	8.5 × f(σ)	[17, 34]
131	$e^- + O_3 \rightarrow e^- + O_2(a\ ^1) + O(^1S)$ (threshold = 14.7 eV)	8.5 × f(σ)	[17,34]
	Ionization		
132	$e^- + O \rightarrow e^- + e^- + O^+$	f(σ)	[35]
133	$e^- + O_2 \rightarrow e^- + e^- + O_2^+$	f(σ)	[35]
134	$e^- + O_2 \rightarrow e^- + e^- + O + O^+$	f(σ)	[35]
135	$e^- + O_2(a\ ^1) \rightarrow e^- + e^- + O_2^+$	f(σ)	[38]
136	$e^- + O_2(b\ ^1) \rightarrow e^- + e^- + O_2^+$	f(σ)	[38]
137	$e^- + O_2(a\ ^1) \rightarrow e^- + e^- + O + O^+$	f(σ)	[38]
138	$e^- + O_2(b\ ^1) \rightarrow e^- + e^- + O + O^+$	f(σ)	[38]
**Electron-ion recombination:**			
139	$e^- + O^+ + M \rightarrow O + M$	$6.0 \times 10^{-27} \times (300 / T_e)^{1.5}$	[37]

140	$e^- + e^- + O^+ \rightarrow e^- + O$	$7.0 \times 10^{-20} \times (300 / T_e)^{4.5}$	[37]
141	$e^- + e^- + O_2^+ \rightarrow e^- + O_2$	$7.0 \times 10^{-20} \times (300 / T_e)^{4.5}$	[37]
142	$e^- + O_2^+ + M \rightarrow O_2 + M$	$6.0 \times 10^{-27} \times (300 / T_e)^{1.5}$	[37]
143	$e^- + O_2^+ \rightarrow O + O$	$0.55 \times 2.7 \times 10^{-7} \times (300 / T_e)^{0.7}$	[37]
144	$e^- + O_2^+ \rightarrow O + O(^1D)$	$0.40 \times 2.7 \times 10^{-7} \times (300 / T_e)^{0.7}$	[37]
145	$e^- + O_2^+ \rightarrow O + O(^1S)$	$0.05 \times 2.7 \times 10^{-7} \times (300 / T_e)^{0.7}$	[37]
146	$e^- + O_4^+ \rightarrow O_2 + O_2$	$1.4 \times 10^{-6} \times (300 / T_e)^{0.5}$	[37]
**Neutral-neutral reactions:**			
	Reactions with excited species		
147	$O(^1D) + O \rightarrow O + O$	8.0×10^{-12}	[37]
148	$O(^1D) + O_2 \rightarrow O + O_2$	$6.4 \times 10^{-12} \times \exp(67 / T_{gas})$	[37]
149	$O(^1D) + O_2 \rightarrow O + O_2(a\ ^1)$	1.0×10^{-12}	[37]
150	$O(^1D) + O_2 \rightarrow O + O_2(b\ ^1)$	$2.6 \times 10^{-11} \times \exp(67 / T_{gas})$	[37]
151	$O(^1D) + O_3 \rightarrow O_2 + O + O$	1.2×10^{-10}	[37]
152	$O(^1D) + O_3 \rightarrow O_2 + O_2$	1.2×10^{-10}	[37]
153	$O(^1D) + O_3 \rightarrow O + O_3$	1.2×10^{-10}	[37]
154	$O(^1S) + O \rightarrow O + O$	$3.33 \times 10^{-11} \times \exp(-300 / T_{gas})$	[40]
155	$O(^1S) + O \rightarrow O(^1D) + O$	$5.0 \times 10^{-11} \times \exp(-301 / T_{gas})$	[42]
156	$O(^1S) + O_2 \rightarrow O + O_2$	$1.6 \times 10^{-12} \times \exp(-850 / T_{gas})$	[40]
157	$O(^1S) + O_2 \rightarrow O(^1D) + O_2$	$1.3 \times 10^{-12} \times \exp(-850 / T_{gas})$	[37]
158	$O(^1S) + O_2 \rightarrow O + O + O$	$3.0 \times 10^{-12} \times \exp(-850 / T_{gas})$	[37]
159	$O(^1S) + O_2(a\ ^1) \rightarrow O + O_2(c)$	1.1×10^{-10}	[37]
160	$O(^1S) + O_2(a\ ^1) \rightarrow O(^1D) + O_2(b\ ^1)$	2.9×10^{-11}	[37]
161	$O(^1S) + O_2(a\ ^1) \rightarrow O + O + O$	3.2×10^{-11}	[37]
162	$O(^1S) + O_3 \rightarrow O_2 + O_2$	2.9×10^{-10}	[37]
163	$O(^1S) + O_3 \rightarrow O_2 + O + O(^1D)$	2.9×10^{-10}	[37]
164	$O_2(a\ ^1) + O \rightarrow O_2 + O$	7.0×10^{-16}	[37]
165	$O_2(a\ ^1) + O_2 \rightarrow O_2 + O_2$	$3.8 \times 10^{-18} \times \exp(-205 / T_{gas})$	[37]
166	$O_2(a\ ^1) + O_3 \rightarrow O_2 + O_2 + O(^1D)$	$5.2 \times 10^{-11} \times \exp(-2840 / T_{gas})$	[37]

167	$O_2(a\ ^1) + O_2(a\ ^1) \rightarrow O_2 + O_2(b\ ^1)$	$7.0 \times 10^{-28} \times (T_{gas})^{3.8} \times \exp(700 / T_{gas})$	[37]
168	$O_2(a\ ^1) + O_2 \rightarrow O_3 + O$	$2.95 \times 10^{-21} \times (T_{gas} / 300)^{0.5}$	[40]
169	$O_2(a\ ^1) + O_2(a\ ^1) \rightarrow O_2 + O_2$	$9.0 \times 10^{-17} \times \exp(-560 / T_{gas})$	[40]
170	$O_2(a\ ^1) + O_2 + O \rightarrow O_2 + O_2 + O$	1.0×10^{-32}	[40]
171	$O_2(b\ ^1) + O \rightarrow O_2(a\ ^1) + O$	8.1×10^{-14}	[37]
172	$O_2(b\ ^1) + O \rightarrow O_2 + O(^1D)$	$3.4 \times 10^{-11} \times (300 / T_{gas})^{0.1} \times \exp(-4200 / T_{gas})$	[37]
173	$O_2(b\ ^1) + O_2 \rightarrow O_2(a\ ^1) + O_2$	$4.3 \times 10^{-22} \times T_{gas}^{2.4} \times \exp(-281 / T_{gas})$	[37]
174	$O_2(b\ ^1) + O_3 \rightarrow O_2 + O_2 + O$	2.2×10^{-11}	[37]
175	$O_2(b\ ^1) + O \rightarrow O_2(a\ ^1) + O$	$8.0 \times 10^{-15} \times (T_{gas} / 300)^{0.5}$	[42]
176	$O_2(b\ ^1) + O_2 \rightarrow O_2(a\ ^1) + O_2$	$3.6 \times 10^{-17} \times (T_{gas} / 300)^{0.5}$	[42]
177	$O_2(b\ ^1) + O_3 \rightarrow O_2(a\ ^1) + O_2 + O$	1.0×10^{-14}	[42]
178	$O_2(b\ ^1) + O_3 \rightarrow O_2 + O_3$	2.2×10^{-11}	[42]
179	$O_2(b\ ^1) + O_3 \rightarrow O_2(a\ ^1) + O_3$	2.2×10^{-11}	[42]
180	$O_2(b\ ^1) + O_2 \rightarrow O_2 + O_2$	4.1×10^{-17}	[43]
181	$O_2(c) + O \rightarrow O_2 + O(^1S)$	9.0×10^{-12}	[37]
182	$O_2(c) + O_2 \rightarrow O_2(b\ ^1) + O_2(b\ ^1)$	3.0×10^{-13}	[37]
183	$O_2(c) + O \rightarrow O_2 + O$	4.95×10^{-12}	[44]
184	$O_2(c) + O \rightarrow O_2(a\ ^1) + O(^1D)$	2.7×10^{-12}	[44]
185	$O_2(c) + O \rightarrow O_2(b\ ^1) + O(^1D)$	1.35×10^{-12}	[44]
186	$O_2(c) + O_2 \rightarrow O_2(a\ ^1) + O_2$	1.86×10^{-13}	[44]
187	$O_2(c) + O_2 \rightarrow O_2(b\ ^1) + O_2$	2.1×10^{-14}	[44]
188	$O_2(c) + O_2 \rightarrow O_2 + O_2$	2.3×10^{-14}	[44]
	Radiative decay		
189	$O_2(a\ ^1) \rightarrow O_2$	2.6×10^{-4}	[37]
190	$O_2(b\ ^1) \rightarrow O_2$	1.5×10^{-3}	[37]
191	$O_2(b\ ^1) \rightarrow O_2(a\ ^1)$	8.5×10^{-2}	[37]
192	$O_2(c) \rightarrow O_2$	11.0	[37]

193	$O(^1D) \rightarrow O$	5.0×10^{-3}	[40]
194	$O(^1S) \rightarrow O(^1D)$	1.34	[40]
**Ion-neutral reactions:**			
	Two-body collisions		
195	$O^+ + O_2 \rightarrow O_2^+ + O$	$2.0 \times 10^{-11} \times (300 / T_{effN})^{0.5}$	[37][a]
196	$O^+ + O_3 \rightarrow O_2^+ + O_2$	1.0×10^{-10}	[37]
197	$O_4^+ + O_2 \rightarrow O_2^+ + O_2 + O_2$	$3.3 \times 10^{-6} \times (300 / T_{effN4})^{4.0} \times \exp(-5053 / T_{effN4})$	[37][a]
198	$O_4^+ + O_2(a\ ^1) \rightarrow O_2^+ + O_2 + O_2$	1.0×10^{-10}	[37]
199	$O_4^+ + O_2(b\ ^1) \rightarrow O_2^+ + O_2 + O_2$	1.0×10^{-10}	[37]
200	$O_4^+ + O \rightarrow O_2^+ + O_3$	3.0×10^{-10}	[37]
201	$O_2^+ + O_2 + O_2 \rightarrow O_4^+ + O_2$	$2.4 \times 10^{-30} \times (300 / T_{effN2})^{3.2}$	[37][a]
202	$O^- + O_2(a\ ^1) \rightarrow O_2^- + O$	1.0×10^{-10}	[37]
203	$O^- + O_3 \rightarrow O_3^- + O$	8.0×10^{-10}	[37]
204	$O_2^- + O \rightarrow O^- + O_2$	3.3×10^{-10}	[37]
205	$O_2^- + O_3 \rightarrow O_3^- + O_2$	3.5×10^{-10}	[37]
206	$O_3^- + O \rightarrow O_2^- + O_2$	1.0×10^{-11}	[37]
207	$O_4^- + O_2 \rightarrow O_2^- + O_2 + O_2$	$1.0 \times 10^{-10} \times \exp(-1044 / T_{effN4})$	[37][a]
208	$O_4^- + O \rightarrow O_3^- + O_2$	4.0×10^{-10}	[37]
209	$O_4^- + O \rightarrow O^- + O_2 + O_2$	3.0×10^{-10}	[37]
210	$O_4^- + O_2(a\ ^1) \rightarrow O_2^- + O_2 + O_2$	1.0×10^{-10}	[37]
211	$O_4^- + O_2(b\ ^1) \rightarrow O_2^- + O_2 + O_2$	1.0×10^{-10}	[37]
	Three-body collisions		
212	$O^+ + O + M \rightarrow O_2^+ + M$	1.0×10^{-29}	[37]
213	$O^- + O_2 + M \rightarrow O_3^- + M$	$1.1 \times 10^{-30} \times (300 / T_{effN})$	[37][a]
214	$O_2^- + O_2 + M \rightarrow O_4^- + M$	$3.5 \times 10^{-31} \times (300 / T_{effN2})$	[41][a]
**Ion-ion reactions:**			
	Two-body collisions		
215	$O^- + O^+ \rightarrow O + O$	$2.0 \times 10^{-7} \times (300 / T_{ionN})^{0.5}$	[41][a]

216	$O^- + O_2^+ \rightarrow O + O_2$	$2.0 \times 10^{-7} \times (300 / T_{ionN})^{0.5}$	[41][a]
217	$O_2^- + O^+ \rightarrow O_2 + O$	$2.0 \times 10^{-7} \times (300 / T_{ionN})^{0.5}$	[41][a]
218	$O_2^- + O_2^+ \rightarrow O_2 + O_2$	$2.0 \times 10^{-7} \times (300 / T_{ionN})^{0.5}$	[41][a]
219	$O_3^- + O^+ \rightarrow O_3 + O$	$2.0 \times 10^{-7} \times (300 / T_{ionN})^{0.5}$	[41][a]
220	$O_3^- + O_2^+ \rightarrow O_3 + O_2$	$2.0 \times 10^{-7} \times (300 / T_{ionN})^{0.5}$	[41][a]
221	$O^- + O_2^+ \rightarrow O + O + O$	1.0×10^{-7}	[41]
222	$O^- + O_4^+ \rightarrow O + O_2 + O_2$	1.0×10^{-7}	[41]
223	$O_2^- + O_2^+ \rightarrow O_2 + O + O$	1.0×10^{-7}	[41]
224	$O_2^- + O_4^+ \rightarrow O_2 + O_2 + O_2$	1.0×10^{-7}	[41]
225	$O_3^- + O_2^+ \rightarrow O_3 + O + O$	1.0×10^{-7}	[41]
226	$O_3^- + O_4^+ \rightarrow O_3 + O_2 + O_2$	1.0×10^{-7}	[41]
227	$O_4^- + O^+ \rightarrow O_2 + O_2 + O$	1.0×10^{-7}	[41]
228	$O_4^- + O_2^+ \rightarrow O_2 + O_2 + O_2$	1.0×10^{-7}	[41]
229	$O_4^- + O_4^+ \rightarrow O_2 + O_2 + O_2 + O_2$	1.0×10^{-7}	[41]
	Three-body collisions		
230	$O^- + O^+ + M \rightarrow O + O + M$	$2.5 \times 10^{-25} \times (300 / T_{ionN})^{2.5}$	[41][a]
231	$O^- + O_2^+ + M \rightarrow O + O_2 + M$	$2.5 \times 10^{-25} \times (300 / T_{ionN})^{2.5}$	[41][a]
232	$O_2^- + O^+ + M \rightarrow O_2 + O + M$	$2.5 \times 10^{-25} \times (300 / T_{ionN})^{2.5}$	[41][a]
233	$O_2^- + O_2^+ + M \rightarrow O_2 + O_2 + M$	$2.5 \times 10^{-25} \times (300 / T_{ionN})^{2.5}$	[41][a]
234	$O^- + O^+ + M \rightarrow O_2 + M$	$2.5 \times 10^{-25} \times (300 / T_{ionN})^{2.5}$	[41][a]
235	$O^- + O_2^+ + M \rightarrow O_3 + M$	$2.5 \times 10^{-25} \times (300 / T_{ionN})^{2.5}$	[41][a]
236	$O_2^- + O^+ + M \rightarrow O_3 + M$	$2.5 \times 10^{-25} \times (300 / T_{ionN})^{2.5}$	[41][a]
237	$O_3^- + O^+ + M \rightarrow O_3 + O + M$	$2.5 \times 10^{-25} \times (300 / T_{ionN2})^{2.5}$	[41][a]
238	$O_3^- + O_2^+ + M \rightarrow O_3 + O_2 + M$	$2.5 \times 10^{-25} \times (300 / T_{ionN2})^{2.5}$	[41][a]

[a] The reaction rate coefficients are as a function of different temperature:

$$T_{effN} = (T_{ionN} + 0.5 \times T_{gas}) \div (1.0 + 0.5)$$
$$T_{effN2} = (T_{ionN2} + 1.0 \times T_{gas}) \div (1.0 + 1.0)$$
$$T_{effN4} = (T_{ionN4} + 2.0 \times T_{gas}) \div (1.0 + 2.0)$$
$$T_{ionN} = T_{gas} + dT_{ion} \times 14 \times (8.0 \times 10^{19})^2$$
$$T_{ionN2} = T_{gas} + dT_{ion} \times 28 \times (4.1 \times 10^{19})^2$$
$$T_{ionN4} = T_{gas} + dT_{ion} \times 56 \times (7.0 \times 10^{19})^2$$
$$dT_{ion} = 2.0 \div (3.0 \times 1.3807 \times 10^{-16}) \times 1.6605 \times 10^{-24} \times (E/N \times 10^{-17})^2$$

*Electronic excitation at 7 eV. [17] for Pure-IST ; [23] for IST-Polak with CO($a^3\Pi$)

**Electronic excitation at 10.5 eV only for "Pure-IST" mechanism

†Electronic excitation at 11.9 eV [23] for "IST-Polak with CO($a^3\Pi$)" mechanism

‡Applicable to only "IST-Polak with CO($a^3\Pi$)" mechanism

5.3. Thermal chemistry mechanism

Table S16: Overview of the different reactions included in the thermal reaction mechanism. The reaction rate coefficients are in the Arrhenius form: $k = A \times T_{gas}^{n} \times \exp(-E_a / R \times T_g)$, in mol-cm^3-s-cal-K units.

No.	Reaction	Rate coefficient	Ref
**Conventional (thermal) reactions:**			
1	$CO + O_2 \rightarrow O + CO_2$	$1.533 \times 10^{12} \times \exp(-47700.0/ R \times T_{gas})$	[24]
2	$O + CO_2 \rightarrow CO + O_2$	Reverse reaction rate	
3	$C + O_2 \rightarrow O + CO_2$	$6.62 \times 10^{13} \times \exp(-636.0/ R \times T_{gas})$	[45]
4	$O + CO_2 \rightarrow C + O_2$	Reverse reaction rate	
5	$CO + O (+ M) \rightarrow CO_2 (+ M)$	$1.88 \times 10^{11} \times \exp(-2430/ R \times T_{gas})$<br>$1.40 \times 10^{21} \times T_{gas}^{-2.1} \times \exp(-5500/ R \times T_{gas})$<br>CO enhanced by 1.90<br>$CO_2$ enhanced by 3.8	[46]
6	$CO_2 (+ M) \rightarrow CO + O (+ M)$	Reverse reaction rate	
7	$O + O + M \rightarrow O_2 + M$	$1.89 \times 10^{13} \times \exp(1788.3/ R \times T_{gas})$<br>CO enhanced by 2.108<br>$CO_2$ enhanced by 4.34<br>O / O(^{1}D) / O(^{1}S) / O_2 / O_2(a ^{1}) / O_2(b ^{1}) / O_2(c) / O_3 enhanced by 1.21	[24]
8	$O_2 + M \rightarrow O_2 + M$	Reverse reaction rate	
9	$O + O_2 + M \rightarrow O_3 + M$	$1.87 \times 10^{16} \times T_{gas}^{-1.1} \times \exp(7.89 \times 10^2 / R \times T_{gas})$<br>$O_2$ / O_2(a ^{1}) / O_2(b ^{1}) / O_2(c) enhanced by 1.25<br>O_3 enhanced by 3.125	[5]

		O / O(^{1}D) / O(^{1}S) enhanced by 5.0	
		CO enhanced by 0.25[#]	
		CO_2 enhanced by 0.5[#]	
10	$O_3 + M \rightarrow O + O_2 + M$	Reverse reaction rate	
11	$O_3 + O \rightarrow O_2 + O_2$	$1.29 \times 10^{13} \times \exp(4.560 \times 10^3 / R \times T_{gas})$	[5]
12	$O_2 + O_2 \rightarrow O_3 + O$	Reverse reaction rate	
13	$C + CO + M \rightarrow C_2O + M$	2.357×10^{16}	[26]
14	$C_2O + M \rightarrow C + CO + M$	Reverse reaction rate	
15	$C_2O + O \rightarrow CO + CO$	5.0×10^{13}	[26]
16	$CO + CO \rightarrow C_2O + O$	Reverse reaction rate	
17	$C_2O + O_2 \rightarrow CO_2 + CO$	1.99×10^{11}	[26]
18	$CO_2 + CO \rightarrow C_2O + O_2$	Reverse reaction rate	
19	$O_3 + CO \rightarrow CO_2 + O_2$	0.24	[47]
20	$CO_2 + O_2 \rightarrow O_3 + CO$	Reverse reaction rate	

[#] The efficiencies were found by iterative trial and error method.

6. Mechanism comparison

To understand how our mechanism compares with respect to existing mechanisms, we compared the reaction rates of the major reactions that were outlined by the chemical analysis. We compared our present work to that of Kozák et al. [29], Berthelot et al. [48], Koelman et al. [49], Wang et al. [28], Vermeiren et al. [50] and Biondo et al. [27].

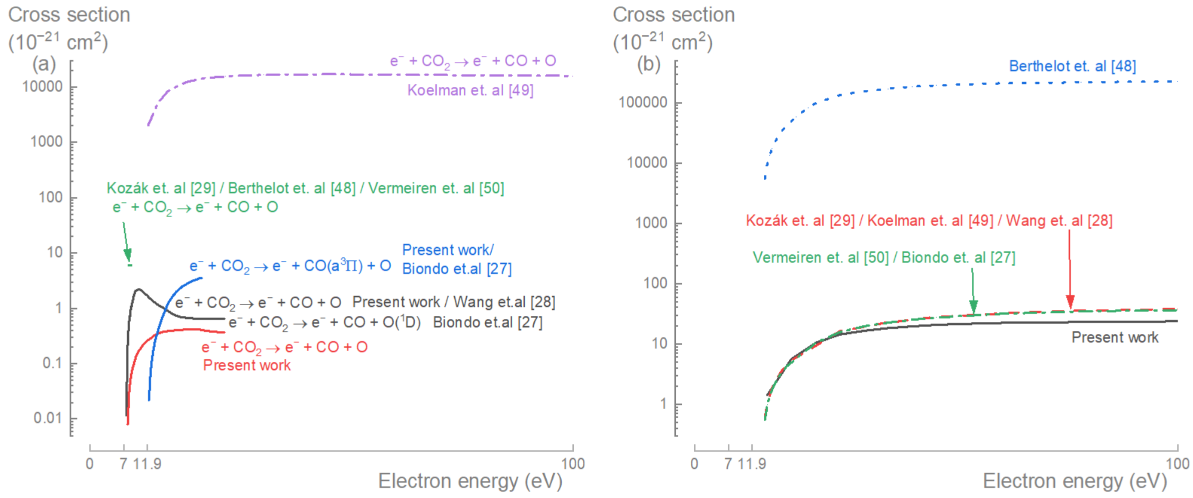

Fig. S22 Comparison of cross-sections used for (a) dissociation and (b) ionization reaction $e^- + CO_2 \rightarrow e^- + e^- + CO_2^+$.

The dissociation cross-section used in [29], [48] and [50] belong to the work of Phelps [19]. This cross-section overestimates dissociation especially at low E/N values [53] and will be evident between 300 and 440 K. The cross-section for dissociation used in [49] is higher in magnitude by an order of 4. The cross-section used in [28] is similar to ours. [29] is the only work which matches the cross-section used in our present work and also has the species 'CO(a $^3\Pi$)'. With regards to the cross-section used for ionization to CO_2^+, [48] has a much higher cross-section value as compared to others.

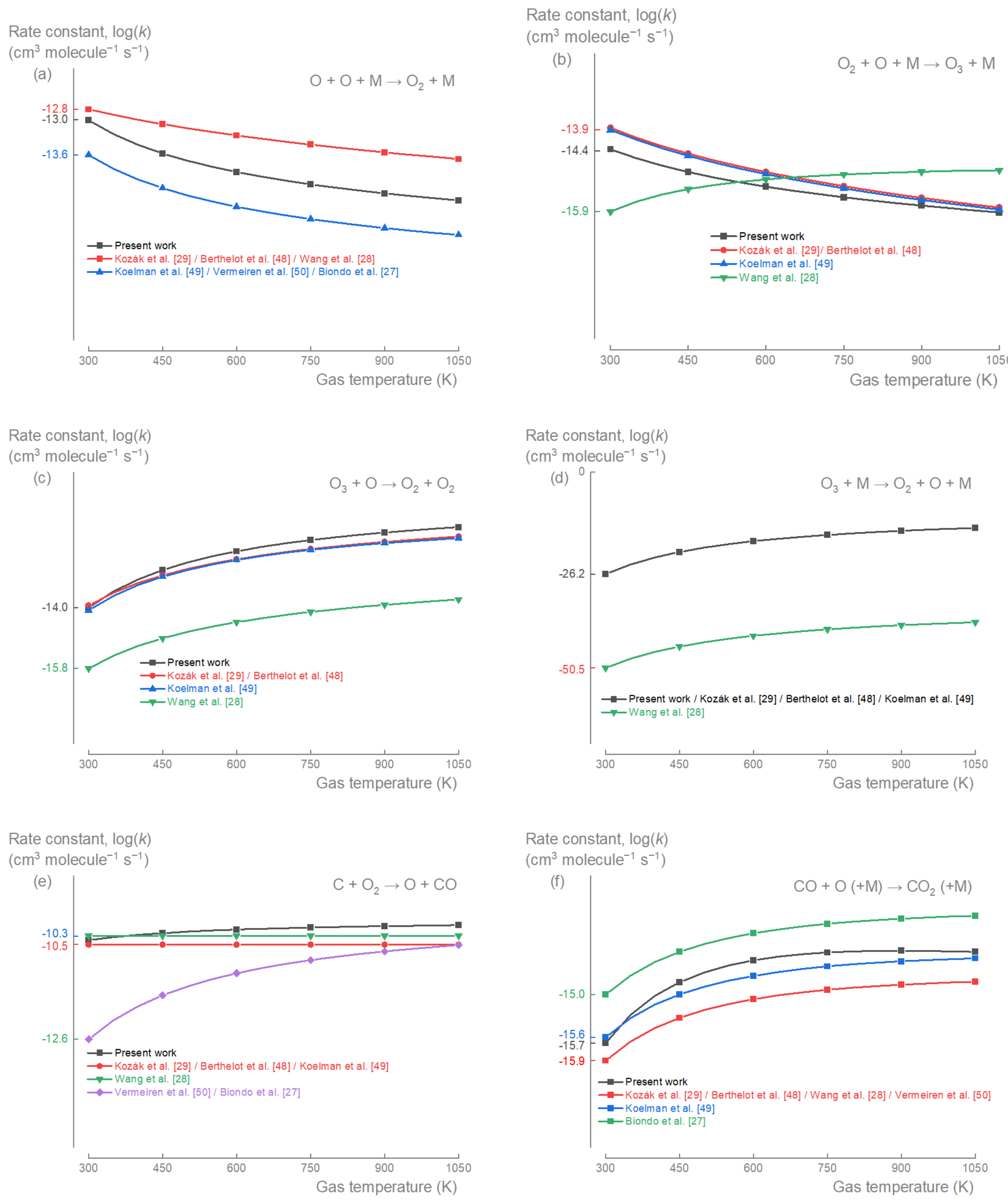

Fig. S23 Comparison of variation in reaction rate constant (k) with gas temperature (T_g) for main reactions that determine plasma-based CO_2 conversion process (a) $O + O + M \rightarrow O_2 + M$ (b) $O_2 + O + M \rightarrow O_3 + M$ (c) $O_3 + O \rightarrow O_2 + O_2$ (d) $O_3 + M \rightarrow O_2 + O + M$ (e) $C + O_2 \rightarrow O + CO$ (f) $CO + O\ (+M) \rightarrow CO_2\ (+M)$ of our present work with that of Kozák et al. [29], Berthelot et al. [48], Koelman et al. [49], Wang et al. [28], Vermeiren et al. [50] and Biondo et al. [27].

When comparing the thermal reaction rate constants given in figure S23, [29] and [48] use similar rates for all the reactions considered. The only difference in the reaction rates among the major reactions is the higher cross-section for ionization used by [48]. Due to the higher reaction rate constants of O + O + M → O_2 + M (R10) and O_2 + O + M → O_3 + M (R11) and lower CO + O (+M) → CO_2 (+M) (R6) values, these mechanisms are expected to overpredict the conversion especially for temperatures below 600 K as seen in section 1.2.4. The reaction rates of [49] for (R10) is lower than that in our work while that of (R11) is slightly higher with comparable values for (R6). With the much higher dissociation cross-section, [49] will presumably overpredict the conversion. With a higher reaction rate constant for (R10), an entirely different trend for (R11) and a lower rate for (R6), [28] is anticipated to not just overpredict the conversion but also could have an entirely different trend altogether. Both [27] and [50] omits chemistry regarding O_3, which from the chemical analysis and experiment is an essential intermediary and hence these chemistry sets could possibly have very different trends than those we observed.

7. References

[1] Cha, M. S. & Snoeckx, R. Plasma Technology–Preparing for the Electrified Future. Front. Mech. Eng. 8, 1–5 (2022).

[2] Pipa, A. V. & Brandenburg, R. The Equivalent Circuit Approach for the Electrical Diagnostics of Dielectric Barrier Discharges: The Classical Theory and Recent Developments. Atoms 7, 14 (2019).

[3] Manley, T. C. The Electric Characteristics of the Ozonator Discharge. Trans. Electrochem. Soc. 84, 83–96 (1943).

[4] Peeters, F. & Butterworth, T. Electrical diagnostics of dielectric barrier discharges. In Atmospheric Pressure Plasma-from Diagnostics to Applications Ch.2 (IntechOpen eBooks, 2019).

[5] Bang, S., Snoeckx, R. & Cha, M. S. Temperature-Dependent Kinetics of Ozone Production in Oxygen Discharges. Plasma Chem. Plasma Process. 43, 1453–1472 (2023).

[6] Snoeckx, R., Jun, D., Lee, B. J. & Cha, M. S. Kinetic study of plasma assisted oxidation of H2 for an undiluted lean mixture. Combust. Flame 242, 112205 (2022).

[7] Pancheshnyi, S., Eismann, B., Hagelaar, G. & Pitchford, L. ZDPlasKin: a new tool for plasmachemical simulations. Bull. Am. Phys. Soc. 53 (2008).

[8] Kee, R. J., Rupley, F. M., Meeks, E. & Miller, J. A. CHEMKIN-III: A FORTRAN chemical kinetics package for the analysis of gas-phase chemical and plasma kinetics. Sandia National Lab. (SNL-CA), Livermore, CA (United States); (1996).

[9] Hagelaar, G. J. M. & Pitchford, L. C. Solving the Boltzmann equation to obtain electron transport coefficients and rate coefficients for fluid models. Plasma Sources Sci. Technol. 14, 722–733 (2005).

[10] Snoeckx, R., Aerts, R., Tu, X. & Bogaerts, A. Plasma-Based Dry Reforming: A Computational Study Ranging from the Nanoseconds to Seconds Time Scale. J. Phys. Chem. C 117, 4957–4970 (2013).

[11] Snoeckx, R., Heijkers, S., Van Wesenbeeck, K., Lenaerts, S. & Bogaerts, A. CO2 conversion in a dielectric barrier discharge plasma: N2 in the mix as a helping hand or problematic impurity? Energy

Environ. Sci. 9, 999–1011 (2016).

[12] Snoeckx, R., Setareh, M., Aerts, R., Simon, P., Maghari, A. & Bogaerts, A. Influence of N2 concentration in a CH4/N2 dielectric barrier discharge used for CH4 conversion into H2. Int. J. Hydrogen Energy 38, 16098–16120 (2013).

[13] Motret, O., Pellerin, S., Nikravech, M., Massereau, V. & Pouvesle, J.-M. Spectroscopic characterization of CH4+CO2 plasmas excited by DBD at atmospheric pressure. Plasma Chem. Plasma Process. 17, 393–407 (1997).

[14] Motret, O., Hibert, C., Pellerin, S. & Pouvesle, J.-M. Rotational temperature measurements in atmospheric pulsed dielectric barrier discharge - gas temperature and molecular fraction effects. J. Phys. D Appl. Phys. 33, 1493–1498 (2000).

[15] Aerts, R., Somers, W. & Bogaerts, A. Carbon Dioxide Splitting in a Dielectric Barrier Discharge Plasma: A Combined Experimental and Computational Study. ChemSusChem 8, 702–716 (2015).

[16] Sun, S. R., Wang, H. X. & Bogaerts, A. Chemistry reduction of complex CO2 chemical kinetics: application to a gliding arc plasma. Plasma Sources Sci. Technol. 29, 025012 (2020).

[17] Alves, L. L. IST-Lisbon database, www.lxcat.net, retrieved on March 16, 2023.

[18] Lowke, J. J., Phelps, A. V. & Irwin, B. W. Predicted electron transport coefficients and operating characteristics of CO2–N2–He laser mixtures. J. Appl. Phys. 44, 4664–4671 (1973).

[19] Phelps, A. V. Phelps database, www.lxcat.net, retrieved on March 16, 2023.

[20] Straub, H. C., Lindsay, B. G., Smith, K. A. & Stebbings, R. F. Absolute partial cross sections for electron-impact ionization of CO2 from threshold to 1000 eV. J. Chem. Phys. 105, 4015–4022 (1996).

[21] Mangan, M. A., Lindsay, B. G. & Stebbings, R. F. Absolute partial cross sections for electron-impact ionization of CO from threshold to 1000 eV. J. Phys. B At. Mol. Opt. Phys. 33, 3225–3234 (2000).

[22] Straub, H. C., Renault, P., Lindsay, B. G., Smith, K. A. & Stebbings, R. F. Absolute partial cross sections for electron-impact ionization of H2, N2, and O2 from threshold to 1000 eV. Phys. Rev. A 54, 2146 (1996).

[23] Polak, L. S. & Slovetsky, D. I. Electron impact induced electronic excitation and molecular dissociation. Int. J. Radiat. Phys. Chem. 8, 257–282 (1976).

[24] Tsang, W. & Hampson, R. F. Chemical Kinetic Data Base for Combustion Chemistry. Part I. Methane and Related Compounds. J. Phys. Chem. Ref. Data 15, 1087–1279 (1986).

[25] Yujie, T., G. P. S. Foundational fuel chemistry model 1.0. Stanford University Available at: https://web.stanford.edu/group/haiwanglab/FFCM1/pages/download.html. (Accessed: 16th March 2023).

[26] Cenian, A., Chernukho, A., Borodin, V. & Śliwiński, G. Modeling of Plasma-Chemical Reactions in Gas Mixture of CO2 Lasers I. Gas Decomposition in Pure CO2 Glow Discharge. Contrib. Plasma Phys. 34, 25–37 (1994).

[27] Biondo, O., Fromentin, C., Silva, T., Guerra, V., Van Rooij, G. & Bogaerts, A. Insights into the limitations to vibrational excitation of CO2: validation of a kinetic model with pulsed glow discharge experiments. Plasma Sources Sci. Technol. 31, 074003 (2022).

[28] Wang, W., Snoeckx, R., Zhang, X., Cha, M. S. & Bogaerts, A. Modeling Plasma-based CO2 and CH4 Conversion in Mixtures with N2, O2, and H2O: The Bigger Plasma Chemistry Picture. J. Phys. Chem. C 122, 8704–8723 (2018).

[29] Kozák, T. & Bogaerts, A. Splitting of CO2 by vibrational excitation in non-equilibrium plasmas: a reaction kinetics model. Plasma Sources Sci. Technol. 23, 045004 (2014).

[30] Pokrovskiy, G. V., Popov, N. A. & Starikovskaia, S. M. Fast gas heating and kinetics of electronically excited states in a nanosecond capillary discharge in CO2. Plasma Sources Sci. Technol. 31, 035010 (2022).

[31] Berenguer, C. & Katsonis, K. Global Modeling of CO2 Discharges with Aerospace Applications. Adv. Aerosp. Eng. 2014, 1–17 (2014).

[32] Wang, W., Berthelot, A., Kolev, S., Tu, X. & Bogaerts, A. CO2 conversion in a gliding arc plasma: 1D cylindrical discharge model. Plasma Sources Sci. Technol. 25, 065012 (2016).

[33] Morgan, L. W. Morgan database, www.lxcat.net, retrieved on March 16, 2023.

[34] Alves, L. L. The IST-Lisbon database on LXCat. J. Phys. Conf. Ser. 565, 012007 (2014). https://doi.org/10.1088/1742-6596/565/1/012007

[35] Itikawa, Y. Cross Sections for Electron Collisions with Oxygen Molecules. J. Phys. Chem. Ref. Data 38, 1–20 (2008).

[36] Biagi, S. F. Biagi database (Fortran program MAGBOLTZ). www.lxcat.net/Biagi, retrieved on March 16, 2023.

[37] Capitelli, M., Ferreira, C. M., Gordiets, B. F. & Osipov, A. I. Plasma Kinetics in Atmospheric Gases. (Springer Science & Business Media, 2013).

[38] Kochetov, I. TRINITI database. www.lxcat.net/TRINITI, retrieved on March 16, 2023.

[39] Suzuki, D. et al. Electron excitation of the Schumann–Runge continuum, longest band, and second band electronic states in O2. J. Chem. Phys. 134, 084308 (2011).

[40] Liu, D. X., Bruggeman, P., Iza, F., Rong, M. Z. & Kong, M. G. Global model of low-temperature atmospheric-pressure He + H2O plasmas. Plasma Sources Sci. Technol. 19, 025018 (2010).

[41] Kossyi, I. A., Kostinsky, A. Y., Matveyev, A. A. & Silakov, V. P. Kinetic scheme of the non-equilibrium discharge in nitrogen-oxygen mixtures. Plasma Sources Sci. Technol. 1, 207–220 (1992).

[42] Van Gaens, W. & Bogaerts, A. Kinetic modelling for an atmospheric pressure argon plasma jet in humid air. J. Phys. D Appl. Phys. 46, 275201 (2013).

[43] Atkinson, R. et al. Evaluated kinetic and photochemical data for atmospheric chemistry: Part 1 gas phase reactions of Ox, HOx, NOx and SOx species. Atmos. Chem. Phys. Discuss. 3, 6179–699 (2003).

[44] Vasiljeva, A. N. et al. On the possibility of O2(a 1Δg) production by a non-self-sustained discharge for oxygen–iodine laser pumping. J. Phys. D Appl. Phys. 37, 2455–2468 (2004).

[45] Baulch, D. L. et al. Evaluated Kinetic Data for Combustion Modeling: Supplement II. J. Phys. Chem. Ref. Data 34, 757–1397 (2005).

[46] Troe, J. Thermal dissociation and recombination of polyatomic molecules. Symp. Int. Combust. Proc. 15, 667–680 (1975).

[47] Arin, L. M. & Warneck, P. Reaction of ozone with carbon monoxide. J. Phys. Chem. 76, 1514–1516 (1972).

[48] Berthelot, A. & Bogaerts, A. Modeling of CO2 Splitting in a Microwave Plasma: How to Improve the Conversion and Energy Efficiency. J. Phys. Chem. C 121, 8236–8251 (2017).

[49] Koelman, P. et al. A Comprehensive Chemical Model for the Splitting of CO2 in Non-Equilibrium Plasmas. Plasma Process. Polym. 14, e1600154 (2017).

[50] Vermeiren, V. & Bogaerts, A. Plasma-Based CO2 Conversion: To Quench or Not to Quench? J. Phys. Chem. C 124, 18401–18415 (2020).
